# Mulberry (*Morus alba*) leaves in sheep and goat nutrition: a critical narrative review of rumen function, performance, meat quality, and mechanistic evidence

**DOI:** 10.3389/fvets.2026.1986200

**Published:** 2026-10-12

**Authors:** Muhammad Jawad, Pengyu Zhang, Haoqi Yang, Jianguo Shi, Linfeng Mi, Yanyi He, Sina Lu, Pengpeng Fu, Huzaifa Iftikhar, Xiaoyue Song

**Affiliations:** 1 School of Modern Agriculture, Yulin University, Yulin, China; 2 Yulin City Food Inspection and Testing Center, Yulin, China

**Keywords:** alternative feed, carcass traits, mulberry, muscle composition, oxidative status, rumen microbiota, sensory quality, small ruminants

## Abstract

Mulberry (*Morus alba*) leaves combine conventional nutrients with polyphenols and flavonoids and are increasingly evaluated as alternative feeds for sheep and goats. This critical narrative review examines how feed form, processing, inclusion level, replacement strategy, and dietary balance influence nutrient utilization, rumen function, productive performance, carcass traits, and meat quality. The review question, eligibility criteria, evidence hierarchy, and synthesis framework were defined and approved by the authors. Relevant literature was identified and verified across bibliographic databases, publisher and journal platforms, Chinese-language sources, regional scholarly indexes, and backward/forward citation chaining, with direct sheep and goat feeding trials assigned the greatest evidential weight. Mulberry generally maintains intake and growth when complete diets remain nutritionally adequate, whereas excessive replacement of nutrient-dense concentrate can depress performance. Carcass yield is commonly maintained. Several independent trials demonstrate diet-sensitive tenderness, water retention, muscle fatty-acid profiles, and amino-acid composition, although sensory and physicochemical responses remain heterogeneous. Rumen fermentation, microbial-community shifts, antioxidant responses, and tissue lipid or gene-expression pathways provide mechanistic plausibility, but microbiome and omics associations are not treated as causal unless mediation was directly tested. Mulberry should therefore be treated as a context-dependent feed resource rather than a universally beneficial additive.

## 1 Introduction

Small-ruminant production converts forages and crop resources into high-value meat, but growth, carcass composition and eating quality remain strongly responsive to feeding system and nutrient supply. Across sheep and goats, diet can alter average daily gain, dressing performance, intramuscular fat, color, tenderness, fatty-acid composition and sensory attributes (1–3). Goat meat is generally lean, but its quality varies with breed, age, production system and diet (4). Alternative feed resources are economically and environmentally relevant when they reduce dependence on conventional concentrates, make productive use of locally available or climate-resilient biomass, and maintain animal performance. Sorghum, bitter vetch, tannin-containing forages and Azolla are examples of alternative resources evaluated in small-ruminant systems (5–8). Their practical value depends on nutrient balance, preservation losses, local availability and cost. Against this broader alternative-feed context, mulberry requires evaluation not only for its ability to maintain intake and growth but also for its downstream effects on carcass value and meat quality.

Mulberry (*Morus alba*) is a woody forage containing appreciable protein, digestible nutrients, minerals, and polyphenolic and flavonoid constituents that may influence redox status, microbial ecology, and metabolism (9, 10). Here, “functional feed” means a feed that supplies conventional nutrients while also delivering bioactive constituents with potential biological effects. Nutritional replacement effects and putative phytochemical effects are therefore evaluated separately rather than assuming that every response is bioactive in origin.

The term “mulberry feed” covers nutritionally non-equivalent materials, including fresh or dried leaves, leaf meal or powder, hay, silage, pelleted or fermented total mixed rations (TMR), pomace and purified flavonoid preparations. Foundational sheep and goat studies showed that mulberry can support substantial intake and digestibility and can replace part of conventional protein or forage resources (11–15). Drying, ensiling, fermentation and pelleting can change moisture, fiber accessibility, fermentation products, microbial exposure and phytochemical stability; feed form is therefore treated as a potential effect modifier rather than a preparation detail. More recent work has expanded to rumen microbiota, antioxidant systems, carcass traits, meat composition and multi-omics endpoints (16–22).

Recent syntheses address adjacent but broader questions. Hassan et al. (9) reviewed mulberry biomass and flavonoids across ruminants, Wang et al. (10) surveyed Morus-derived products across animal production, Geng et al. (23) quantitatively synthesized growth, production, gut-microbiota and immune outcomes across poultry and livestock, and Rohela et al. (24) reviewed mulberry as a forage crop across livestock systems. These syntheses are valuable but do not jointly resolve small-ruminant meat outcomes, dose denominators, shared cohorts, terminal biological replication, tissue specificity, and the distinction between measured phenotype, association and mechanistic hypothesis. Two 2026 reviews further emphasize species and ingredient specificity: Liu et al. (25) focused on mulberry-leaf flavonoids and juvenile animal health, whereas Ma et al. (26) evaluated paper mulberry (*Broussonetia papyrifera*), a botanically distinct feed resource. Only publications publicly available by the final literature-update date of 15 August 2026 were eligible for the final evidence set. Throughout this review, *B. papyrifera* is excluded from the *M. alba* evidence base and is mentioned only to distinguish adjacent literature. The present review therefore addresses the narrower unresolved question of how *M. alba* feeding in sheep and goats connects feed chemistry and processing with rumen function, systemic physiology, carcass development, muscle composition, flavor and sensory evidence.

Accordingly, the review asks four questions: under which feeding conditions can mulberry replace conventional ingredients without loss of performance; which carcass and meat-quality responses are reproducible; which rumen, redox and metabolic mechanisms are directly supported; and which design features explain inconsistent findings.

## 2 Review methodology

### 2.1 Review design and literature identification

This article was developed as a structured critical narrative review rather than a registered systematic review or meta-analysis. No prospective registered protocol or formal duplicate-screening/adjudication procedure was used. Instead, the authors defined and approved the review question, species and ingredient scope, eligibility criteria, evidence hierarchy, data-extraction domains, and rules used to distinguish direct evidence, supportive association, and mechanistic hypothesis. The review was designed to critically synthesize evidence on *Morus alba* feeding in sheep and goats, with particular emphasis on the feed-to-rumen-to-performance-to-meat pathway. A final literature update was completed through 15 August 2026, and only records publicly available by that date were included in the final evidence set.

Literature identification and source verification used distinct source categories: bibliographic databases/biomedical indexes (PubMed, PubMed Central and Europe PMC); publisher or journal platforms (including ScienceDirect, MDPI, Wiley, Springer, Frontiers, Taylor & Francis and PeerJ); Chinese-language and regional journal sources (including Wanfang Data and journal portals); and supplementary scholarly indexes used to locate older or inconsistently indexed records (including CiNii, KoreaScience and Semantic Scholar). Searches had no lower publication-date limit and were updated iteratively during manuscript development, with the final update on 15 August 2026. Exact-title, author, DOI and bibliographic cross-checking, together with backward and forward citation chaining, was used to identify foundational studies, companion publications and newly published work. The recurring English concept blocks combined *Morus alba*/mulberry leaf/mulberry silage with sheep/goat/lamb/ovine/caprine/small ruminant and outcome terms for feed, digestibility, rumen fermentation, microbiota, growth, carcass, meat quality, fatty acids, amino acids, flavor, antioxidant responses and omics; equivalent Chinese-language concepts were used for Chinese records. Because the searches were iterative for a critical narrative review, database-specific hit counts and exact historical Boolean strings were not prospectively archived and are not reconstructed retrospectively. Table 1 distinguishes the source categories, and Supplementary Table S2 documents the search concepts, date limits and source-verification procedure used for reproducibility.

**Table 1:** Literature-identification sources and source-verification framework used for the critical narrative review.

Literature-identification pathway	Sources used	Role in the review	Verification/selection rule
Bibliographic databases and biomedical indexes	PubMed; PubMed Central (PMC); Europe PMC	Identify and verify internationally indexed primary and review literature; confirm titles, abstracts, authorship and identifier information	Prioritize direct sheep/goat feeding interventions; use indexed records to verify study identity and reported design details
Publisher and journal platforms	ScienceDirect; MDPI; Wiley; Springer; Frontiers; Taylor & Francis; PeerJ; other original publisher/article pages	Retrieve full text or authoritative source metadata and check treatments, sample information, outcomes and references	Prefer the original publication or publisher record over secondary summaries
Chinese-language, regional and scholarly-index sources	Wanfang Data; Chinese journal websites and journal portals; CiNii; KoreaScience; Semantic Scholar; Academic Journals and related scholarly-index pages	Locate Chinese-language, regional, older or inconsistently indexed studies and verify bibliographic details	Include evidence only when study design and reported outcomes can be checked from an accessible primary or authoritative source record
Citation chaining and bibliographic verification	Backward/forward citation tracing from key primary studies and reviews; exact-title, author and DOI follow-up	Identify foundational studies, companion publications and newer directly relevant work through 15 August 2026	Flag shared cohorts; distinguish direct sheep/goat evidence from supporting cross-ruminant, feed-level or mechanistic evidence

### 2.2 Eligibility criteria and evidence hierarchy

Peer-reviewed studies were prioritized when *M. alba* leaves or derived feed materials were administered directly to sheep or goats. Direct-evidence exclusions included paper mulberry (*B. papyrifera*), interventions in which the *Morus* species or treatment could not be verified, records without sufficient design or outcome information to check the claim, and studies whose endpoints were unrelated to the review questions. Supporting cattle or buffalo studies were retained only when they clarified conserved rumen, preservation or processing mechanisms; feed-level and in vitro studies were retained only for preservation, degradation or fermentation questions; non-ruminant studies were not used as direct evidence for sheep/goat conclusions. Reviews were used for context and citation discovery rather than as substitutes for direct evidence. No formal language restriction was applied, but non-English primary studies were retained only when an English-language abstract or sufficiently detailed bibliographic record allowed the design and reported outcomes to be checked. This requirement may introduce language or reporting bias and is considered when interpreting the evidence. Companion publications from shared animal cohorts were flagged so that publication counts were not treated as independent biological replication.

### 2.3 Data extraction and evidence synthesis

A 15-domain extraction framework recorded animal model and biological sample size, experimental unit, diet and mulberry form, inclusion or replacement basis, duration, intake and digestibility, rumen variables, growth, carcass and meat traits, fatty acids, amino acids, flavor or sensory outcomes, antioxidant or physiological responses, proposed mechanisms, and major sources of bias. Extraction and evidence appraisal were not conducted as blinded independent duplicate review, and this is acknowledged as a methodological limitation. Evidence was weighted at the claim level using five explicit dimensions: directness (species and intervention relevance to sheep/goat *M. alba* feeding), cohort independence (whether publications represented distinct animal experiments), biological replication (animal/pen experimental unit and terminal biological n), intervention comparability (feed form, dose denominator, replaced ingredient and nutritional matching), and endpoint proximity (direct phenotype vs. proxy, correlation, predicted function or pathway enrichment). These rules distinguish direct evidence, supportive association and mechanistic hypothesis rather than assigning a formal numerical risk-of-bias score.

Feasibility of quantitative pooling was considered for repeatedly reported outcomes. The final evidence matrix contains 67 publication-level records; 43 are direct small-ruminant records when direct whole-feed, purified-compound and companion publications are counted at the publication level, while the remainder provide review, feed-level, supporting-ruminant or indirect context. The manuscript contains 71 references in total; references 5–8 are used only for broader alternative-feed context and are therefore not included in the mulberry-focused evidence matrix. The number of independent experiments is smaller because several publications share cohorts. After collapsing known companion cohorts and examining exposure definition, comparative diets, experimental units, terminal sampling and outcome reporting, no major endpoint had a sufficiently large set of independent, nutritionally comparable contrasts with compatible effect measures to support a robust pooled estimate. A meta-analysis was therefore not undertaken. The synthesis instead emphasizes consistency, moderators, contradictory findings and claim-level evidence strength. Representative direct small-ruminant studies are summarized in Table 2, and the study-level matrix identifies cohort overlap and terminal sample size where available.

**Table 2:** Representative direct small-ruminant studies across major feed forms, dose designs, and evidence domains.

Study	Animal model	Mulberry strategy	Main evidence domain	Critical appraisal
Doran et al. (12)	Wethers; two sheep trials	Dried mulberry foliage vs. alfalfa/oat hay	Digestibility/protein value	Direct foundational forage comparison
Ouyang et al. (20, 21)	Male Hu sheep, *n* = 40 (8/treatment); terminal meat/liver *n* = 3/treatment	Leaf powder; 0%−60% of concentrate (not treated as total-diet DM)	Rumen, growth, meat, antioxidant genes	Companion publications from one 40-lamb experiment; terminal meat *n* = 3/group
Sun et al. (37)	Small-tailed Han sheep, *n* = 32 (8/treatment)	0, 8, 24 or 32% of ration (air-dry basis) replacing wildrye	Digestibility, growth, carcass	Direct forage replacement; animal-level *n* = 8/treatment
Wang et al. (22)	White Cashmere goats, *n* = 60 (10/treatment); terminal *n* = 3/treatment	Ensiled 10-20% vs sun-dried 10-15% in TMR	Rumen microbiota, serum	Direct feed-form comparison; terminal *n* = 3/group
Han et al. (38); M. Wang et al. (58)	Hu lambs, n=96; 6 replicate pens/treatment x 4 lambs	0, 20, 40 or 60% maize-silage replacement with concurrent soybean-meal adjustment	Growth, rumen, carcass/meat	Shared dietary strategy/companion outcomes; pen replication should be distinguished from terminal n
Long et al. (18)	Crossbred black goats, *n* = 44 (11/treatment); terminal *n* = 5/treatment	40% mulberry in pelleted, fresh or fermented TMR	Growth, meat, FA/AA, genes	Direct processing comparison; terminal tissue *n* = 5/group
Cirne et al. (61, 62)	Ile-de-France lambs, *n* = 24 (8/treatment)	0, 12.5 or 25% mulberry hay on total-diet DM basis	Meat, sensory, muscle FA	Two papers from one cohort; n=8/treatment
Wang and Luo (33)	Tan lambs, *n* = 40 (20/diet); terminal *n* = 6/diet	20% mulberry leaf silage of diet DM replacing 20% alfalfa silage	Microbiota, antioxidant/immune	Microbe-host links are correlational; terminal n=6/group
Luo et al. (19)	Xiangdong black goats, *n* = 25 (5/treatment)	0, 5, 10, 15 or 20% leaf powder on air-dry diet basis	Growth, meat, FA/AA	High relevance; n=5/group
Deng et al. (48)	Male Hu sheep, *n* = 120; 6 replicate groups/treatment x 5 sheep	0, 10, 20 or 30% silage mulberry in pelleted TMR	Growth, meat quality, antioxidant, rumen fermentation	Large feeding-phase enrollment; terminal meat n not confirmed in accessible record
Cui et al. (16, 17)	Growing sheep, *n* = 54 (18/group); slaughter *n* = 6/group; omics subsets ~5–8	Fermented vs dried leaves; reported diet inclusions differ (FML 16.59%, DML 7.11%)	Antioxidant, transcriptomics, metabolomics/lipidomics	Companion papers, one experiment; processing not isolated under nutritionally identical exposure
Hou et al. (67)	Small-tailed Han sheep, *n* = 32 (8/treatment); terminal meat *n* = 4/treatment	Approx. 0, 8, 24 or 32% mulberry in complete diets combined with corn straw	Growth, water retention, antioxidant	Terminal meat/carcass n=4/group
Khattab et al. (39)	Xiangdong black wethers, *n* = 45 (15/treatment)	0, 50 or 100% soybean-meal protein replacement	Growth, carcass, muscle AA	Companion-like design with Cao et al. (69); duration internally inconsistent
Fan et al. (47)	Heat-stressed Hu sheep, *n* = 45 (~15/treatment)	0, 10 or 20% fermented-leaf concentrate replacement	Digestibility, rumen bacteria/fungi	Environmental context may modify response

AA, amino acids; FA, fatty acids; TMR, total mixed ration.

## 3 Mulberry feed characteristics, processing, and dietary use

The feeding value of mulberry foliage derives from both conventional nutrients and bioactive constituents, but composition varies with cultivar, maturity, leaf-to-stem ratio, harvest conditions and processing. Dried leaves and leaf meal are generally protein-rich relative to low-quality crop residues and show favorable degradability, yet crude-protein and fiber concentrations differ substantially among sources (13, 27–29). Doran et al. (12) found dried mulberry foliage broadly comparable with alfalfa for several digestibility and protein-value measures in sheep. Importantly, plant maturity itself changes feeding value: Saddul et al. (30) showed declining dry-matter and crude-protein disappearance in important plant fractions as mulberry matured. Table 3 summarizes representative nutritional characteristics, processing effects, phenolic markers and total-diet dry-matter inclusion values where the source permits a defensible conversion. Values are not pooled across forms because analytical basis and processing differ. Where directly comparable numerical values for dry matter, crude protein, fiber or degradability were unavailable, Table 3 states this explicitly rather than imputing or pooling across incompatible analytical bases.

**Table 3:** Representative nutritional characteristics, processing effects, phenolic markers, and inclusion bases of Morus alba feed forms.

Feed form	Representative nutrient/feeding characteristics	Degradability/ digestibility evidence	Representative inclusion basis	Processing/phenolic notes
Fresh/whole foliage	Directly comparable DM, crude-protein and fiber values were not consistently reported across the retained fresh-foliage studies; these values are therefore not pooled. Composition varies with maturity and leaf:stem ratio; direct retained studies support high palatability and useful protein value, but no single pooled DM/fiber/protein value is defensible.	Goat studies show form- and maturity-dependent intake/digestibility; digestibility declines as leaves mature.	Report original denominator unless total-diet DM can be derived; e.g., graded Guinea-grass replacement in the Nigerian study is a forage-replacement series, not a universal total-DM dose.	Fresh material is highly perishable; maturity is a major source of heterogeneity.
Dried leaf/hay/powder	Representative dried-leaf records report about 19.0%−20.1% crude protein and about 26.8%−27.4% NDF on a DM basis; values vary by source.	One feed-value study reported very high in-vitro true DM digestibility (~89.8%); sheep trials generally support favorable ruminal/total-tract use.	Examples with defensible total-diet basis include 0, 12.5 and 25% mulberry hay DM (Cirne cohort) and 0-15% dried leaves in Ossimi lamb diets. Concentrate-percentage studies are retained in their original denominator.	A chemically characterized dried-leaf preparation contained chlorogenic acid (3976 μg/g), gallic acid (1144), syringic acid (764), catechin (635), rutin (340), coumaric acid (120) and ellagic acid (90 μg/g).
Silage/ensiled leaves	Comparable DM, CP, NDF and ADF values were not consistently available across retained silage studies. Nutrient composition depends on crop material and ensiling. In one Hu-lamb comparison, mulberry silage supplied more CP and less fiber than maize silage.	20% mulberry leaf silage replacing alfalfa maintained DMI in Tan lambs; other silage trials show context-dependent fermentation/digestibility responses.	Examples include 20% of diet DM replacing alfalfa silage; 0%−30% silage mulberry in pelleted TMR; and ~53.2% of ration as either corn or mulberry silage in a direct comparison.	Ensiling changes organic acids and microbial ecology; additives can alter fermentation quality and aerobic stability.
Fermented/pelleted TMR	No single DM/CP/fiber value is presented because complete-TMR composition varies with formulation and processing. Processing can change particle size, feed sorting and nutrient availability; nominally equal mulberry percentages do not guarantee nutritionally identical diets.	At 40% mulberry TMR, pelleting increased apparent DM/CP digestibility and ADG relative to selected comparators in one goat study.	Long et al.: 40% mulberry in each experimental TMR; Cui cohort: fermented and dried diets used different inclusion percentages and therefore are not a nutritionally matched processing-only contrast.	Direct processing comparisons exist, but none isolates processing under perfectly matched nutrient and physical conditions.

Bioactive exposure is less standardized. Mulberry leaves contain polyphenols and flavonoids, and interventions using whole leaves, silage or extracted flavonoids have altered antioxidant indices, rumen microbial communities or antioxidant-related gene expression (21, 31–33). In one chemically characterized dried-leaf preparation, chlorogenic acid was the dominant quantified phenolic, accompanied by gallic acid, syringic acid, catechin, rutin, coumaric acid, and ellagic acid. However, most animal trials report the percentage of leaf biomass rather than the intake of defined marker compounds. Consequently, mechanistic claims should be attributed to specific phytochemicals only when the intervention or feed has been chemically characterized; otherwise the treatment represents a combined change in nutrients, physical structure and bioactives.

Processing creates a second major layer of heterogeneity. Ensiling can improve storage but changes organic acids, microbial ecology and potentially phytochemical stability. Glucose, lactic-acid bacteria and other additives alter fermentation quality, digestibility and aerobic stability of mulberry silage (34–36). Pelleting changes particle size, feed sorting and ruminal availability, while sun-drying differs from ensiling in moisture, fermentation products and microbial exposure. A few studies directly compare processing forms, including pelleted vs. fresh/fermented TMR (18), ensiled vs. sun-dried leaves (22), and fermented vs. dried leaves within the same sheep cohort (16, 17); however, these comparisons do not isolate processing under perfectly matched nutrient and physical conditions. Processing is therefore treated as a plausible effect modifier, not a proven causal explanation for every between-study difference.

Dose cannot be interpreted independently of replacement strategy. Where sufficient information was available, Table 3 expresses inclusion on a total-diet dry-matter basis; otherwise the original denominator is retained and explicitly labeled. Ouyang et al. (20) used 0–60% mulberry leaf powder in the concentrate of Hu sheep; Luo et al. (19) used 0%−20% leaf powder in pelleted goat diets; Sun et al. (37) replaced wild rye hay at 0%−32% of the ration; Han et al. (38) replaced maize silage with mulberry silage; and Khattab et al. (39) replaced soybean-meal protein rather than a fixed proportion of total dietary dry matter. These interventions are not biologically equivalent, so no generally applicable “low”, “moderate” or “high” inclusion range is inferred across incompatible denominators.

## 4 Intake, digestibility, and rumen function

Across direct sheep and goat studies, mulberry commonly maintained nutrient use when the complete ration remained nutritionally adequate, but the response depended on form, basal diet and replacement strategy. Foundational goat and sheep work showed acceptable intake, digestibility or nitrogen use with fresh/dried foliage, silage and protein replacement (11–15). More recent trials extend this pattern to whole mulberry, mulberry silage and crop-residue diets (40–43), with improved fiber utilization in some settings but not a consistent translation from digestibility to faster growth. This synthesis is therefore stronger for feasibility of nutrient substitution than for a universal digestibility advantage.

Dose-response evidence shows that nutritional constraints can become more apparent at high replacement levels. Ouyang et al. (20) found that 15%−30% mulberry leaf powder in concentrate maintained intake and gain, whereas 45%−60% reduced both. Higher fiber, lower energy density and changes in displaced concentrate composition are plausible explanations, but these mechanisms were not independently tested and should not be interpreted as demonstrated causes. In contrast, forage-replacement studies such as Sun et al. (37) and Han et al. (38) generally maintained intake and growth while altering selected digestibility and fermentation variables. Saleh et al. (44) reported improved digestibility and growth in Ossimi lambs receiving graded dried leaves, while the separate Damascus-buck digestibility experiment used only three bucks per treatment (45). At lower exposure, rumen-cannulated lambs receiving 2.5 or 5% white mulberry leaves showed changes in potentially degradable dry matter without a large shift in effective degradation (46), further indicating that ruminal responses may not follow a simple linear dose-response pattern. These contrasts support a non-linear, formulation-dependent response rather than a simple dose effect.

Rumen evidence confirms biological response beyond apparent digestibility, but not every microbial shift represents improved function. Across leaf powder, whole mulberry, silage, dried and fermented forms, studies reported changes in ruminal ammonia, volatile fatty acids, epithelial traits or microbial communities (20, 22, 33, 38, 40, 47). In White Cashmere goats, ensiled and sun-dried leaves altered community structure while conventional fermentation indices remained largely stable (22). In the 120-animal Hu-sheep trial, the experimental unit comprised six replicate groups per treatment with five sheep per replicate; the study reported higher intake/ADG at 30%, reduced cooking/drip losses at 20%−30%, higher T-AOC/SOD with lower MDA, and altered VFA responses (48). Because the terminal meat-sampling n was not confirmed in the accessible record, total enrollment is not used as a proxy for meat-quality replication. In Tan lambs, microbial changes correlated with host antioxidant and immune markers (33), but these correlations remain associative.

Purified-constituent, co-intervention and cross-ruminant evidence is kept separate from whole-feed sheep/goat evidence throughout this review. Purified mulberry-leaf flavonoids modified digestion, methane output and rumen microbial populations in sheep (31, 49), supporting phytochemical plausibility without representing a whole-leaf feeding contrast. Sahoo et al. (50) combined mulberry with Eupatorium, so the response cannot be attributed to mulberry alone. Cattle and buffalo studies using pellets, extracts or branch-and-leaf silage (32, 51–54) are used only to support conserved rumen or processing mechanisms and are not counted as direct small-ruminant meat evidence.

## 5 Growth performance and carcass characteristics

Growth performance is a primary test of whether an alternative feed can maintain nutrient supply and productive efficiency in small ruminants, and it should be interpreted together with intake, feed conversion and carcass endpoints (5, 6). Across the retained direct *M. alba* studies, responses are predominantly neutral or beneficial at nutritionally compatible inclusion, whereas adverse growth responses are concentrated in a smaller subset of high-replacement treatments. Tesfay et al. (55) and Sun et al. (37) reported no consistent depression in gain with substantial replacement. Whole mulberry or silage studies in Hu sheep generally maintained or supported growth (40, 41), while the Liuyang black-goat study reported improved feed efficiency and loin-eye area with pelleted leaf powder (56). These findings indicate a potential practical benefit when conventional feeds can be replaced without loss of performance; commercial benefit, however, requires direct feed-cost evidence.

High leaf-powder treatments in Ouyang et al. (20) significantly reduced intake and average daily gain at 45%−60% of concentrate, whereas 15%−30% maintained growth. The accompanying diet changes make nutrient dilution or altered energy/fiber supply plausible interpretations rather than demonstrated mechanisms. Other formulations produced different responses: pelleted 40% mulberry TMR increased digestibility and ADG relative to control/fresh-TMR comparisons in Long et al. (18); Luo et al. (19) reported a dose-related increase in ADG in Xiangdong black goats; and the 120-sheep pelleted-TMR trial reported significantly higher intake and ADG at 30% silage mulberry (48). The 160-sheep Ma et al. (57) study used four treatments with five pens per treatment and eight sheep per pen and reported maintained growth across 0%−45% silage replacement. These studies involve independent cohorts despite some shared breeds; differences are therefore discussed as formulation-specific responses rather than proof that nutrient equivalence or processing alone caused the outcome.

Carcass yield is more consistently maintained than improved. Tesfay et al. (55), Sun et al. (37), M. Wang et al. (58), Ma et al. (57) and Khattab et al. (39) reported broadly maintained slaughter or carcass performance under their tested formulations. Li et al. (56) reported improved loin-eye area together with economic outcomes in a 15-goat study divided among three treatments. Ouyang et al. (21) found no significant difference in hot carcass weight or dressing percentage, and terminal meat/liver outcomes used only three lambs per treatment. Cao et al. (59) reported significantly lower subcutaneous fat mass with intramuscular fat unchanged, but only five lambs per diet were slaughtered and proteomics used three biological replicates per treatment. Chen et al. (60) reported numerically greater carcass weight and loin-eye area, but neither difference was statistically significant and terminal n was three goats per group. These contrasts distinguish statistically supported changes from numerical differences and emphasize the limited terminal replication of several carcass studies.

The practical synthesis is therefore conditional: mulberry can maintain, and in some formulations improve, growth and carcass performance, but no universal growth-promoting dose can be recommended. Potential practical or commercial value depends on the ingredient replaced, achieved nutrient density, feed processing, breed, slaughter endpoint and local feed costs; only studies that directly measured economic outcomes are used as economic evidence.

## 6 Meat quality responses

### 6.1 Physicochemical traits

Physicochemical meat responses are more heterogeneous than production responses. Cirne et al. (61) found no significant changes in pH, color, water-holding capacity, cooking loss, shear force, sarcomere length or consumer acceptance with 0%−25% mulberry hay, supporting non-impairment rather than improvement. Ouyang et al. (21) reported significantly greater redness/chroma and lower shear force, but terminal meat measurements used only three lambs per treatment. Ma et al. (57) enrolled 160 Hu sheep in four treatments (five pens per treatment, eight sheep per pen) and reported lower cooking loss and shear force at selected silage levels; the feeding-phase enrollment should not be interpreted as the terminal meat n unless the sampled-animal number is reported. Likewise, the 120-sheep Deng et al. trial used six replicate groups per treatment with five sheep per replicate and reported lower cooking/drip losses at 20%−30%, but the accessible record did not confirm terminal meat-sampling *n*. These studies strengthen evidence for selected physicochemical responses while preserving uncertainty about terminal biological replication.

Goat evidence also demonstrates context dependence. Long et al. (18) compared 40% mulberry TMR forms and reported higher ADG with pelleted TMR, lower shear force and altered color relative to selected comparators, together with changes in muscle fatty acids, amino acids and IMP; only five goats per group were slaughtered for tissue collection. Luo et al. (19) reported dose-related growth and muscle-composition responses, with 15% significantly increasing several antioxidant indices and 20% lowering C18:0; five goats were assigned per treatment. Chen et al. (60) is an important counterexample: selenium-rich mulberry leaf powder significantly increased muscle water-loss rate, while 24-h pH, cooking loss and marbling were unchanged. Because selenium enrichment is a co-intervention and terminal n was three per group, this study is not interpreted as an effect attributable solely to mulberry.

### 6.2 Fatty-acid composition and nutritional quality

The clearest lipid-composition responses concern specific fatty acids rather than a uniform shift in total lipid classes. In the shared Cirne cohort, mulberry hay increased alpha-linolenic acid and eicosapentaenoic acid and reduced the n-6:n-3 ratio, while total SFA, MUFA and PUFA were not significantly changed (62); these changes are nutritionally relevant because they increase n-3 fatty-acid representation without demonstrating a universal improvement in all lipid indices. Long et al. (18), Wang et al. (58), Luo et al. (19, 63) and Ma et al. (57) provide additional direct muscle evidence. Ma et al. reported higher alpha-linolenic and arachidonic acids and lower total SFA at selected silage levels, while other fractions varied by dose. Where source papers analyzed many fatty acids, statistical significance is reported only as presented by the original authors; the review does not assume that nominal *P* values remained significant after multiplicity adjustment when such correction was not documented.

Tissue specificity is maintained throughout the synthesis, tables, abstract and conclusion. Cao et al. (59) reported lower saturated and trans fatty acids and higher alpha-linolenic and oleic acids in subcutaneous adipose tissue, not a conventional longissimus muscle profile; Cui et al. (17) likewise generated much of its lipidomic evidence from adipose tissue. These findings support altered lipid metabolism but are not presented as direct evidence of improved edible-muscle fatty-acid composition. Chen et al. (60) further illustrates heterogeneity: most muscle fatty acids and major lipid classes were unchanged, with only gamma-linolenic acid differing significantly and no significant improvement in the n-6:n-3 ratio.

Greater unsaturation may improve nutritional composition but can also increase susceptibility to oxidation. In the retained mulberry meat studies, fatty-acid shifts were more commonly measured than direct storage oxidative stability; several studies did not measure lipid-oxidation products or shelf-life endpoints. Accordingly, this review treats the oxidation trade-off as a mechanistic consideration supported by meat-science literature (64), not as a demonstrated consequence of every mulberry-induced fatty-acid change. Future studies should pair feed and muscle lipid profiles with rumen biohydrogenation markers, validated lipid-oxidation measurements and storage stability.

### 6.3 Amino acids, flavor, and sensory evidence

Direct evidence for quantitative muscle amino-acid modification is less extensive than evidence for growth or rumen outcomes but is available from several sheep and goat studies (18, 19, 39, 57, 63, 65). Ma et al. (57), for example, reported higher concentrations of several individual amino acids, total amino acids and flavor-related amino acids at selected silage levels. However, studies differ in whether amino acids are expressed as concentrations, proportions or other analytical bases, and differences in muscle dry matter or protein can influence apparent composition. The synthesis therefore interprets amino-acid changes within each study's reporting basis and avoids treating cross-study increases as a standardized absolute enrichment.

Flavor evidence is limited but methodologically informative. Wang et al. (66) used GC-MS and UHPLC-QTOF-MS to identify diet-related differences in hydrophilic metabolites and selected volatile or flavor-associated compounds in Tan-lamb meat. This demonstrates chemical modification, not sensory improvement: the study did not include trained sensory validation or aroma-activity analysis. Cirne et al. (61) provides direct consumer evidence and found no significant differences in flavor, tenderness or overall acceptance with mulberry hay. Accordingly, the manuscript uses “flavor-related compounds” or “flavor precursors” unless sensory or aroma-activity evidence directly supports perceived flavor change. Representative direct meat-quality responses are summarized in Table 4.

**Table 4:** Direction of meat-quality responses in representative direct studies.

Study	Physicochemical response	Composition/flavor response	Critical interpretation
Cirne et al. (61, 62)	Physicochemical and sensory traits largely unchanged	Selected n-3 FA increased; n-6:n-3 decreased	Shared 24-lamb cohort; *n* = 8/treatment; direct sensory non-impairment + selective n-3 lipid effect
Ouyang et al. (21)	Redness/chroma increased; shear force decreased	No full FA/AA/flavor profile	Statistically significant redness/shear-force changes, but terminal *n* = 3/group
Long et al. (18)	Meat traits varied with TMR processing	Muscle FA and 17 AA measured	Processing-associated response; terminal tissue *n* = 5/group and processing is not isolated from all diet differences
M. Wang et al. (58)	Generally maintained meat quality	Treatment-specific muscle composition shifts	Companion with silage feeding cohort
B. Wang et al. (66)	Raw-muscle metabolomics	Flavor/taste-related metabolites changed	*n* = 6/diet; chemical/metabolomic evidence without sensory or aroma-activity validation
Luo et al. (19)	Selected quality traits improved	Direct muscle FA and AA profiles	Direct goat evidence; *n* = 5/treatment; composition changes are not equivalent to sensory improvement
Hou et al. (67)	Water holding improved in selected groups	No FA/AA/volatile profiling	Selected water-retention effects; terminal meat/carcass *n* = 4/group
Ma et al. (57)	Cooking loss and shear force decreased	Muscle AA and unsaturated FA increased at selected levels	Feeding-phase *n* = 160 (5 pens/treatment x 8 sheep); terminal meat n not confirmed in accessible record
Chen et al. (60)	Water loss increased; 24-h pH and cooking loss unchanged	Most muscle FA unchanged	Terminal *n* = 3/group; selenium-rich mulberry is a co-intervention and cannot isolate mulberry alone
Luo et al. (63)	Direct goat muscle composition study	Fermented-leaf AA and FA profiles measured	Independent goat evidence; dose dependent; analytical expression should be retained as reported

AA, amino acids; FA, fatty acids.

## 7 Antioxidant, metabolic, and mechanistic evidence

Antioxidant and metabolic responses are among the most frequently measured mechanistic domains, but they are not treated as intrinsically stronger than direct rumen or phenotype evidence. Wang and Luo (33) reported higher serum catalase, glutathione peroxidase, superoxide dismutase and total antioxidant capacity with lower malondialdehyde in lambs fed mulberry leaf silage; terminal blood/rumen sampling used six lambs per group. Ouyang et al. (21) reported dose-related hepatic antioxidant-gene responses, but terminal liver/meat measurements used three lambs per treatment. Cui et al. (16) measured serum and muscle SOD, catalase, glutathione peroxidase, total antioxidant capacity and malondialdehyde, with six sheep per group slaughtered and smaller omics subsets. Other sheep/goat studies report additional antioxidant or physiological responses (39, 40, 45, 48, 67, 68). Purified mulberry flavonoids reduced methane output and altered protozoal/methanogen populations in sheep (49), demonstrating a ruminal response but not mediation of downstream meat quality.

The companion Cui et al. publications represent one 54-sheep experiment (18 sheep per group), with six sheep per group slaughtered and approximately 5–8 biological replicates used in different omics/biochemical assays. Cui et al. (16) combined measured serum and muscle antioxidant biomarkers with transcriptomics and targeted metabolomics; Cui et al. (17) extended the same cohort to adipose lipidomics, transcriptomics and biochemical markers. Differentially expressed genes/metabolites are distinguished from pathway-enrichment or association results, and the review does not infer multiple-testing significance beyond the procedures reported by the source papers. Similarly, Cao et al. (69) and Khattab et al. (39) use the same Xiangdong black-goat population size, initial body-weight range, three treatment groups (15 goats/group) and 0/50/100% soybean-protein replacement strategy; they are therefore treated conservatively as companion data sources rather than independent replication unless explicit cohort independence can be demonstrated.

An evidence-weighted pathway therefore runs from feed composition and processing to rumen nutrient use and microbial ecology, then to circulating redox and metabolic signals, tissue lipid/protein pathways and finally meat phenotype. Direct feeding/phenotype evidence supports the feed-to-intake/rumen and carcass/meat stages (for example 14, 16–18, 29, 33–35, 44, 54–59); microbiome-to-host and omics pathway transitions are mainly associative (16, 17, 33, 38, 49, 69); and the final transition from altered metabolites or antioxidant capacity to consumer-perceived flavor is least directly validated (61, 66, 70). Figure 1 therefore uses solid arrows for direct evidence, dashed arrows for associative/mechanistic links and dotted arrows for the least directly validated hypotheses.

**Figure 1:**
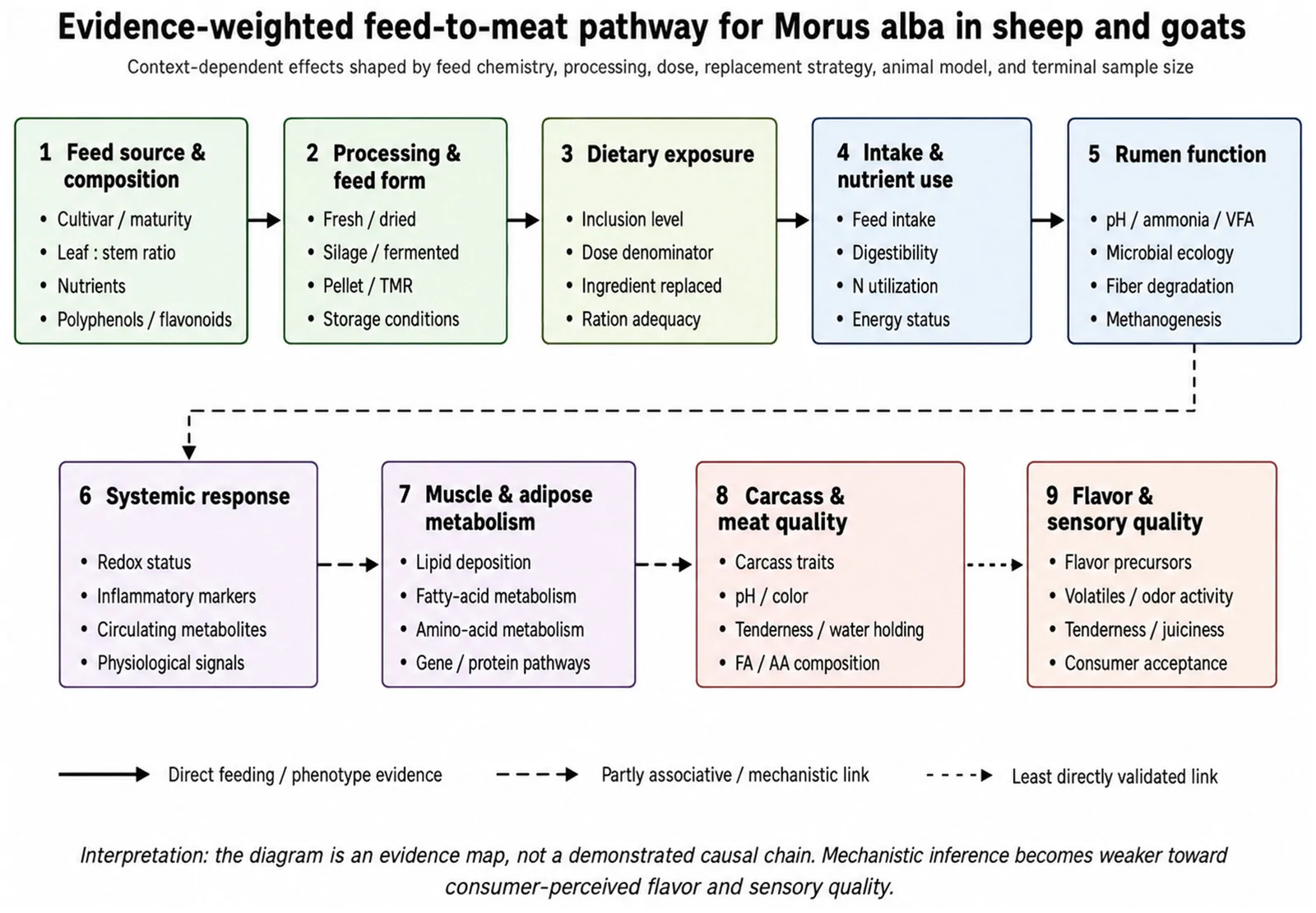
Evidence-weighted feed-to-meat pathway for Morus alba in sheep and goats. Solid arrows indicate stages supported by direct feeding or phenotype evidence; dashed arrows indicate mainly associative microbiome, biochemical, transcriptomic, metabolomic or lipidomic links; dotted arrows indicate the least directly validated hypotheses, particularly the transition to consumer-perceived flavor/sensory quality. Feed chemistry, processing, dose denominator, displaced ingredient, animal model and terminal sample size act as major moderators. The diagram is an evidence map, not a demonstrated causal chain.

## 8 Discussion

Two interpretive controversies run through the literature. First, favorable responses may reflect conventional nutrient substitution, specific actions of mulberry phytochemicals, or both; most whole-leaf feeding trials do not isolate these mechanisms experimentally. Second, nominal inclusion percentages are not directly comparable when the denominator, feed form and displaced ingredient differ. These issues explain part of the apparent inconsistency among studies, together with differences in study quality, animal characteristics, basal diets, analytical methods and terminal sample size.

### 8.1 Sources of response heterogeneity

Heterogeneity is a central biological result rather than statistical noise. Nominal inclusion percentages refer variously to total dietary dry matter, concentrate, forage replacement, protein replacement or formulated TMR. Table 3 reports a common total-diet dry-matter basis only when it can be derived directly from the source; otherwise the original denominator is retained. Ouyang et al. (20, 21) identified poorer intake and gain at high concentrate replacement, whereas Ma et al. (57) maintained growth across 15%−45% silage replacement of peanut-seedling forage and Deng et al. (48) reported favorable responses at 20%−30% silage mulberry in pelleted TMR. Luo et al. (63) reported favorable growth at 20%−30% fermented leaves in goats, while Li et al. (56) used lower leaf-powder levels in pelleted goat diets. Because these denominators are not always biologically equivalent, the review does not define a universal ‘low-to-moderate' inclusion range.

Processing is a plausible effect modifier, but the direct comparative evidence is limited. Long et al. (18) compared pelleted, fresh and fermented TMR containing the same nominal 40% mulberry level; Wang et al. (22) compared ensiled with sun-dried leaves; and Cui et al. (16, 17) compared fermented with dried leaves within one cohort. These studies show processing-associated differences, but the diets were not perfectly matched for all nutrient, physical and fermentation characteristics. Accordingly, processing is discussed as a moderator supported by direct comparisons, not as an isolated causal mechanism.

Animal and analytical context further limit transferability. Direct evidence is geographically concentrated, particularly in Chinese sheep and goat systems, with additional work from Ethiopia, Brazil, Vietnam, Nigeria and other regions. Breeds, sexes, ages, physiological stages and slaughter endpoints vary, and muscle, subcutaneous fat, raw-meat metabolomics and consumer sensory scores are non-equivalent outcomes. The Nigerian study broadened geographic representation but used only 25 West African Dwarf does, randomly assigned to five diets (*n* = 5/treatment), over 90 days; it reported higher digestibility/nitrogen use with increasing mulberry and the highest ADG at the 75% mulberry-forage treatment, but did not assess carcass or meat quality (71). Its small treatment-level sample therefore warrants cautious interpretation. Multi-location trials are needed before assuming transferability across breeds, forage bases, climates and market systems.

### 8.2 Evidence strength, limitations, and research gaps

The evidence base supports a critical synthesis, but certainty differs sharply by outcome. The final matrix contains 67 publication-level records, including 43 direct small-ruminant records at publication level; known companion publications are collapsed when judging independent biological replication. Claim strength is graded using directness, cohort independence, biological replication, intervention comparability and endpoint proximity. Feed value, intake, rumen responses, growth and carcass maintenance have multiple direct experiments, whereas direct muscle amino-acid evidence is less standardized and cooked-meat volatile chemistry, trained sensory validation and storage oxidative stability remain comparatively sparse. Biochemical antioxidant findings are supported by several direct trials, while microbiome and multi-omics results are interpreted more cautiously when based on correlations, enrichment analyses or small terminal subsets.

Several methodological issues lower confidence. First, publication counts overstate replication because Ouyang et al. (20, 21), Cirne et al. (61, 62) and Cui et al. (16, 17) are companion publications from shared experiments; Cao et al. (69) and Khattab et al. (39) are also treated conservatively as companion data sources because their animal population and treatment design closely match. Second, terminal biological n is often much smaller than feeding-phase enrollment: three animals per treatment were used for several outcomes in Ouyang et al. (21), Wang et al. (22) and Chen et al. (60); Cao et al. (59) slaughtered five lambs per diet and used three proteomic replicates; Cui et al. slaughtered six sheep per group with smaller omics subsets. The evidence tables now report treatment-level or terminal replication where available rather than describing trials as “larger” solely from total enrollment. Third, Chinese-language primary studies broaden the evidence base but vary in reporting detail and international indexing, so claims are restricted to outcomes explicitly reported in accessible primary records.

Fourth, mechanistic endpoints need graded interpretation. Microbiome correlations, predicted microbial functions, transcriptomic/proteomic enrichment and metabolomic discrimination are powerful for hypothesis generation, but they do not establish that a specific rumen taxon or pathway causes a meat-quality phenotype. This caution is applied consistently in the abstract, Figure 1 and the evidence tables. Fifth, the geographic concentration of the literature and variation in breed, climate, basal ration and slaughter target constrain generalization. These limitations support a conclusion of context-dependent benefit, not universal improvement.

### 8.3 Future directions

Future work should be prioritized by feasibility and evidential value. First, standardize exposure by reporting cultivar/source, plant part, maturity, processing, storage, total-diet dry-matter inclusion, replacement basis and marker phytochemicals. Second, strengthen basic trial validity through preregistered or prospectively archived protocols, transparent randomization, blinded outcome assessment where feasible, prespecified primary endpoints and power calculations based on terminal biological n. Third, use nutritionally matched controls and replicate key growth, carcass and meat endpoints across breeds and locations before expanding biomarker panels. Fourth, in well-powered studies, integrate feed chemistry, rumen microbiome/metabolome, circulating metabolites and redox markers, muscle/adipose omics, oxidative stability and meat phenotype to test mediation rather than correlation. Fifth, strengthen translation with standardized amino-acid profiling, cooked-meat GC-MS/GC-IMS and sensory validation, together with feed cost, preservation losses, substitution efficiency, carcass value and resource-use outcomes. The priority evidence gaps and design recommendations are summarized in Table 5.

**Table 5:** Priority evidence gaps and recommended study designs.

Evidence problem	Why it matters	Recommended response
Non-equivalent dose reporting	Percent of concentrate, total DM and ingredient replacement are conflated	Report total-DM inclusion, absolute intake and nutrient/ingredient replacement equivalence
Variable feed chemistry	Cultivar, maturity, leaf:stem ratio and processing differ	Characterize nutrients plus marker polyphenols/flavonoids for each batch
Small terminal samples	Meat/omics conclusions often rely on *n* = 3-6/group	Power terminal endpoints and report animal vs. pen vs. analytical replicate
Companion-cohort double counting	One experiment can generate multiple publications	Identify shared cohorts explicitly in reviews and meta-analyses
Incomplete rumen-to-meat causality	Rumen, blood and meat are often measured in separate studies	Integrate feed, rumen, systemic and muscle measurements in the same animals
Weak AA/flavor validation	Few quantitative AA datasets; metabolomics rarely linked to eating quality	Standardize AA analysis and combine cooked-volatiles with trained/consumer sensory tests
Limited external validity	Evidence is concentrated in specific breeds and regions	Conduct multi-breed, multi-location validation under different basal feed systems
Limited production economics	Biological responses are rarely converted to system value	Report feed cost, preservation loss, substitution efficiency and carcass value
Trial transparency and bias control	Randomization, blinding and prespecified outcomes are inconsistently reported	Prospectively archive/preregister protocols; report randomization and allocation; blind laboratory/meat-quality assessment where feasible; prespecify primary endpoints

AA, amino acids; DM, dry matter; GC-IMS, gas chromatography-ion mobility spectrometry; GC-MS, gas chromatography-mass spectrometry.

## 9 Conclusion

Mulberry leaves are a credible alternative feed resource for sheep and goats, but the evidence does not support treating them as a uniformly beneficial additive. Their practical value comes from conventional nutrients combined with potentially functional phytochemicals, and the response depends on feed form, dose denominator, the ingredient replaced and the nutritional balance of the complete ration. Within study-specific tolerated ranges or nutritionally equivalent replacement strategies, intake, growth and carcass performance are commonly maintained; excessive substitution of nutrient-dense concentrate can reduce intake and gain. Because studies use incompatible dose denominators, a generally applicable “low-to-moderate” inclusion range is not proposed.

Meat-quality effects are promising but heterogeneous. Several independent sheep and goat studies report changes in tenderness, water retention, muscle fatty acids and amino-acid composition, but other studies show neutral sensory and physicochemical responses or adverse water loss. Evidence from subcutaneous adipose tissue is not treated as equivalent to edible-muscle fatty-acid composition, and flavor-related metabolites are not described as sensory improvement without sensory or aroma-activity validation. Microbiome and multi-omics findings provide mechanistic support but remain associative where causal mediation was not tested. The evidence therefore supports responsiveness of muscle composition and selected quality traits rather than universal improvement.

The most useful next step is therefore not to search for a universal optimal inclusion percentage, but to standardize exposure and comparator diets, increase independent terminal replication, test breed and production-system transferability, and connect mechanistic measurements with meat and economic outcomes. Mulberry therefore has potential as a locally useful component of small-ruminant feeding systems, but claims of regional adaptability or commercial relevance require direct multi-location and economic evidence.
