# Supplementary material for: Mulberry (Morus alba) leaves in sheep and goat nutrition: a critical narrative review of rumen function, performance, meat quality, and mechanistic evidence
Source: Front Vet Sci. 2026 Oct 12;13:1986200. doi: 10.3389/fvets.2026.1986200 (PMC13699504; doi:10.3389/fvets.2026.1986200)
Supplement: Supplementary file 1 [file Table_1.docx]

***Supplementary Material***

# 1 **Supplementary Data**

Sixty-seven publication-level records are mapped in the supplied evidence set. Direct sheep/goat evidence is distinguished from supporting ruminant, feed-level, and review evidence. Companion publications from shared animal cohorts are not treated as independent experiments.

*Appraisal dimensions used in the manuscript: species directness, cohort independence, biological replication, intervention comparability, endpoint proximity to meat quality, and causal strength of mechanistic evidence.*

**Supplementary Table S1. Study-level evidence extraction and appraisal matrix (67 publication-level records), presented in three linked parts for readability.**

**Supplementary Table S1A. Study identification and evidence classification.**

| **ID** | **First author** | **Year** | **Article title** | **Article type** | **Evidence relevance** | **Evidence directness** |
| --- | --- | --- | --- | --- | --- | --- |
| 1 | Xi Huang | 2019 | Lipid oxidation and its implications to meat quality and human health | Narrative review | Moderate - supporting meat-quality mechanism evidence | Review / synthesis |
| 2 | Gebrekidan Tesfay | 2018 | Substitution of Mulberry Leaf Meal on Feed Intake, Body Weight and Carcass Characteristics of Tigray Highland Lambs | Primary feeding trial | High - direct small-ruminant mulberry evidence | Direct small-ruminant |
| 3 | Gebrekidan Tesfay | 2018 | Feeding value potential of mulberry (Morus alba) leaf meal to replace concentrate mix | Feed evaluation - in vitro/in situ | Moderate to high - direct feed-value evidence | Feed-level / in vitro |
| 4 | Yafeng Huang | 2023 | Feeding regimens affecting carcass and quality attributes of sheep and goat meat - A comprehensive review | Comprehensive narrative review | High supporting - small-ruminant meat-quality framework | Review / synthesis |
| 5 | Yang Luo | 2025 | Supplementation with mulberry leaves improves growth performance and meat quality of Xiangdong black goats | Primary goat feeding trial | Very high - direct goat, dose-response, meat-composition evidence | Direct small-ruminant |
| 6 | Dwi Yulistiani | 2015 | Effects of Supplementation of Mulberry (Morus alba) Foliage and Urea-rice Bran as Fermentable Energy and Protein Sources in Sheep Fed Urea-treated Rice Straw Based Diet | Primary sheep digestibility/rumen trial | High - direct small-ruminant nutrition evidence | Direct small-ruminant |
| 7 | Jialiang Ouyang | 2019 | Effects of Dietary Mulberry Leaf Powder in Concentrate on the Rumen Fermentation and Ruminal Epithelium in Fattening Hu Sheep | Primary sheep feeding/rumen trial | Very high - direct dose-response rumen mechanism evidence | Direct small-ruminant |
| 8 | Hua Sun | 2020 | The Effect of Replacing Wildrye Hay with Mulberry Leaves on the Growth Performance, Blood Metabolites, and Carcass Characteristics of Sheep | Primary sheep feeding/meat trial | Very high - direct sheep growth, carcass, meat-fat evidence | Direct small-ruminant |
| 9 | Faiz-ul Hassan | 2020 | Potential of Mulberry Leaf Biomass and Its Flavonoids to Improve Production and Health in Ruminants: Mechanistic Insights and Prospects | Narrative mechanistic review | Very high supporting - mulberry/ruminant foundation review | Review / synthesis |
| 10 | Haoqi Han | 2022 | Replacement of Maize Silage and Soyabean Meal with Mulberry Silage in the Diet of Hu Lambs on Growth, Gastrointestinal Tissue Morphology, Rumen Fermentation Parameters and Microbial Diversity | Primary sheep feeding/rumen-microbiome trial | Very high - direct rumen/growth evidence | Direct small-ruminant |
| 11 | Mingyan Wang | 2022 | Effect of the Replacement of Maize Silage and Soyabean Meal with Mulberry Silage in the Diet of Hu Lambs on Growth Performance, Serum Biochemical Indices, Slaughter Performance, and Meat Quality | Primary sheep feeding/carcass/meat trial | Very high - direct meat and carcass evidence | Direct small-ruminant |
| 12 | Lixue Wang | 2022 | Protective Application of Morus and Its Extracts in Animal Production | Narrative review | High supporting - broad Morus bioactivity/animal-production evidence | Review / synthesis |
| 13 | Wenjie Wang | 2024 | Effects of Feeding Systems on the Growth Performance, Carcass Characteristics, and Meat Quality in Sheep: A Meta-Analysis | Meta-analysis | High supporting - quantitative sheep feeding-system evidence | Review / synthesis |
| 14 | Mostafa S. A. Khattab | 2026 | Mulberry Silage as Alternative to Soybean Meal Protein in Ruminant Diet: Effect on Growth Performance, Digestion, Antioxidant Capacity, and Carcass Characteristics of Goats | Primary goat feeding/carcass trial | Very high - recent direct goat evidence | Direct small-ruminant |
| 15 | Liangyong Guo | 2025 | Effects of dietary addition of mulberry leaf powder on blood metabolites and fecal microbiota composition in Hu sheep | Primary sheep physiology/microbiota trial | High - direct sheep physiological evidence; no meat outcomes | Direct small-ruminant |
| 16 | Jinzhuan Li | 2024 | The Fermentation Quality, Antioxidant Activity, and Bacterial Community of Mulberry Leaf Silage with Pediococcus, Bacillus, and Wheat Bran | Silage fermentation study | Moderate to high - feed-processing evidence | Feed-level / in vitro |
| 17 | Zekeriya Safa İnanç | 2025 | In Vitro and In Situ Evaluation of White Mulberry (Morus alba) Pomace and Leaf: Fermentation Kinetics, Digestibility, and Potential as Alternative Ruminant Feed Sources | In-vitro/in-situ ruminant feed study | Moderate - current feed-value/fermentation evidence | Feed-level / in vitro |
| 18 | Yong Long | 2022 | Effect of Mulberry Leaf TMR on Growth Performance, Meat Quality and Expression of Meat Quality Master Genes (ADSL, H-FABP) in Crossbred Black Goats | Primary goat feeding/meat/molecular trial | Very high - direct goat meat-quality evidence | Direct small-ruminant |
| 19 | Mariero Gawat | 2023 | Goat Meat: Production and Quality Attributes | Narrative review | High supporting - goat meat-quality reference | Review / synthesis |
| 20 | Rong Wan | 2026 | Effects of fermented mulberry leaves on growth performance, nutrient digestibility, diarrhea, and intestinal microecology in weaned piglets: a preliminary study | Primary non-ruminant feeding trial | Low to moderate - indirect fermented-mulberry mechanism evidence | Indirect non-ruminant |
| 21 | Tiantian Ke | 2023 | Review of Feeding Systems Affecting Production, Carcass Attributes, and Meat Quality of Ovine and Caprine Species | Narrative review | High supporting - small-ruminant feeding/meat synthesis | Review / synthesis |
| 22 | Tianyu Su | 2025 | Effects of intramuscular fat on the flavor of fresh sheep and goat meat: Recent insights into pre-mortem and post-mortem factors | Narrative flavor review | High supporting - sheep/goat flavor mechanism evidence | Review / synthesis |
| 23 | Yingying Liu | 2021 | Mulberry leaf powder regulates antioxidative capacity and lipid metabolism in finishing pigs | Primary non-ruminant feeding/meat-metabolism trial | Moderate - indirect antioxidant/lipid mechanism evidence | Indirect non-ruminant |
| 24 | Mengwei Li | 2022 | Mulberry flavonoids modulate rumen bacteria to alter fermentation kinetics in water buffalo | Primary ruminant flavonoid/rumen-microbiome trial | High supporting - direct ruminant mechanism evidence | Supporting ruminant |
| 25 | Bing Wang | 2022 | Assessment of components related to flavor and taste in Tan-lamb meat under different silage-feeding regimens using integrative metabolomics | Primary feeding trial + integrative metabolomics | High - direct small-ruminant flavor/metabolomics evidence | Direct small-ruminant |
| 26 | Bing Wang | 2021 | Effects of mulberry leaf silage on antioxidant and immunomodulatory activity and rumen bacterial community of lambs | Primary feeding trial + rumen microbiome | High - direct rumen microbiota and antioxidant evidence | Direct small-ruminant |
| 27 | Jialiang Ouyang | 2020 | Effects of dietary mulberry leaf powder on growth performance, blood metabolites, meat quality, and antioxidant enzyme-related gene expression of fattening Hu lambs | Primary dose-response feeding trial | High - direct growth, meat-quality and antioxidant-gene evidence | Direct small-ruminant |
| 28 | Luís Gabriel Alves Cirne | 2019 | Nutritional characteristics of meat from lambs fed diets containing mulberry hay | Primary feeding trial - companion fatty-acid publication | High - direct muscle fatty-acid and nutritional-quality evidence | Direct small-ruminant |
| 29 | Luís Gabriel Alves Cirne | 2018 | Physicochemical and sensory characteristics of meat from lambs fed diets containing mulberry hay | Primary feeding trial - companion meat-quality/sensory publication | High - direct physicochemical and consumer sensory evidence | Direct small-ruminant |
| 30 | Qirui Hou | 2025 | Synergistic effect of corn straw and mulberry leaves on in vitro fermentation, growth performance and antioxidant capacity of sheep | Combined in vitro fermentation + primary sheep feeding trial | High - direct feed synergy, meat water-holding and antioxidant evidence | Direct small-ruminant + in vitro |
| 31 | Yang Cao | 2024 | The Effect of Mulberry Silage Supplementation on the Carcass Fatness and Long-Chain Fatty Acid Composition of Growing Lambs Compared with Traditional Corn Silage | Primary feeding trial + proteomics | High - direct carcass fatness, adipose fatty-acid and lipid-metabolism evidence | Direct small-ruminant |
| 32 | Yaoyue Wang | 2020 | Comparison of Rumen Microbiota and Serum Biochemical Indices in White Cashmere Goats Fed Ensiled or Sun-Dried Mulberry Leaves | Primary goat feeding + rumen microbiome trial | Very high - direct White Cashmere goat rumen/feed-form evidence | Direct small-ruminant |
| 33 | Ling Chen | 2023 | Effects of Dietary Supplementation of Selenium-rich Mulberry Leaf Powder on Slaughtering Traits and Muscular Fatty Acids in Shannan White Goats | Primary goat feeding/meat trial | High - direct White-goat carcass, meat-quality and muscle fatty-acid evidence | Direct small-ruminant |
| 34 | Xiaopeng Cui | 2023 | Transcriptomics and metabolomics analysis reveal the anti-oxidation and immune boosting effects of mulberry leaves in growing mutton sheep | Primary feeding trial + transcriptomics/metabolomics | High - direct growth, antioxidant and multi-omics mechanism evidence | Direct small-ruminant |
| 35 | Xiaopeng Cui | 2024 | Mulberry leaves supplementation alters lipid metabolism and promotes fatty acid β oxidation in growing mutton sheep | Companion primary paper + lipidomics/transcriptomics | High - direct tissue lipid-metabolism mechanism evidence | Direct small-ruminant - companion cohort |
| 36 | Hanan A. M. Hassanien | 2026 | Dietary inclusion of Morus alba leaves influences milk production and offspring growth performance in dairy goats | Primary dairy-goat feeding + separate digestibility/rumen trial | Moderate-high - direct goat feed/rumen evidence; supporting meat-quality context | Direct small-ruminant - supporting meat-quality |
| 37 | S. S. Omar | 1999 | Voluntary intake and digestibility of mulberry (Morus alba) diets by growing goats | Primary goat feeding trial | High - foundational direct goat intake/digestibility evidence | Direct small-ruminant |
| 38 | Nguyen Xuan Ba | 2005 | Ensiling of mulberry foliage (Morus alba) and the nutritive value of mulberry foliage silage for goats in central Vietnam | Primary goat silage/digestibility trial | High - direct goat feed-preservation and nutrient-use evidence | Direct small-ruminant |
| 39 | K. Kandylis | 2009 | The nutritive value of mulberry leaves (Morus alba) as a feed supplement for sheep | Primary sheep feed-value/digestibility study | High - direct sheep feed-value evidence | Direct small-ruminant |
| 40 | J. Salinas-Chavira | 2011 | Effect of increasing levels of white mulberry leaves (Morus alba) on ruminal dry matter degradability in lambs | Primary lamb rumen degradability trial | Moderate-high - direct rumen degradability evidence | Direct small-ruminant |
| 41 | J. X. Liu | 2001 | Effects of mulberry leaves to replace rapeseed meal on performance of sheep feeding on ammoniated rice straw diet | Primary sheep feeding trial | High - foundational direct sheep replacement/performance evidence | Direct small-ruminant |
| 42 | Dandan Chen | 2015 | Effects of mulberry leaf flavonoid and resveratrol on methane emission and nutrient digestion in sheep | Primary sheep phytochemical metabolism trial | High - direct sheep flavonoid/methane/digestibility mechanism evidence | Direct small-ruminant - purified extract |
| 43 | Dwi Yulistiani | 2024 | Increasing the nutritive value of a rice straw-based diet using mulberry and Leucaena to promote the growth performance of lambs | Primary lamb feeding/digestibility trial | Very high - recent direct lamb nutrition evidence | Direct small-ruminant |
| 44 | Qiwen Fan | 2026 | Effects of fermented mulberry leaves on rumen fermentation and microbiota in rumen fluid of heat-stressed Hu sheep | Primary sheep feeding + rumen microbiome trial | Very high - recent direct sheep fermented-mulberry/rumen evidence | Direct small-ruminant |
| 45 | Cuong Chi Vu | 2011 | The nutritive value of mulberry leaves (Morus alba) and partial replacement of cotton seed in rations on the performance of growing Vietnamese cattle | Ruminant digestibility + cattle feeding trial | High - supporting cross-ruminant feed-value/performance evidence | Supporting ruminant |
| 46 | N. T. Huyen | 2012 | Effect of Mulberry leaf pellet (MUP) supplementation on rumen fermentation and nutrient digestibility in beef cattle fed on rice straw-based diets | Cattle rumen/digestibility Latin-square trial | High - supporting rumen/digestibility evidence | Supporting ruminant |
| 47 | N. D. Tan | 2012 | Enhancing mulberry leaf meal with urea by pelleting to improve rumen fermentation in cattle | Cattle rumen/microbial Latin-square trial | High - supporting processing/rumen evidence | Supporting ruminant |
| 48 | Ehab Bo Trabi | 2017 | Effect of glucose and lactic acid bacteria on the fermentation quality, chemical compositions and in vitro digestibility of mulberry (Morus alba) leaf silage | Silage fermentation / in-vitro study | Moderate-high - feed-processing evidence | Feed-level / in vitro |
| 49 | Z. Dong | 2020 | Effects of additives on the fermentation quality, in vitro digestibility and aerobic stability of mulberry (Morus alba L.) leaves silage | Silage fermentation / in-vitro study | Moderate-high - feed-processing/aerobic stability evidence | Feed-level / in vitro |
| 50 | Yan Li | 2022 | Effects of mulberry branch and leaves silage on microbial community, rumen fermentation characteristics, and milk yield in lactating dairy cows | Dairy-cow feeding + rumen microbiome trial | High - supporting rumen/microbiome evidence | Supporting ruminant |
| 51 | Patipan Hnokaew | 2024 | Influence of dietary mulberry (Morus alba L.) leaf supplementation on production performance, blood metabolites, rumen fermentation characteristics and ruminal bacteria community in lactating dairy cows | Dairy-cow feeding + rumen microbiome trial | High - supporting rumen/microbiome and metabolic tolerance evidence | Supporting ruminant |
| 52 | A. A. K. Saleh | 2026 | Effect of dietary incorporation of mulberry leaves on nutritive value of rations, digestibility, growth performance and blood constituents of male Ossimi lambs | Primary sheep feeding/digestibility trial | Very high - direct recent sheep dose-response evidence | Direct small-ruminant |
| 53 | M. P. Doran | 2007 | Total tract and rumen digestibility of mulberry foliage (Morus alba), alfalfa hay and oat hay in sheep | Primary sheep digestibility trial | Very high - foundational direct sheep digestibility evidence | Direct small-ruminant |
| 54 | Bing Geng | 2024 | Effects of dietary mulberry leaves on growth, production performance, gut microbiota, and immunological parameters in poultry and livestock: a systematic review and meta-analysis | Systematic review and meta-analysis | High - contextual synthesis; cross-species rather than small-ruminant-specific | Review / synthesis |
| 55 | Gulab Khan Rohela | 2025 | Mulberry (Morus spp.): A promising field crop for livestock forage | Narrative forage/livestock review | High - current contextual review of mulberry forage systems | Review / synthesis |
| 56 | D. Saddul | 2005 | Evaluation of mulberry (Morus alba) as potential feed supplement for ruminants: Effect of plant maturity on in situ disappearance and in vitro intestinal digestibility of plant fractions | Feed evaluation / in situ + in vitro study | High - foundational evidence for maturity-dependent feed value | Feed-level / direct ruminant degradation |
| 57 | A. Sahoo | 2011 | Evaluation of feeding value of Eupatorium adenophorum in combination with mulberry leaves | Primary sheep feeding + in vitro fermentation trial | Moderate-high - direct sheep feeding evidence with a mixed-forage co-intervention | Direct small-ruminant + in vitro |
| 58 | T. Ma | 2017 | Dietary supplementation with mulberry leaf flavonoids inhibits methanogenesis in sheep | Primary sheep phytochemical + rumen trial | Very high - direct mechanistic sheep evidence | Direct small-ruminant - purified extract |
| 59 | Y. Kou | 2021 | Effects of diets with different proportions of whole mulberry on growth performance, nutrient apparent digestibility, serum antioxidant indexes and rumen fermentation parameters of fattening Hu sheep | Primary sheep feeding/digestibility trial | High - direct sheep dose evidence | Direct small-ruminant |
| 60 | Y. Luo | 2022 | Effects of different adding proportions of fermented mulberry leaves on growth performance, serum indexes, muscle amino acid and fatty acid contents of Xiangdong black goats | Primary goat feeding + meat-composition trial | Very high - direct goat amino-acid and fatty-acid evidence | Direct small-ruminant |
| 61 | H. Ma | 2022 | Effects of silage mulberry leaves on production performance and meat quality of Hu sheep | Primary sheep feeding + meat-quality trial | Very high - large direct meat-quality and muscle-composition evidence | Direct small-ruminant |
| 62 | X. Li | 2023 | Effects of adding mulberry leaf powder into TMR granulated diets on growth performance, slaughter performance and economic benefits of Liuyang black goats | Primary goat feeding/carcass/economic trial | High - direct goat production and economic evidence | Direct small-ruminant |
| 63 | Y. Wang | 2023 | Effects of replacing whole corn silage with Morus alba silage on growth performance and nutrient digestion and metabolism of fattening Hu sheep | Primary sheep feeding/digestion trial | High - direct forage-replacement evidence | Direct small-ruminant |
| 64 | P. Cao | 2025 | Effects of substituting mulberry silage for soybean meal on blood indexes, rumen fermentation, and rumen bacteria of goats | Primary goat feeding + rumen microbiome trial | High mechanistic relevance - companion to protein-replacement goat evidence | Direct small-ruminant - companion cohort |
| 65 | W. Deng | 2025 | Effects of incorporating silage mulberry leaves into TMR pellet diets on production performance, meat quality and rumen fermentation of Hu sheep | Primary sheep feeding + meat-quality/rumen trial | Very high - large recent integrated sheep evidence | Direct small-ruminant |
| 66 | X. Han | 2023 | Effects of silage forage mulberry on growth performance, slaughter performance and amino acid and fatty acid contents in longissimus dorsi of Hu sheep | Primary sheep feeding + muscle-composition trial | Very high - direct sheep amino-acid/fatty-acid evidence | Direct small-ruminant |
| 67 | O.A. Adebayo | 2026 | Digestibility, nitrogen balance and growth performance of West African Dwarf (WAD) does fed mulberry (Morus alba) with Guinea grass (Panicum maximum) foliage and cassava peel-based concentrate | Primary goat feeding + digestibility/growth trial | High - direct goat intake, digestibility, nitrogen-balance and growth evidence | Direct small-ruminant |

**Supplementary Table S1B. Animal/model information, mulberry intervention, measured outcomes, and source.**

| **ID** | **Animals / model & sample** | **Mulberry form + inclusion / duration** | **Key measured outcomes** | **Source / DOI** |
| --- | --- | --- | --- | --- |
| 1 | Not applicable. This is a literature review and did not use a single experimental animal population. It discusses meat and biological oxidation across species rather than focusing on goats or sheep. | No mulberry treatment was used. The paper is relevant only for explaining how antioxidant-rich feeds such as mulberry might influence meat quality through oxidative pathways. Not applicable because this was not a feeding trial. | Intake/digestibility: Feed intake and digestibility were not evaluated. Rumen/metabolism: Rumen fermentation and microbiota were not evaluated. At the metabolic level, the review explains oxidative stress as an imbalance favoring oxidants and discusses enzymatic and non-enzymatic antioxidant defenses. Growth: Growth performance and feed efficiency were not evaluated. Carcass: Slaughter and carcass traits were not evaluated. Meat quality: The review identifies lipid oxidation as a major cause of deterioration in meat flavor, color, texture, nutritional value, water-holding capacity, and emulsifying/functional properties. It therefore provides a mechanistic basis for interpreting treatment-related changes in oxidative stability and sensory quality. FA/AA/flavor: The paper explains that unsaturated fatty acids, especially highly unsaturated lipids, are vulnerable to oxidation and can generate secondary volatile compounds that influence odor and flavor. It does not provide goat-specific amino-acid or sensory intervention data. Antioxidant/physiology: The review discusses endogenous antioxidant enzymes and antioxidant compounds, sources of reactive species, and the value of additional dietary antioxidants in maintaining redox balance. These are general mechanisms, not evidence that mulberry specifically causes such effects in small ruminants. | https://doi.org/10.1007/s10068-019-00631-7 |
| 2 | Thirty intact yearling male Tigray Highland lambs with an average initial body weight of about 17.8 +/- 0.95 kg were used. The animals were blocked by live weight, giving approximately six animals per dietary treatment. | Shade-dried mulberry foliage processed as mulberry leaf meal was used as the protein-rich supplement/replacement ingredient. Mulberry replaced the concentrate mixture progressively from 0% to 100% on an iso-nitrogenous basis. Lambs were adapted for 15 days, followed by a 90-day feeding period; a subsequent digestibility collection was conducted before slaughter evaluation. | Intake/digestibility: Complete replacement with mulberry leaf meal increased total dry-matter, organic-matter, NDF, and ADF intake compared with concentrate alone. Apparent nutrient digestibility was generally maintained and some fiber-digestibility responses favored diets containing more mulberry, supporting good ruminal utilization of the leaf meal. Rumen/metabolism: The study did not directly profile rumen microbiota or fermentation metabolites. The intake and digestibility results nevertheless indicate that mulberry leaf meal was effectively utilized as a ruminant feed and did not cause a broad digestive penalty across the tested replacement range. Growth: Average growth responses were broadly comparable among treatments; the study did not show a consistent depression in body-weight gain when concentrate was replaced by mulberry leaf meal. Carcass: Slaughter weight and empty body weight were higher in lambs receiving sole mulberry leaf meal than in the concentrate-only treatment. Hot carcass weight and dressing percentage on an empty-body-weight basis did not differ consistently across treatments, although some treatment differences in carcass indices were reported. Meat quality: Detailed physicochemical meat-quality traits such as pH, color, water-holding capacity, shear force, or intramuscular fat were not measured. The paper contributes carcass evidence rather than comprehensive meat-quality evidence. FA/AA/flavor: Fatty acids, amino acids, volatile compounds, and sensory attributes were not analyzed. Antioxidant/physiology: Blood antioxidant or metabolic biomarkers were not a major component of this study. | https://doi.org/10.14334/jitv.v23i1.1634 |
| 3 | There was no production feeding trial in goats or sheep. In-sacco degradation was measured using three rumen-fistulated Boran-Holstein Friesian crossbred steers as incubation hosts. | Dried mulberry (Morus alba) leaf meal was evaluated. The same graded replacement concept as the related lamb work was used, from concentrate alone to mulberry leaf meal alone. Because this was a laboratory/in-sacco feed evaluation, there was no long-term animal feeding duration. | Intake/digestibility: Mulberry leaf meal compared favorably with concentrate for major nutrients, had substantially more ash and calcium, and showed high degradability. All treatment diets exceeded about 66% dry-matter degradability by 24 h. The mulberry-only diet had a large slowly degradable fraction but somewhat lower soluble fraction/effective degradability than diets with lower mulberry proportions. Rumen/metabolism: The paper evaluated ruminal degradation kinetics rather than complete fermentation or microbiome profiles. The high degradability supports the premise that mulberry leaf nutrients are accessible to rumen microorganisms. Growth: Growth performance and feed efficiency were not measured in this paper. Carcass: No slaughter or carcass outcomes were measured. Meat quality: No meat-quality traits were measured. FA/AA/flavor: No muscle fatty-acid, amino-acid, volatile, or sensory outcomes were measured. Antioxidant/physiology: No blood or tissue antioxidant endpoints were measured. | https://doi.org/10.5897/IJLP2018.0479 |
| 4 | Not applicable as a single cohort. The review synthesizes published sheep and goat studies using multiple breeds, ages, sexes, production systems, and durations. | Mulberry was not the central intervention. The paper deals with feeding systems broadly and is therefore background/comparator evidence for understanding how diet and production system influence meat quality. No single mulberry dose, basal diet, or duration applies because many studies were reviewed. | Intake/digestibility: The review generally shows that concentrate supplementation or indoor feeding improves growth rate and often carcass yield compared with pasture-only systems, while forage/pasture systems can modify nutrient intake and meat composition. Digestibility itself is not the main focus. Rumen/metabolism: Rumen fermentation and microbiota are not deeply synthesized; the emphasis is downstream carcass and meat outcomes. Growth: Stall-fed or concentrate-supplemented lambs/kids generally had higher average daily gain than pasture-only animals, although good-quality pasture and supplemented grazing could narrow or eliminate this difference. Carcass: Indoor feeding and supplemented grazing generally increased carcass weight/yield relative to pasture-only feeding. The review emphasizes that these responses are affected by species, breed, diet quality, and finishing system. Meat quality: Feeding system influenced meat color, tenderness, juiciness, intramuscular fat, protein, and flavor, but responses differed between sheep and goats. Pasture systems frequently produced leaner meat, whereas concentrate feeding often increased intramuscular fat and some eating-quality traits. FA/AA/flavor: Pasture-fed meat generally had a more favorable fatty-acid profile, including more n-3 PUFA and lower n-6/n-3 ratios, while concentrate feeding tended toward less favorable fatty-acid indices. Flavor intensity also differed by feeding system, with species-specific responses. Amino-acid data were less central. Antioxidant/physiology: The paper does not provide a unified antioxidant biomarker analysis, although diet-induced differences in meat composition and quality are discussed. | https://doi.org/10.5713/ab.23.0051 |
| 5 | Twenty-five healthy male Xiangdong black goats, 3-4 months old, with average initial body weight 13.17 +/- 1.67 kg were used; five goats were assigned to each treatment. | Air-dried Morus alba leaves were ground into powder and incorporated into pelleted diets. Inclusion levels were 0, 5, 10, 15, and 20% on an air-dry basis, replacing portions of soybean meal/rice straw and other ingredients while maintaining similar CP and ME. Goats adapted for 10 days and the feeding experiment lasted 92 days. | Intake/digestibility: Initial and final dry-matter intake increased with increasing mulberry level, and the growth response suggests good acceptance of the diets. The paper focused more on intake/growth than a full digestibility balance. Rumen/metabolism: Rumen fermentation and microbiota were not directly measured. The study instead evaluated serum physiology and muscle composition. Growth: Final body weight and average daily gain increased linearly/quadratically with increasing mulberry inclusion; the 20% group showed the highest ADG. This provides direct dose-response evidence that moderate-to-high inclusion can support growth in this breed. Carcass: Animals were slaughtered for muscle collection, but detailed carcass yield/dressing traits were not the principal outcomes. Therefore, the study is stronger for growth and muscle composition than for carcass evaluation. Meat quality: The paper evaluated muscle composition rather than a full panel of pH, color, water-holding capacity, or shear force. Its claim of improved meat quality is mainly supported by changes in fatty-acid/amino-acid characteristics and antioxidant-related status. FA/AA/flavor: Saturated fatty acids decreased linearly with increasing mulberry; C18:0 was significantly lower at 20%. Amino-acid profiles were measured, and the authors interpreted the high-inclusion diet as favorable for meat flavor/nutritional quality. Volatile compounds and trained sensory evaluation were not measured, so 'flavor improvement' is an inference from compositional precursors rather than direct sensory proof. Antioxidant/physiology: Total antioxidant capacity increased with mulberry inclusion. The 15% treatment significantly increased T-AOC, SOD, catalase, and IgG relative to control, supporting enhanced antioxidant and immunomodulatory status. The paper reported 15% as particularly favorable for these physiological responses. | https://doi.org/10.5713/ab.24.0427 |
| 6 | Twelve sheep were used. The paper focused on digestibility and rumen responses; four animals were allocated to each of three dietary treatments. | Mulberry (Morus alba) foliage was used as a dried/processed forage supplement. Mulberry supplied the supplement in T1 and half the supplement in T2; the recommended level corresponded to about 1.2% BW or roughly 32% of the total diet. The digestibility trial included 14 days of adaptation, 7 days of sample collection, and 1 day of rumen sampling. | Intake/digestibility: Dry-matter intake and DM, OM, and CP digestibility did not differ significantly among diets. Fiber digestibility was higher in the mulberry-containing diets than in the rice-bran/urea-only treatment, and all groups maintained positive nitrogen balance. Rumen/metabolism: Rumen ammonia was higher in T2/T3 than T1, but concentrations in all treatments were adequate for microbial growth. Total VFA concentration was highest with the mulberry treatment, while propionate proportion was highest in the rice-bran/urea treatment. Microbial nitrogen supply was comparable between T1 and T3 and lower in T2. Growth: This was a digestibility study, not a long-term growth trial, so growth performance and feed conversion were not robustly evaluated. Carcass: No slaughter or carcass measurements were reported. Meat quality: No physicochemical meat-quality traits were measured. FA/AA/flavor: No muscle fatty acids, amino acids, volatiles, or sensory traits were analyzed. Antioxidant/physiology: Blood antioxidant and systemic physiological markers were not central outcomes. | https://doi.org/10.5713/ajas.14.0406 |
| 7 | Forty three-month-old male Hu sheep with initial body weight 16.5 +/- 0.6 kg were used, with eight animals per treatment. | Mulberry leaves were processed as mulberry leaf powder and incorporated into the concentrate. The concentrate contained 0-60% MLP. The total study covered approximately 12 weeks (2-week adaptation + 10-week experiment), with repeated body-weight, fecal, and rumen sampling. | Intake/digestibility: At 15% and 30% MLP, DMI and ADG were not different from control; at 45-60%, both were reduced. Apparent OM and NDF digestibility increased with MLP inclusion, indicating improved fiber/nutrient utilization despite poorer intake at very high inclusion. Rumen/metabolism: Rumen ammonia tended to decrease as MLP increased, while microbial protein increased. The study also found changes consistent with greater acetate production and improved ruminal epithelial development: papilla width/structure and stratum basale characteristics were enhanced, supporting absorptive/metabolic adaptation. Growth: Growth was maintained at 15-30% MLP but depressed at 45-60%, showing a clear non-linear dose response and supporting an intermediate optimum rather than unrestricted inclusion. Carcass: No slaughter or carcass outcomes were measured. Meat quality: No meat pH, color, tenderness, water-holding capacity, or proximate meat-quality outcomes were measured. FA/AA/flavor: No muscle fatty-acid, amino-acid, volatile, or sensory analyses were performed. Antioxidant/physiology: Systemic antioxidant biomarkers were not the main focus; physiological evidence came mainly from rumen fermentation and epithelial morphology. | https://doi.org/10.3390/ani9050218 |
| 8 | Thirty-two four-month-old male Small-tailed Han sheep with initial body weight 25.15 +/- 1.03 kg were used, eight per treatment. | Mulberry leaves were used as hay/dried forage replacing conventional Chinese wildrye hay. Mulberry inclusion was 0, 8, 24, or 32% of the ration on an air-dry basis as a substitute for wildrye. The feedlot study lasted 65 days, including 10 days adaptation and 55 days formal feeding. | Intake/digestibility: ADG and feed conversion were unchanged. DM, OM and ADF digestibility were higher with mulberry, and the 24% treatment had higher CP and ether-extract digestibility, indicating improved nutrient use at an intermediate level. Rumen/metabolism: Rumen pH and total VFA were not significantly changed, but microbial protein was higher in ML24/ML32 and acetate/butyrate proportions differed among treatments. These data support altered rumen nutrient use without evidence of acidosis. Growth: Final body weight, ADG, and FCR did not differ significantly, showing that mulberry could replace wildrye without compromising growth under these conditions. Carcass: Carcass weight, dressing percentage, and longissimus muscle area were not significantly different. Thus, the replacement maintained carcass performance rather than producing a large carcass advantage. Meat quality: Intramuscular fat was higher in the 24% group, and 24-h longissimus pH was also higher than control. These are direct meat-related changes; the paper did not demonstrate broad improvements in all conventional quality parameters. FA/AA/flavor: The 24% treatment reduced saturated fatty acids and increased monounsaturated fatty acids relative to control. This supports a diet-induced shift in meat lipid composition. Amino acids, detailed volatile profiles, and sensory panels were not reported. Antioxidant/physiology: Most serum variables were unchanged except blood urea nitrogen, suggesting no major adverse systemic metabolic effect across treatments. The study did not provide an extensive antioxidant biomarker panel. | https://doi.org/10.3390/ani10112018 |
| 9 | Not applicable as a single experiment. Evidence was drawn from cattle, buffalo, sheep, goats and other relevant experimental models. | The review covers fresh/dried mulberry leaf biomass and mulberry leaf flavonoids/extracts, with emphasis on Morus species used in livestock feeding. No single inclusion level or duration applies. The review reports wide variation in dietary use depending on species, feed form, and production objective. | Intake/digestibility: The authors summarize evidence that mulberry leaves are highly palatable, protein-rich, and digestible, and that dietary inclusion can enhance fiber digestion and nutrient utilization in ruminants. These conclusions are based on multiple primary studies rather than a pooled quantitative estimate. Rumen/metabolism: A major focus is rumen function. The review proposes that flavonoids can alter microbial populations, stimulate some cellulolytic bacteria, increase total VFA/propionate in some conditions, and suppress methanogens/protozoa, potentially reducing methane production. Growth: The review reports generally favorable effects on production, including milk and performance responses, but emphasizes that outcomes depend on dose, feed form, basal diet, and animal status. Carcass: Carcass and meat outcomes are not the dominant focus, so this paper should not replace direct carcass/meat trials in our evidence hierarchy. Meat quality: The paper offers limited direct synthesis of pH, tenderness, color, or water-holding capacity. Its value for meat quality is mainly mechanistic and nutritional rather than direct. FA/AA/flavor: Detailed goat/sheep meat fatty-acid, amino-acid, volatile and sensory evidence is not its central contribution. It is more useful for explaining bioactive and rumen-mediated pathways. Antioxidant/physiology: The review emphasizes antioxidant properties of mulberry flavonoids and their potential to alleviate oxidative stress, while also discussing endocrine/metabolic effects and antimicrobial activity. | https://doi.org/10.3390/ani10112076 |
| 10 | Ninety-six healthy Hu lambs with initial body weight about 27.59 +/- 3.03 kg were assigned to four treatments, with six replicates per treatment and four lambs per replicate. | Ensiled mulberry leaves (mulberry silage) were used. Replacement levels were 0, 20, 40, and 60% of maize silage, with concurrent soybean-meal adjustment. The trial lasted 75 days: 15 days acclimation and 60 days formal feeding. | Intake/digestibility: Feed intake and growth were not significantly impaired. Mulberry silage had higher CP and lower fiber than maize silage, changing diet composition and nutrient supply. Rumen/metabolism: Rumen pH and acetate:propionate ratio increased, while rumen ammonia decreased with increasing mulberry replacement. Microbial composition changed, including increased norank_f__F082. Rumen papillae became shorter as replacement increased, which the authors related to altered fiber characteristics of the diet. Growth: Initial/final body weight and growth rate were broadly maintained. The simple summary suggests 20-40% replacement could support growth while maintaining satisfactory digestion. Carcass: This paper did not focus on slaughter performance/carcass quality; its companion paper by Wang et al. (2022) provides those outcomes for the same broad dietary strategy. Meat quality: Detailed meat-quality measurements were not the main outcomes in this paper. FA/AA/flavor: Muscle fatty acids, amino acids, volatiles, and sensory quality were not central measurements. Antioxidant/physiology: The study provides physiological evidence through gastrointestinal morphology and fermentation rather than a detailed antioxidant/serum biomarker panel. | https://doi.org/10.3390/ani12111406 |
| 11 | Ninety-six healthy Hu lambs averaging about 27.5 kg were used. Four treatments contained six replicates with four lambs per replicate; subsets of animals were sampled and slaughtered at the end. | Mulberry was fed as silage produced from mulberry material. Maize-silage replacement was 0, 20, 40, or 60%. The study lasted 75 days, comprising 15 days acclimation and 60 days formal feeding. | Intake/digestibility: The paper reports no major adverse effect on growth, implying that intake/nutrient supply remained adequate across the range. Detailed digestibility/rumen outcomes are better covered by the companion Han et al. study. Rumen/metabolism: Rumen microbiota/fermentation were not the primary outcomes in this paper; the companion Han et al. paper provides those data. Growth: Initial/final body weight and overall growth performance did not differ significantly among treatments. Carcass: Slaughter performance was not significantly altered by mulberry-silage replacement, supporting feasibility without carcass penalty. Meat quality: Conventional pH45min and pH24h of longissimus dorsi were not significantly changed. Muscle ether extract increased, especially around the 40% treatment, indicating altered intramuscular lipid deposition without a broad deterioration of physical meat quality. FA/AA/flavor: Amino-acid proportions changed: the percentage of essential amino acids was lower in the 40% and 60% groups than in control/20%. In fatty acids, C16:1 increased in the 40% group, while C18:0 and C20:0 decreased. The study discussed possible flavor/nutritional implications, but volatile compounds and sensory panels were not measured directly. Antioxidant/physiology: Serum glucose and blood urea nitrogen decreased with mulberry silage. Other biochemical indices were largely maintained, suggesting no obvious systemic health impairment under the tested conditions. | https://doi.org/10.3390/ani12223164 |
| 12 | Not applicable as a single cohort. The reviewed evidence spans poultry, pigs, ruminants, fish and laboratory models. | Multiple forms were covered: whole/fresh material, leaf powder, fermented mulberry, silage, extracts, combinations with traditional medicines, and specific bioactive components. No single inclusion level or duration applies; the review explicitly notes that effective dose varies by species, physiological status, age, and purpose. | Intake/digestibility: The review reports that mulberry products can serve as supplementary protein/feed resources and may improve feed utilization and performance, but responses vary across species and preparation methods. Rumen/metabolism: It summarizes microbial modulation in the rumen and intestine and potential reductions in enteric methane, mainly through antimicrobial/bioactive effects. These are compiled from heterogeneous primary studies. Growth: Across animal studies, mulberry or extracts often improved or maintained growth/feed efficiency, but the review does not provide a single pooled effect estimate. Carcass: The review reports improvements in some carcass/product-quality studies, particularly in pigs and selected ruminants, but these findings should not be generalized to goat meat without direct evidence. Meat quality: Some source studies reported improved meat quality and reduced rancidity, but the review covers multiple species and product types, so it is background rather than direct goat/sheep meat evidence. FA/AA/flavor: The review reports changes in meat lipid composition and product quality in some studies, especially fermented mulberry/pig work, but is not a focused synthesis of small-ruminant fatty acids, amino acids, volatiles, or sensory responses. Antioxidant/physiology: A major theme is antioxidant, anti-inflammatory, antimicrobial and immunomodulatory activity of Morus compounds. These effects are supported by various animal and laboratory studies. | https://doi.org/10.3390/ani12243541 |
| 13 | No new animal cohort was used. The meta-analysis extracted outcome data from 28 eligible published papers on sheep, covering multiple breeds, ages, sexes, and production environments. | Mulberry was not the intervention. This is comparator evidence on feeding systems generally. No single dose/basal diet/duration applies because data came from multiple experiments. | Intake/digestibility: The meta-analysis focused on growth/carcass/meat outcomes rather than digestibility. It supports the broader principle that feed system alters nutrient supply and downstream production traits. Rumen/metabolism: Rumen fermentation and microbiota were not primary pooled outcomes. Growth: Pasture-only sheep had significantly lower ADG and slaughter live weight than stall-fed sheep; supplemented-pasture sheep were generally comparable to stall-fed sheep for major growth traits. Carcass: Pasture-only feeding reduced hot/cold carcass weights relative to stall feeding, while supplemented grazing produced carcass attributes broadly similar to stall feeding. Dressing percentage was less consistently different. Meat quality: Pasture or supplemented-pasture meat tended to have lower lightness and intramuscular fat but higher yellowness, shear force and protein than stall-fed meat. Thus, production and eating-quality trade-offs exist rather than a single universally superior feeding system. FA/AA/flavor: The paper discusses health-related meat composition in the context of feeding systems; detailed volatile/aroma or amino-acid outcomes are less central than physical meat quality and general nutritional attributes. Antioxidant/physiology: Systemic antioxidant markers were not the central pooled endpoints. | https://doi.org/10.3390/ani14182738 |
| 14 | Forty-five healthy Xiangdong black wether goats, about 6 months old and 18.2 +/- 1.6 kg, were used; 15 goats were assigned to each of three groups and housed individually. | Whole-plant/leaf-derived mulberry silage was used as the alternative protein/feed source. The key replacement levels were 0%, 50%, and 100% of soybean-meal protein. The paper contains an internal duration inconsistency: it states a total period of 90 days including 10-day adaptation and a '100-day formal period', while other methods refer to days 75-80 of the formal period. This should be flagged rather than silently resolved. | Intake/digestibility: Replacing soybean protein increased DMI, crude-protein intake, NDF intake and organic-matter intake. Most digestibility coefficients were unchanged, but organic-matter and ADF digestibility were lower in MS-100. Rumen/metabolism: Rumen fermentation/microbiota were not directly characterized. Nutritional interpretation therefore relies on intake/digestibility and blood responses. Growth: MS-100 significantly increased final body weight and average daily gain and improved feed-conversion-related performance compared with control under the reported conditions. Carcass: MS-100 increased carcass weight, while overall results supported mulberry silage as a practical protein replacement. The paper provides direct goat carcass evidence. Meat quality: Detailed pH/color/tenderness outcomes were limited. The authors describe improved meat quality primarily through amino-acid composition, especially at MS-50, rather than a full physical/sensory panel. FA/AA/flavor: MS-50 produced the highest concentrations of several amino acids, including aspartic acid, threonine, serine, glutamic acid, alanine, lysine and proline. Volatile compounds and sensory evaluation were not directly measured; fatty-acid data were not the major emphasis. Antioxidant/physiology: Plasma total protein, albumin and calcium increased in MS-100. Total antioxidant capacity increased in both mulberry groups, but MDA was also higher in MS-100, indicating that the redox response was not uniformly beneficial and should be interpreted carefully. | https://doi.org/10.3390/ani16050787 |
| 15 | Sixteen male Hu sheep, three months old and about 28.96 +/- 1.04 kg, were used; eight were allocated to control and eight to mulberry treatment. | Mulberry leaf powder was used. The treatment supplied 60 g/day mulberry leaf powder. The study lasted 56 days, including 14 days adaptation and 42 days formal experimental feeding. | Intake/digestibility: Feed intake and nutrient digestibility were not the main reported endpoints, so this paper provides limited evidence for those outcomes. Rumen/metabolism: The study examined fecal/hindgut rather than rumen microbiota. Mulberry altered microbial composition: Campylobacterota/Campylobacter/Mailhella increased, whereas Alloprevotella, Roseburia and Prevotellaceae UCG-003 decreased. These shifts show biological activity but are not automatically beneficial and should be interpreted cautiously. Growth: Growth performance/feed efficiency were not the central outcomes and no strong growth advantage was established. Carcass: No slaughter or carcass traits were reported. Meat quality: No physicochemical meat-quality measurements were reported. FA/AA/flavor: No muscle fatty-acid, amino-acid, volatile or sensory analyses were reported. Antioxidant/physiology: Blood alkaline phosphatase and GPT/ALT-related activity increased, total protein decreased, and GSH-Px/CAT showed upward trends. These changes demonstrate altered metabolism/antioxidant status, but some blood changes are not straightforward markers of improved health. | https://doi.org/10.3389/fanim.2024.1469850 |
| 16 | No livestock feeding cohort was used; this was a silage fermentation experiment. | Fresh mulberry leaves were processed into silage with defined bacterial inoculants and wheat bran. The key processing variable was inoculant combination rather than a dietary inclusion rate. All treatments received 10% wheat bran on a fresh-matter basis; the inoculated silage was assessed after fermentation rather than being fed in a long-term animal trial. | Intake/digestibility: Animal intake/digestibility were not measured. The study instead characterized silage dry matter, acids, pH and fermentation quality, which determine later feeding value. Rumen/metabolism: No in-vivo rumen data were collected. Microbial analysis was of the silage itself: combined inoculation increased beneficial Lactococcus/Lactobacillus and produced a favorable community profile. Growth: Growth performance was not measured. Carcass: Carcass traits were not measured. Meat quality: Meat quality was not measured. FA/AA/flavor: Muscle fatty acids, amino acids, flavor and sensory traits were not measured. Antioxidant/physiology: Antioxidant activity of the silage increased with fermentation, especially under favorable inoculant combinations. The A/AK groups also showed stronger lactic fermentation and lower ammonia-N than DK in key comparisons. | https://doi.org/10.3390/fermentation10040214 |
| 17 | No small-ruminant production trial was conducted. Rumen fluid/in-situ conditions were derived from two non-lactating rumen-cannulated Holstein cows. | Sun-dried Morus alba pomace and sun-dried mulberry leaves were compared. The experimental variable was 50% substitution of each mulberry by-product into TMRs across five NDF backgrounds, not a live-animal feeding dose. No long-term feeding duration applies. | Intake/digestibility: Mulberry leaf contained about 19% CP and 27.4% NDF, while pomace contained about 14.9% CP and 35.8% NDF. Leaf generally showed greater in-vitro NDF digestibility, and both materials showed substantial degradability, supporting their potential as ruminant feed resources. Rumen/metabolism: Fermentation responses depended strongly on basal NDF. The authors identified approximately 40-45% NDF combinations as promising for VFA production; ruminal pH remained in a physiologically acceptable range. Leaf treatments tended toward higher acetate proportions, while pomace produced relatively more propionate. Growth: Growth/feed efficiency were not measured in vivo. Carcass: Carcass traits were not measured. Meat quality: Meat quality was not measured. FA/AA/flavor: No muscle fatty-acid, amino-acid, flavor, volatile or sensory outcomes were measured. Antioxidant/physiology: No systemic antioxidant/blood outcomes were measured. | https://doi.org/10.3390/fermentation11120692 |
| 18 | Forty-four Guizhou crossbred black goats (Nubian male x Guizhou black goat female) averaging 33.43 +/- 0.55 kg were used, with 11 goats per treatment. | Three mulberry-feed forms were compared at the same nominal 40% level: pelleted TMR, fresh TMR and fermented TMR. Mulberry leaf comprised 40% of each experimental TMR. The trial lasted 85 days, including 10 days adaptation and 75 days formal data collection/feeding. | Intake/digestibility: The pellet TMR increased apparent digestibility of DM and CP (strongly) and EE/NDF relative to control. This treatment also supported better growth performance than control and fresh TMR in key comparisons. Rumen/metabolism: Rumen fermentation/microbiota were not deeply characterized, so the mechanism from feed form to digestibility is inferred from feed processing and apparent digestibility rather than directly measured rumen ecology. Growth: Average daily gain was significantly higher in the pelleted 40% mulberry TMR group than control and fresh-TMR group. The study therefore indicates that feed form modifies the production response even at the same mulberry inclusion level. Carcass: The paper focused on muscle quality rather than a complete carcass-yield analysis; five goats per group were slaughtered for tissue collection. Meat quality: Pellet TMR lowered L* and b* values in longissimus muscle and reduced shear force relative to control, consistent with altered color and improved tenderness. Intramuscular-fat/IMP-related traits were also interpreted as favorable. FA/AA/flavor: Pellet TMR increased total fatty acids while lowering total SFA and increasing UFA, MUFA and PUFA versus control. Total amino acids, EAA, NEAA and major fresh-tasting amino acids were higher in mulberry TMR groups, and IMP increased. These are strong flavor-precursor data, although volatile profiling and formal sensory evaluation were not reported. Antioxidant/physiology: Systemic antioxidant biomarkers were not the central outcome. The study's physiological/molecular contribution was expression of meat-quality-related genes. | https://doi.org/10.3390/foods11244032 |
| 19 | Not applicable as a single animal experiment. The review covers multiple goat breeds and production systems from published literature. | Mulberry was not the central topic. No single dietary inclusion level, basal diet, or duration applies. | Intake/digestibility: Feed intake/digestibility are not core review outcomes, although production systems and diet are recognized as contributors to goat meat variability. Rumen/metabolism: Rumen fermentation/microbiota are not major sections. Growth: Growth and production efficiency are discussed at industry/breed level rather than through a single intervention effect. Carcass: The review discusses carcass and production characteristics across goat breeds and notes that the goat-meat industry is less specialized and therefore produces variable carcass/product quality. Meat quality: It provides useful baseline information on goat meat composition, leanness, protein/fat content, pH, color, texture, water holding and other quality attributes. These baselines help interpret whether mulberry-fed goat meat values are biologically meaningful. FA/AA/flavor: The review summarizes goat-meat lipid/fatty-acid characteristics and their nutritional significance. It is not a mulberry-specific amino-acid or flavor intervention study, but it provides context for interpreting favorable or unfavorable changes in fat composition. Antioxidant/physiology: Antioxidant feed interventions are not its central focus. | https://doi.org/10.3390/foods12163130 |
| 20 | Two hundred 28-day-old Duroc x Landrace x Yorkshire piglets, with equal numbers of castrated males and females, were used. Five treatments each had four replicates of ten piglets. | Mulberry leaves were co-fermented with Lactobacillus and cellulase before dietary inclusion. Dietary inclusion was 0, 5, 10, 15 or 20% FML over a 28-day post-weaning period. | Intake/digestibility: Growth itself did not differ significantly, but feed-to-gain was lower in the 5% and 20% groups. Apparent nutrient digestibility was evaluated; the study's strongest benefits related to intestinal environment rather than a large growth response. Rumen/metabolism: This is a monogastric gut study, not rumen research. FML reduced duodenal pH, increased cecal acetate/propionate at higher inclusions, and altered cecal microbial diversity/composition; the 15% group had high Prevotella and trends for Lactobacillus/Bifidobacterium. Growth: No significant overall growth-performance improvement was detected, although some feed-efficiency changes occurred. Carcass: No slaughter/carcass data relevant to our small-ruminant review were reported. Meat quality: No meat-quality traits were measured. FA/AA/flavor: No muscle fatty-acid, amino-acid, volatile or sensory outcomes were measured. Antioxidant/physiology: The major physiological benefit was reduced diarrhea, with the greatest reported reduction in the 15% group, along with changed intestinal pH and SCFA production. Antioxidant meat endpoints were not studied. | https://doi.org/10.3389/fvets.2026.1874983 |
| 21 | Not applicable as a single cohort. Evidence comes from multiple ovine and caprine breeds, ages and production systems. | Mulberry was not a specific intervention. No single inclusion level/duration applies; numerous feeding systems and trial durations were compared. | Intake/digestibility: Digestibility was not the main pooled focus, but the review interprets how forage quality and concentrate supplementation change nutrient supply and growth. Rumen/metabolism: Rumen fermentation/microbiota are not central outcomes. Growth: Pasture-only finishing generally reduced ADG compared with stall feeding, while supplemented grazing often achieved similar or greater ADG. Carcass: Pasture-only systems generally reduced carcass yield, while supplemented grazing could produce carcass attributes similar to indoor systems. Time-limited grazing plus supplementation was discussed as a compromise strategy. Meat quality: Pasture increased flavor intensity and favorable fatty-acid content in many lamb/kid studies; supplemented grazing often maintained or improved sensory/nutritional traits. Sheep and goats differed in some color, tenderness and juiciness responses. FA/AA/flavor: Pasture-based systems generally increased beneficial n-3 PUFA and improved PUFA/SFA and n-6/n-3 indices compared with concentrate feeding. These data help interpret mulberry-induced fatty-acid changes but are not specific to mulberry. Antioxidant/physiology: Systemic antioxidant biomarkers were not a primary focus. | https://doi.org/10.3390/life13051215 |
| 22 | Not applicable as a single cohort. The review integrates sheep/goat meat studies across breeds, diets, ages, management systems and postmortem conditions. | Mulberry was not the central intervention. No single dietary dose or duration applies. | Intake/digestibility: Feed intake/digestibility were not central outcomes. Pre-mortem diet/feeding system is discussed as a driver of intramuscular lipid amount and composition. Rumen/metabolism: Rumen metabolism is relevant indirectly through fatty-acid transformation, but microbiota/fermentation were not the main synthesis endpoints. Growth: Growth performance was not the review's focus. Carcass: Carcass traits were not the core focus, although adiposity and fat deposition are relevant to intramuscular fat. Meat quality: The paper emphasizes that IMF affects juiciness, odor, flavor intensity and consumer perception; too little or too much fat can change sensory properties. It provides a framework for interpreting IMF changes in mulberry-fed animals. FA/AA/flavor: This is the paper's strongest area. It links fatty-acid distribution and lipid oxidation/degradation to volatile flavor compounds and characteristic sheep/goat odor. It also reviews branched-chain and other lipid-derived flavor contributors and effects of pre/postmortem management. Amino-acid-derived flavor is discussed less centrally than lipid-derived pathways. Antioxidant/physiology: Oxidative processes are important because lipid oxidation generates many aroma compounds; antioxidant status can therefore shift flavor formation and rancidity, although mulberry-specific antioxidant interventions are not evaluated. |  |
| 23 | One hundred eighty Xiangcun black finishing pigs with initial body weight 71.64 +/- 1.46 kg were used. There were five treatments, six pens per treatment and six pigs per pen. | Dried mulberry leaf powder was used. Dietary inclusion was 0, 3, 6, 9, or 12%. After a 7-day adaptation, the formal feeding trial lasted 50 days. | Intake/digestibility: Growth was not adversely affected at 3-9%; the paper does not provide ruminant digestibility information because pigs are monogastric. Rumen/metabolism: Rumen fermentation/microbiota are not applicable. The study instead measured systemic and muscle lipid-metabolic responses. Growth: Inclusion below 12% generally did not depress growth; the paper concluded that mulberry could be used as a forage/feed ingredient in finishing pigs. Carcass: Carcass traits were not the central contribution. Meat quality: The study focused on muscle lipid/oxidative characteristics rather than classic pH/color/tenderness outcomes. FA/AA/flavor: Mulberry increased muscle PUFA content, especially in longissimus dorsi. It also altered expression of lipid-metabolism genes. Amino-acid, volatile and sensory profiles were not the main outcomes. Antioxidant/physiology: Serum glutathione peroxidase activity and glutathione concentration increased linearly with mulberry inclusion. Muscle antioxidant-gene expression changed less consistently, but the paper concluded that antioxidant capacity and resistance to lipid oxidation were enhanced. | https://doi.org/10.1016/j.aninu.2020.08.005 |
| 24 | Forty lactating water buffaloes, about 600 +/- 50 kg, 4 +/- 1 lactations and approximately 90 +/- 20 days in milk, were used, with animals allocated to four treatments. | A commercial mulberry leaf extract containing about 5% flavonoids (reported as a mixture dominated by flavones/flavonols plus other polyphenols) was used rather than whole leaf biomass. Supplementation was 0, 15, 30, or 45 g/day for about 35 days (five weeks) on a common basal diet. | Intake/digestibility: Feed intake/digestibility were not the central direct endpoints in this paper; the focus was rumen fermentation and microbiota. Rumen/metabolism: At the highest dose, total VFA, acetate, propionate, microbial protein and NH3-N increased, while rumen pH was maintained. Bacterial alpha diversity decreased for several indices, and community composition shifted, with changes in Proteobacteria and multiple genera. Correlation analyses linked some bacteria with fermentation and antioxidant/production variables. Growth: Growth was not an endpoint because animals were lactating buffalo; related production work from the same animals addressed milk performance. Carcass: No slaughter/carcass outcomes were measured. Meat quality: No meat-quality outcomes were measured. FA/AA/flavor: No muscle fatty-acid/amino-acid/flavor outcomes were measured. Antioxidant/physiology: The paper references serum antioxidant associations and companion production data; its direct contribution is mainly rumen fermentation and bacterial composition rather than meat oxidative stability. | https://doi.org/10.7717/peerj.14309 |
| 25 | Twenty-four healthy castrated male Tan lambs were used. They averaged 75 ± 3 days of age and 15.3 ± 1.92 kg body weight at allocation. Six lambs were assigned to each of four dietary treatments. | Mulberry leaf silage was used as one of four feeding regimes. The exact ingredient percentages and nutrient composition were provided in Supplementary Table S1 rather than the main-text table reproduced in the uploaded PDF. The intervention lasted 80 days. For evidence extraction, the treatment should therefore be recorded as a mulberry-leaf-silage-based TMR rather than assigning an unsupported percentage from the main paper. | Intake/digestibility: Feed intake and digestibility were not the focus and were not reported as primary outcomes in this article. Rumen/metabolism: Rumen fermentation/microbiota were not measured in this paper. The study investigated muscle metabolomics rather than rumen mechanisms. Growth: Growth and feed efficiency were not reported as primary outcomes in this article and should not be inferred from the metabolomics results. Carcass: Carcass outcomes were not the focus. The paper concentrated on longissimus lumborum chemistry after slaughter. Meat quality: Meat crude protein did not differ significantly among diets. Intramuscular fat showed only a tendency to differ (p≈0.08), and ash was higher in mulberry-silage meat than corn-silage meat. Conventional pH, color, WHC and shear-force outcomes were not the main endpoint in this article. FA/AA/flavor: GC-MS identified 31 volatile compounds spanning alcohols, aldehydes, alkanes, esters and hydrocarbons. Compared with concentrate feeding, mulberry silage increased 3-hydroxydodecanoic acid; relative to corn silage it decreased 10,13-octadecadiynoic acid methyl ester, and relative to alfalfa silage it increased octaethylene glycol monododecyl ether. Overall, fewer volatile compounds changed than hydrophilic metabolites. UHPLC-QTOF-MS found 45 differential metabolites for MS vs CON, 40 for MS vs CS and 41 for MS vs AS. Mulberry-associated discriminants included L-palmitoylcarnitine, L-carnosine, His-Ser, Pro-Arg and propyl cinnamate; propyl cinnamate increased whereas Pro-Arg and His-Ser decreased under MS. The authors interpreted these changes as potentially favorable for taste, but no trained sensory panel validated that interpretation. Amino-acid-related metabolites were detected, but this was untargeted metabolomics rather than a conventional quantitative amino-acid composition assay. Antioxidant/physiology: No direct antioxidant-enzyme or oxidative-stability measurements were made. Some identified metabolites were discussed in relation to health or lipid oxidation, but this article is not direct antioxidant evidence. | https://doi.org/10.1016/j.fochx.2022.100269 |
| 26 | Forty healthy Tan lambs (Ovis aries) were selected at about 75 ± 3 days of age with an initial body weight of 15.3 ± 1.92 kg. Sex was not explicitly stated in the main method excerpt used here. Twenty lambs were assigned to each diet. Six lambs per group, selected around the average body weight, were used for terminal blood, slaughter and rumen/meat sampling. | Mulberry leaf silage was used. Fresh mulberry leaves were chopped to 2-3 cm, mixed with 1% glucose and Lactobacillus plantarum at approximately 10^6 CFU/g fresh material, and ensiled for 60 days. Mulberry leaf silage constituted 20% of diet DM and replaced 20% alfalfa silage; the remaining ingredients were identical. The experiment lasted 80 days, comprising 20 days of adaptation and 60 days of formal feeding. | Intake/digestibility: Dry-matter intake was similar between alfalfa- and mulberry-silage diets. No formal digestibility measurement was reported. Therefore, the paper supports maintenance of intake with silage replacement but not improved digestibility. Rumen/metabolism: Rumen pH, ammonia-N, total volatile fatty acids and major VFA proportions were generally unchanged. In contrast, 16S rRNA profiling showed clear shifts in alpha/beta diversity and taxonomic composition. Bifidobacterium, Lactobacillus and Schwartzia were enriched with mulberry silage, whereas Ruminococcaceae UCG-010 and Lachnospiraceae_XPB1014_group were reduced. Several taxa correlated with serum antioxidant and immune markers. These are association data and do not demonstrate microbial causality. Growth: Average daily gain, body weight and the DMI:ADG ratio were similar between diets. Thus, replacing alfalfa silage with 20% mulberry leaf silage maintained productive performance rather than producing a clear growth advantage. Carcass: Slaughter body weight, carcass weight and dressing percentage were similar. Most organ weights were also comparable, although head and spleen weights differed and kidney weight showed a tendency. Overall, the study indicates that 20% mulberry silage did not compromise carcass performance. Meat quality: Measured meat-quality traits were broadly similar between diets. pH at 45 min and 24 h and color variables did not differ significantly; L* at 45 min only tended to be lower with mulberry silage. The study therefore does not support a strong direct meat-quality improvement at this inclusion level. FA/AA/flavor: Fatty-acid, amino-acid, volatile and sensory profiles were not directly measured. The authors discussed possible implications for ruminal biohydrogenation based on microbial taxa, but muscle lipid composition was not quantified. Antioxidant/physiology: Mulberry silage increased serum catalase, glutathione peroxidase, superoxide dismutase and total antioxidant capacity and reduced malondialdehyde. IFN-γ increased, whereas IL-1β and IL-6 decreased; TNF-α and IL-2 were unchanged. Serum cholesterol, triglycerides, HDL and LDL were unchanged, whereas BUN was higher in the mulberry group. These results provide direct evidence for improved systemic antioxidant status and altered inflammatory/immune signaling. | https://doi.org/10.1186/s12866-021-02311-1 |
| 27 | Forty male Hu lambs, approximately three months old and 16.5 ± 0.6 kg, were used. They were randomly assigned to five groups of eight. For terminal slaughter, meat and liver measurements, only three lambs per group were randomly selected. | Mulberry leaf powder made from Morus alba var. multicaulis was used as a concentrate ingredient. MLP replaced combinations of conventional concentrate ingredients at 0, 15, 30, 45 or 60% of the concentrate. Concentrates were formulated near 250 g/kg crude protein, but digestible energy declined modestly at the highest dose. The experiment lasted 84 days, including 14 days of adaptation and 70 days of formal feeding. | Intake/digestibility: DMI was maintained at 0-30% MLP but was lower at 45-60% (about 801-817 vs 858-870 g/day in control/low-dose groups). Nutrient digestibility was not directly measured in this paper. Therefore, the data demonstrate an intake threshold rather than improved digestibility. Rumen/metabolism: Rumen fermentation was not directly measured in this publication. Total stomach and rumen weights were higher at 15-30% than control/high-dose treatments, suggesting altered rumen development, but this is anatomical rather than fermentation evidence. Growth: Average daily gain was similar at 0-30% (about 114-120 g/day) but declined to 100 and 87 g/day at 45 and 60% MLP. The findings support tolerance of moderate inclusion and impaired growth at excessive replacement. Carcass: Hot carcass weight and dressing percentage did not significantly differ, but GR measurement (fat/tissue depth) declined with increasing MLP. Total stomach weight and rumen weight were higher particularly at 15-30% MLP. Thus, moderate MLP maintained carcass performance while very high inclusion reduced growth. Meat quality: Muscle pH at 45 min and 24 h and L* were unaffected. Redness (a*) and chroma increased with MLP, and shear force declined markedly from 42.54 N in controls to 24.79 N at 60%, indicating improved instrumental tenderness. Cooking loss tended to decline. Pressing loss was inconsistent across doses. Muscle moisture, intramuscular fat, organic matter and protein were not significantly changed. FA/AA/flavor: Fatty-acid, amino-acid, volatile and sensory profiles were not measured. Therefore, this study should not be used as direct evidence for flavor chemistry or lipid composition. Antioxidant/physiology: MLP lowered ALT and LDH, with trends/reductions in AST, cholesterol and triglycerides, and increased HDL at higher inclusion. Hepatic antioxidant-related gene expression was strongly altered: Cu/Zn SOD and GPx increased with dose, and 15% MLP enhanced most targeted antioxidant-enzyme-related mRNAs except EPHX1. Higher doses also increased subsets of Nrf2, CAT, HO-1, PKC, MAFG and related genes. | https://doi.org/10.1139/cjas-2019-0119 |
| 28 | Twenty-four weaned Île-de-France lambs were used. They were approximately 60 days old, had an initial body weight of 15.48 ± 0.07 kg, and were housed individually. The paper does not explicitly restate sex in the extracted text. Animals were slaughtered at 32.2 ± 0.49 kg, at approximately 130 days of age. There were eight lambs per dietary treatment. | Sun-dried mulberry hay prepared from branches harvested after about 60 days of regrowth was used. The material was dried to below 20% moisture and ground through a 0.8-mm sieve before incorporation into the concentrate. The forage:concentrate ratio was 50:50. Sugarcane supplied the forage fraction. Mulberry hay was included at 0, 12.5 or 25.0% of total dietary DM and principally replaced ground corn, with other concentrate ingredients adjusted to maintain diet formulation. Animals were fed until they reached approximately 32 kg body weight; the same cohort is reported as being on trial for roughly 66 days in the companion physicochemical/sensory paper. | Intake/digestibility: Dry-matter intake was recorded and animals were weighed during the feeding period, but this article did not present a detailed intake or digestibility comparison as a major outcome. No digestibility trial was reported. Therefore, this paper should not be cited as direct evidence that mulberry improved digestibility. Rumen/metabolism: No rumen fermentation, rumen microbiota, volatile fatty acids, ammonia-N or ruminal biohydrogenation measurements were made. The discussion invoked ruminal biohydrogenation to interpret why dietary polyunsaturated fatty acids were only partly recovered in meat, but this mechanism was not directly measured. Growth: Growth was monitored until a common slaughter weight, but treatment-specific growth and feed-efficiency results were not reported in this article. Growth evidence from this paper should therefore not be extracted independently; performance information belongs to the associated feeding-trial publications. Carcass: Animals were slaughtered at a common target weight. This article did not focus on carcass yield or detailed carcass measurements, so it provides little independent evidence for carcass responses. Meat quality: Mulberry hay did not materially change the proximate composition of longissimus lumborum meat. Mean meat composition was approximately 757.9 g/kg moisture, 208.6 g/kg crude protein, 22.9 g/kg crude fat and 10.5 g/kg ash. Meat cholesterol averaged about 36.07 mg/100 g and was not affected by dietary mulberry. Physicochemical quality traits such as pH, color, water-holding capacity and tenderness were addressed in the companion Cirne et al. 2018 paper rather than this article. FA/AA/flavor: This is a key direct fatty-acid paper. Increasing mulberry hay increased several individual fatty acids, including pentadecanoic, heptadecanoic and arachidic acids, heptadecenoic acid, α-linolenic acid, γ-linolenic acid and eicosapentaenoic acid. Nervonic acid and conjugated linoleic acid showed quadratic responses, with estimated maxima near 16.98% and 12.56% mulberry inclusion, respectively. Total SFA, UFA, MUFA and PUFA were not significantly altered. Omega-3 fatty acids increased markedly and the omega-6:omega-3 ratio declined from about 20.99 in controls to 8.06 at 25% mulberry. Atherogenicity, thrombogenicity and related lipid indices were not significantly changed. Amino acids, volatiles and sensory traits were not measured in this article. Antioxidant/physiology: No direct antioxidant assays, oxidative-stability measurements or blood metabolites were reported in this article. The nutritional interpretation focused on fatty-acid composition rather than antioxidant physiology. | https://doi.org/10.4314/sajas.v49i1.3 |
| 29 | Twenty-four newly weaned Île-de-France lambs were used at approximately 60 days of age and 15.48 ± 0.07 kg initial body weight. Lambs were housed individually, eight per treatment, and slaughtered at 32.20 ± 0.49 kg. The feeding period was approximately 66 days. The paper does not clearly specify sex in the extracted main text. | Sun-dried and finely ground mulberry hay was incorporated into the concentrate portion of the total mixed feeding program. Diets contained 0, 12.5 or 25.0% mulberry hay on a total-diet DM basis, with a 50:50 concentrate:sugarcane ratio. Mulberry hay replaced ground corn and other concentrate ingredients were adjusted. Lambs were fed until the common target slaughter weight, for about 66 days. | Intake/digestibility: Feed intake was monitored, but detailed intake and digestibility outcomes were not the focus of this publication. No digestibility measurements were reported. The study should therefore not be used as direct digestibility evidence. Rumen/metabolism: No rumen fermentation, rumen microbiota or nutrient-metabolism biomarkers were measured. Growth: Animals were taken to slaughter at a common body weight, but treatment-specific growth and feed-efficiency data were not reported as the main evidence in this paper. Carcass: The paper did not identify adverse carcass effects attributable to mulberry in the meat-quality dataset, but detailed carcass yield outcomes were not its principal focus. It is better used for meat-quality and sensory evidence rather than carcass conclusions. Meat quality: Mulberry hay did not significantly affect pH at 45 min or 24 h, meat lightness (L*), redness (a*) or yellowness (b*), water-holding capacity, cooking loss, Warner-Bratzler shear force or sarcomere length. Across treatments, mean WHC was about 58.03%, cooking loss 41.95%, shear force approximately 2.6-2.8 kgf/cm² and sarcomere length about 1.60 μm. The meat remained within normal pH and tenderness ranges. FA/AA/flavor: No fatty-acid analysis was reported in this paper; those outcomes were published from the same experimental cohort in Cirne et al. (2019). A 30-consumer panel found no significant treatment effects on appearance, flavor, tenderness or overall acceptance; mean scores were approximately 7.29, 7.23, 7.51 and 7.48 on a nine-point hedonic scale. This provides direct evidence that up to 25% mulberry hay did not create an obvious sensory penalty in this trial. Antioxidant/physiology: No antioxidant, oxidative-stability or blood-metabolite outcomes were measured in this article. | https://doi.org/10.1080/1828051X.2017.1408435 |
| 30 | The in-vivo phase used 32 healthy male Small-tailed Han sheep, approximately four months old and 24.14 ± 0.78 kg, allocated to four groups of eight animals. The in-vitro phase used rumen fluid from Small-tailed Han sheep and six substrate ratios. | Unensiled mulberry leaves were combined with corn straw. The study focused on a complementary forage mixture rather than purified mulberry phytochemicals or silage. In the in-vivo complete diets, mulberry represented approximately 0, 8, 24 or 32% of the diet while corn straw declined correspondingly from 40 to 8%. Diets were formulated to have similar metabolizable energy and reasonably similar crude protein. The feeding experiment lasted 65 days, including a 10-day adaptation period. | Intake/digestibility: In vitro, higher mulberry proportions were positively associated with theoretical/cumulative gas production, microbial crude protein and in-vitro organic-matter digestibility, and T60-T100 produced higher total VFA/acetate at 72 h. In vivo, average daily feed intake was significantly higher in T60 than control (approximately 1260 vs 1174 g/day), but feed:gain did not differ. No in-vivo total-tract digestibility trial was reported. Rumen/metabolism: In-vitro fermentation showed ratio-dependent changes in gas production, VFAs, ammonia-N, microbial protein and IVOMD. These data demonstrate a measurable associative effect between corn straw and mulberry. The in-vivo experiment did not include rumen microbiome sequencing, so translation from in-vitro fermentation to host outcomes remains inferential. Growth: Average daily gain and final/live weight did not significantly differ among diets, whereas average daily feed intake increased in T60. Feed:gain remained similar. Thus, the major productive effect was enhanced intake rather than statistically confirmed faster growth. Carcass: Carcass weight, eye-muscle area and most fat depots did not differ significantly. Rib thickness was greater in T60/T80 and back-muscle thickness was greater in the higher-mulberry groups, suggesting an effect on muscle dimensions without a clear increase in carcass weight. Meat quality: Meat color and Warner-Bratzler shear force were not significantly changed. Water-holding capacity increased in the mulberry/corn-straw combination groups, especially T20, and longissimus dorsi drip loss was lower than control. Longissimus cooking loss was lowest in T20. These results support improved water retention, although carcass/meat measurements were based on only four animals per group. FA/AA/flavor: Fatty acids, amino acids, volatile compounds and sensory acceptance were not measured. Antioxidant/physiology: Blood urea nitrogen declined as mulberry proportion increased. Antioxidant assays showed greater DPPH/ABTS radical-scavenging capacity in higher-mulberry treatments. Liver catalase activity increased, and glutathione peroxidase was higher in T60/T80; SOD and MDA were not significantly different. These provide direct physiological/biochemical support for enhanced antioxidant capacity. | https://doi.org/10.5713/ab.24.0763 |
| 31 | Forty four-month-old male Hu sheep were randomly divided into two groups. Each group contained five pens with four sheep per pen (20 sheep per diet). After four months, one lamb per pen was randomly slaughtered, giving five slaughtered animals per treatment. Proteomics/PRM used three biological replicates per treatment. | Mulberry silage was used, compared directly with conventional corn silage. The reported ration contained approximately 21.3% concentrate, 25.5% peanut vines and 53.2% either corn silage or mulberry silage. Lambs were fed twice daily for four months. | Intake/digestibility: Feed intake and nutrient digestibility were not reported as primary outcomes. This paper should not be used to claim improved digestibility or feed efficiency. Rumen/metabolism: No rumen fermentation or microbiota measurements were made. Ruminal hydrogenation was discussed as a plausible explanation for fatty-acid changes, but it was not directly measured. Growth: Growth performance and feed conversion were not reported as primary outcomes, so the study provides little direct evidence for productive performance. Carcass: Mulberry silage markedly reduced subcutaneous fat mass (about 2.10 to 1.27 kg) and subcutaneous fat rate (about 8.81 to 5.38%) compared with corn silage, while intramuscular fat remained similar (about 1.80 vs 1.82 g/100 g). Meat tenderness was reported as unchanged. Thus, the strongest carcass finding is selective reduction of external fat rather than loss of intramuscular fat. Meat quality: Intramuscular fat concentration and tenderness were unchanged. The paper measured proximate composition of longissimus thoracis, but the principal reported quality effect was unchanged IMF/tenderness rather than broad improvement in conventional physicochemical traits. FA/AA/flavor: The key lipid analysis was performed on subcutaneous adipose tissue rather than directly on intramuscular muscle lipid. Mulberry silage reduced saturated and trans fatty acids and significantly increased α-linolenic acid by about 39% and oleic acid by about 37%, while linoleic acid fell by about 43%. Amino acids, volatile compounds and sensory attributes were not measured. For our review, this paper must be described accurately as adipose long-chain fatty-acid evidence, not as a direct muscle fatty-acid profile. Antioxidant/physiology: Most routine blood indices were unchanged. HDL increased significantly, whereas LDL, total cholesterol and triglycerides were not significantly altered. The study did not report a comprehensive antioxidant-enzyme panel in the same way as Wang & Luo (2021) or Hou et al. (2025). | https://doi.org/10.3390/foods13172739 |
| 32 | Sixty White Cashmere goats were used. All were wethers, with an initial body weight of 28 ± 1.05 kg and an average initial age of 230 days. Ten goats were allocated to each of six dietary treatments. At the end of the trial, only three goats per treatment were randomly selected for slaughter and terminal sampling, so rumen sequencing, rumen morphology, fermentation, and serum analyses were based on 18 goats in total. | Two forms of Morus alba leaf feed were compared: ensiled mulberry leaves (mulberry leaf silage) and sun-dried mulberry leaves. This direct comparison makes the study particularly useful for evaluating processing/form effects rather than mulberry inclusion alone. The ensiled diets contained 10%, 15%, or 20% mulberry leaf silage, whereas the sun-dried diets contained 10% or 15% sun-dried mulberry leaves in a typical TMR. Diets were formulated to meet nutrient requirements for growing goats. The study lasted 70 days, including a 10-day adaptation period and approximately 60 days of experimental feeding. | Intake/digestibility: The paper did not report a formal total-tract digestibility trial and did not present feed intake or feed-efficiency outcomes as principal results. Therefore, it should not be cited as direct evidence that mulberry improved intake or nutrient digestibility. Its contribution is primarily to rumen ecology, fermentation stability, and serum physiology. Rumen/metabolism: Rumen pH remained within a normal range (approximately 6.00-6.39), and feed form did not significantly alter total VFA, acetate, propionate, butyrate, acetate:propionate ratio, ammonia-N, CMCase activity, or rumen papilla/wall measurements. Total VFA averaged 112.67, 123.60, and 95.59 mmol/L in control, sun-dried, and ensiled groups, respectively (P=0.199). 16S rRNA sequencing identified 209 genera from 19 bacterial phyla, dominated by Firmicutes and Bacteroidetes. Alpha diversity did not differ significantly. Community structure differed significantly among feed types at phylum, genus, and OTU levels, but only Erysipelotrichaceae_UCG-009 differed significantly in relative abundance among the three main feed-form groups. Ruminococcus_2 abundance was positively correlated with butyrate concentration (r=0.715 after multiple-testing correction). Methanogens, ciliate protozoa, and anaerobic fungi were not significantly changed. Overall, the study supports microbial restructuring with largely stable bulk fermentation. Growth: Growth performance, average daily gain, final body weight response, and feed conversion were not reported as primary outcomes in this paper. The study therefore provides little direct evidence for growth or feed-efficiency effects. Carcass: Detailed slaughter yield, carcass weight, dressing percentage, and carcass composition were not investigated as outcome variables. Rumen tissues were collected at slaughter for morphology, but this is not equivalent to a carcass-quality assessment. Meat quality: No conventional meat-quality assessment was performed. The study did not report muscle pH, color, water-holding capacity, cooking loss, shear force, proximate composition, or sensory traits. It should not be used as direct evidence that mulberry improves goat meat quality. FA/AA/flavor: Muscle fatty acids, amino acids, volatile flavor compounds, and sensory characteristics were not measured. Although microbial pathways related to lipid and amino-acid metabolism were predicted, these predictions cannot substitute for direct muscle-composition measurements. Antioxidant/physiology: Most measured serum lipid-metabolism indices were unchanged: total cholesterol, triglycerides, HDL-C, LDL-C, lipase, lipoprotein lipase, and fatty-acid synthase did not differ significantly. Serum leptin was the major significant host response, declining from 7.26 ng/mL in controls to 4.75 ng/mL in the sun-dried group, while the ensiled group averaged 6.81 ng/mL (overall P=0.003). No direct antioxidant-enzyme panel or meat oxidative-stability test was performed. | https://doi.org/10.3390/microorganisms8070981 |
| 33 | Thirty healthy gelded Shannan White goats approximately three months old were used, with an initial body weight of 13 ± 2 kg. Fifteen goats were assigned to the control and fifteen to the experimental group. At the end of the feeding trial, only three goats from each group were randomly selected for the slaughter experiment and terminal muscle analyses. | The intervention was selenium-rich mulberry leaf powder. The material contained approximately 12.60% moisture, 15.52% crude protein, 2.5% crude fat, 19.4% crude ash, 8.9% crude fiber, 11.92 MJ/kg energy, and 0.12 mg/kg selenium. The experimental ration contained 15% selenium-rich mulberry leaf powder. Mulberry powder partly displaced distillers grains and small amounts of corn/soybean meal while crude protein and energy remained broadly similar between diets. The study lasted 60 days, including 15 days of preliminary feeding and 45 days of formal feeding. | Intake/digestibility: The study did not conduct or report a formal digestibility trial, and feed intake was not analyzed as an outcome. It therefore provides no direct evidence for effects on dry-matter intake or nutrient digestibility. Rumen/metabolism: No rumen fermentation, rumen microbiota, VFA, ammonia-N, nutrient-metabolism, or ruminal biohydrogenation measurements were made. Any explanation involving rumen lipid metabolism is therefore hypothetical in this study. Growth: Growth performance, average daily gain, and feed conversion were not reported as primary outcomes. Numerical differences in carcass weight cannot be treated as demonstrated growth effects because terminal sampling involved only three goats per group and carcass weight was not significantly different. Carcass: Carcass weight was numerically higher in the mulberry group (20.17 ± 2.48 kg) than in controls (17.64 ± 0.18 kg), but the difference was not significant (P=0.153). Loin-eye area was also numerically higher (10.49 ± 1.63 vs 9.49 ± 1.24 cm²; P=0.201). Hind-leg proportion was not significantly different (33.57 ± 6.23% vs 36.38 ± 1.22%; P=0.487). Thus, the paper does not demonstrate a statistically significant improvement in carcass yield or muscularity. Meat quality: The clearest meat-quality effect was poorer water retention: muscle water-loss rate increased significantly from 22.17 ± 6.60% in controls to 32.84 ± 6.38% with selenium-rich mulberry (P=0.004). pH at 1 h was higher in the treatment group (5.96 ± 0.30 vs 5.71 ± 0.18; P=0.050), whereas pH at 24 h was unchanged (5.58 ± 0.10 vs 5.60 ± 0.22; P=0.784). Cooking loss was not significantly different and marbling score was unchanged. This paper is useful because it shows that mulberry supplementation is not uniformly beneficial for all meat-quality traits. FA/AA/flavor: Nineteen fatty acids were detected in longissimus dorsi. The profile was dominated by MUFA, with approximate proportions of 28% SFA, 67% MUFA, and 5% PUFA in both groups. Most individual fatty acids and all major classes were not significantly changed. The only individually significant fatty acid was gamma-linolenic acid (C18:3n-6), which was slightly higher with selenium-rich mulberry (P=0.012). SFA, UFA, MUFA, PUFA, PUFA/SFA, n-6 PUFA, n-3 PUFA, and n-6:n-3 ratio were not significantly different. No amino-acid, volatile-compound, or sensory analyses were performed. Antioxidant/physiology: Muscle selenium was numerically higher with selenium-rich mulberry (0.0713 ± 0.006 vs 0.0660 ± 0.007 mg/kg), but this was not statistically significant (P=0.376). No antioxidant-enzyme activity, oxidative-stability test, blood-metabolite panel, gene-expression assay, or systemic physiological mechanism was directly measured. | https://doi.org/10.12160/j.issn.1672-5190.2023.01.008 |
| 34 | The experiment used 54 healthy female growing mutton sheep, described as white-headed Suffolk ram × Hu ewe crossbreds. Sheep were approximately 6 months old and averaged 30.41 kg at the start. Eighteen animals were assigned to each of three dietary groups. Six sheep per group, selected to represent the average group body weight, were slaughtered for tissue collection at the end of the trial. Depending on the assay, omics and biochemical analyses used smaller subsets of approximately 5-8 biological replicates per group. | Two forms of Morus alba leaves (cultivar/species designation 707) were used. Dried mulberry leaves (DMLs) were sun-dried for seven days. Fermented mulberry leaves (FMLs) were wilted to about 65% moisture, lightly crushed, inoculated with Lactobacillus plantarum (5 mL bacterial suspension per 100 g leaves; suspension concentration 1×10^8 CFU/mL), vacuum sealed and fermented for 30 days at approximately 27.5-28 °C. The 50-day feeding period was preceded by six days of adaptation. In the reported diets, FMLs accounted for 16.59% of the TR1 diet and DMLs for 7.11% of the TR2 diet. The remaining forage/concentrate ingredients differed among diets to maintain broadly similar nutrient composition: crude protein was 16.40, 16.70 and 16.60%, and metabolizable energy was 10.46, 10.63 and 10.30 MJ/kg for Con, TR1 and TR2, respectively. | Intake/digestibility: Average daily feed intake increased significantly with both mulberry treatments: approximately 1.79 kg in controls versus 2.31 kg with FMLs and 2.35 kg with DMLs (P<0.001). Feed:gain ratio was numerically lower with mulberry but was not significantly different. The study did not conduct a total-tract digestibility trial, so it should not be cited as direct evidence that mulberry improved nutrient digestibility. Rumen/metabolism: No rumen pH, volatile fatty acids, ammonia-N, rumen microbiota or direct nutrient-digestibility outcomes were measured. The study investigated systemic and muscle nutrient metabolism through metabolomics and transcriptomics rather than rumen fermentation. Therefore, any rumen-based explanation would be indirect and should come from other studies. Growth: Growth improved with mulberry feeding. Final body weight was 38.03 kg in controls, 42.05 kg in FML-fed sheep and 40.86 kg in DML-fed sheep (overall P=0.050). Average daily gain over 0-50 days increased from 156.11 g/day in controls to 233.89 and 205.56 g/day in FML and DML groups, respectively (P=0.001). Daily gain during days 25-50 was also higher in both mulberry groups. Serum growth hormone increased significantly (4.81, 5.84 and 7.28 for Con, FML and DML; P<0.001). Feed:gain ratio did not differ significantly. Carcass: Detailed slaughter yield, dressing percentage, carcass dimensions and fat-depot weights were not reported as primary outcomes in this article. Six sheep per group were slaughtered primarily to obtain muscle and adipose tissues for biochemical and omics analyses. This paper should therefore not be used as direct carcass-performance evidence. Meat quality: Conventional meat-quality traits such as postmortem pH, color, water-holding capacity, cooking loss, shear force and sensory tenderness were not measured. The authors speculated that reduced lipid peroxidation and lower expression of some genes might benefit meat redness and quality, but these claims were not validated with standard meat-quality measurements. This article is therefore mechanistic support, not direct physicochemical meat-quality evidence. FA/AA/flavor: The paper did not quantify conventional muscle fatty-acid classes, amino-acid composition, volatile flavor compounds or sensory characteristics. However, targeted muscle metabolomics identified 43 differential metabolites for control versus FML and 55 for control versus DML. Important changes included lower lipid-peroxidation products such as 8-HETE, 13-HOTrE, 8-iso-prostaglandin F2α, 11β-prostaglandin F2α and 8-iso-prostaglandin F2β, together with increased metabolites related to tryptophan metabolism, including 5-hydroxytryptophan and indoleacrylic acid. These are mechanistic metabolic markers and should not be presented as a conventional fatty-acid or flavor profile. Antioxidant/physiology: Both mulberry treatments markedly improved antioxidant status. Serum and muscle SOD, catalase, glutathione peroxidase and total antioxidant capacity were significantly higher, while serum and muscle malondialdehyde were reduced; FML generally produced the strongest response. For example, serum SOD increased from 58.72 in controls to 77.94 with FML and 66.56 with DML, and muscle SOD increased from 5.55 to 9.64 and 7.67, respectively. Immune responses also changed: total immunoglobulin and IgG increased most clearly with FML, TNF-α decreased with both treatments, and IgM decreased after FML, which the authors interpreted as an antibody-class transition. | https://doi.org/10.3389/fimmu.2022.1088850 |
| 35 | The study used the same experimental animals as Cui et al. (2023): 54 healthy female growing mutton sheep (white-headed Suffolk ram × Hu ewe crossbreds), approximately 6 months old and averaging 30.41 kg, with 18 sheep per group. Six sheep per group, selected around the group mean body weight, were slaughtered for tissue sampling. Omics analyses were performed on subsets of these samples. | Fermented mulberry leaves (FMLs) and dried mulberry leaves (DMLs) from Shansang 707 mulberry were used. DMLs were sun-dried for seven days. FMLs were wilted, lightly crushed, inoculated with Lactobacillus plantarum and fermented in vacuum-sealed bags for 30 days. Thus, the paper provides a direct comparison of processing form as well as mulberry supplementation. The treatment lasted 50 days after six days of adaptation. The main PDF refers the exact diet composition to Supplementary Table A.2 rather than reproducing all percentages in the article text. Because this is the same cohort as Cui et al. (2023), it should be interpreted as the same FML, DML and control feeding experiment, but the 2024 article should not be treated as a second independent dose trial. | Intake/digestibility: Feed intake and total-tract nutrient digestibility were not primary outcomes reported in this 2024 article. The paper focuses on tissue lipid metabolism. Therefore, any intake or growth effects should be cited from the companion Cui et al. (2023) paper rather than counted again from this publication. Rumen/metabolism: No rumen fermentation or rumen microbiota measurements were reported. The nutrient-metabolism work focused on serum, muscle and adipose lipid markers, subcutaneous-fat lipidomics and transcriptomics. Consequently, the paper provides host lipid-metabolism evidence rather than a rumen mechanism. Growth: Growth performance was not analyzed as a primary outcome in this paper. Because the same animals were used in Cui et al. (2023), the growth results belong to the shared experiment and should not be counted again. For the review, this publication should be cited for lipid-metabolism mechanisms, not as an additional independent growth study. Carcass: Detailed carcass yield and slaughter traits were not reported. The paper did, however, demonstrate a large reduction in longissimus dorsi crude fat: 15.05% in controls, 9.14% in the FML group and 6.75% in the DML group (P<0.001). This indicates lower muscle lipid content in the treatment groups, but it is not equivalent to a full carcass-fatness evaluation. Meat quality: Among proximate muscle components, dry matter, ash and crude protein were not significantly different, whereas crude fat decreased significantly and progressively from control to FML to DML. Conventional pH, color, water-holding capacity, cooking loss, shear force and sensory tenderness were not measured. Thus, the direct meat-quality contribution is limited mainly to proximate muscle fat rather than broader physicochemical quality. FA/AA/flavor: The paper did not provide a conventional GC profile of muscle fatty acids, amino-acid composition, flavor volatiles or sensory traits. Instead, wide-target LC-MS/MS lipidomics of subcutaneous fat identified major perturbations in triglycerides, diglycerides, glycerophospholipids, ether lipids, sphingolipids, free fatty acids and acyl-carnitines. These data are highly informative mechanistically but should not be reported as a standard edible-muscle fatty-acid composition. The work also identified changes in lipid species consistent with reduced unsaturated-fatty-acid oxidation and enhanced acyl-carnitine-mediated transport. Antioxidant/physiology: Serum total cholesterol, triglycerides, HDL and LDL were not significantly different overall, although FML tended to reduce triglycerides. Muscle and adipose free fatty acids and glycerol were also broadly unchanged. FML significantly altered several lipid-related hormones: adiponectin increased, while leptin, resistin and insulin decreased; DML significantly lowered leptin. Lipoprotein lipase in adipose tissue increased. Biochemical markers of β-oxidation/oxidative phosphorylation, including fatty-acid synthase, acetyl-CoA, CPT1-related signals, ATP/NADH and mitochondrial VDAC1, together with transcriptomic GSEA, supported enhanced lipid degradation and mitochondrial energy metabolism. | https://doi.org/10.1093/jas/skae076 |
| 36 | The main trial used 36 clinically healthy multiparous Damascus does in the last third of pregnancy, aged 4-7 years, parity 2-5, and averaging 42.13 ± 0.27 kg. Twelve does were assigned to each diet. A separate digestibility/rumen trial used nine adult Damascus bucks, approximately 24 months old and about 50 kg, with three bucks per diet. Offspring numbers reflected reproductive outcomes: 8, 10 and 12 kids were born in the ML0, ML25 and ML50 groups, respectively. | Healthy Morus alba leaves were collected from mature trees, shade/air-dried at approximately 25-28 °C with ventilation, ground through a 1-mm screen and stored for diet preparation. HPLC characterized the phenolic fraction. The dominant compounds included chlorogenic acid (3976 µg/g), gallic acid (1144 µg/g), syringic acid (764 µg/g), catechin (635 µg/g), rutin (340 µg/g), coumaric acid (120 µg/g) and ellagic acid (90 µg/g), with smaller quantities of several other phenolics/flavonoids. The basal ration consisted of a concentrate feed mixture contributing about 60% of intake, 250 g rice straw/goat/day, and fresh berseem offered ad libitum. ML25 replaced 25% of the concentrate feed mixture with dried mulberry leaves; ML50 replaced 50%. The main feeding trial lasted 150 days (last 60 days of gestation + 90 days of suckling). Postpartum reproductive performance was evaluated during the subsequent breeding season. The digestibility trial used 14 days of adaptation plus a 7-day collection period. | Intake/digestibility: In the separate buck trial, mulberry significantly improved digestibility of organic matter, crude protein, crude fiber, NDF and ADF in a dose-dependent manner. For example, CP digestibility increased from 63.8% in ML0 to 65.8% in ML25 and 68.2% in ML50; NDF digestibility increased from 53.3 to 55.5 and 58.4%, respectively. Dry-matter digestibility did not differ significantly. TDN and DCP values increased modestly, while dry-matter intake and TDN intake did not differ significantly. Because only three bucks per treatment were used, the precision and generalizability of these digestibility estimates are limited. Rumen/metabolism: Rumen-fluid measurements in the nine-buck trial showed higher total volatile fatty acids in mulberry groups, especially 3 and 6 h after feeding, while ruminal ammonia-N was lower at 3 and 6 h. Rumen pH followed the expected post-feeding decline and recovery and remained within a physiologically acceptable range. No rumen microbiome sequencing was performed. The authors attributed improved fermentation partly to phenolics/flavonoids, but the direct evidence is limited to pH, VFA and ammonia-N; specific microbial mechanisms were not measured. Growth: For the does, body weight was similar during late pregnancy and near parturition, but the ML50 group had higher body weight during the first and second months postpartum. For individual kids, weaning weight increased significantly (15.83, 16.23 and 16.91 kg for ML0, ML25 and ML50; P=0.048), whereas individual total gain and ADG were numerically higher but not statistically significant. At the litter level, weaning litter weight, total litter gain and litter ADG increased significantly, especially with ML50. Feed efficiency for 4%-fat-corrected milk per unit of DM or TDN improved with mulberry, while efficiency per unit DCP decreased slightly at higher inclusion. Carcass: No slaughter, dressing percentage, carcass dimensions or meat-yield traits were measured because this was a dairy/reproductive goat study. It should not be used as direct evidence for carcass performance. Meat quality: No meat pH, color, water-holding capacity, cooking loss, shear force, proximate meat composition or other meat-quality outcomes were measured. The paper is therefore supporting evidence for nutrition, rumen function and whole-animal physiology rather than direct meat-quality evidence. FA/AA/flavor: No muscle fatty-acid profile, amino-acid composition, volatile/flavor analysis or sensory evaluation was performed. The study measured milk composition rather than meat chemistry. Daily milk yield and 4% FCM increased only numerically and non-significantly. Milk fat and protein percentages declined with mulberry, while absolute fat yield increased significantly and protein yield was lower in the mulberry groups. Thus, it should not be used to make claims about meat flavor or nutritional composition. Antioxidant/physiology: Blood biochemistry was measured in the does. The paper reports that 50% mulberry increased total antioxidant capacity and altered cholesterol/triglyceride-related measures, while many other blood constituents were not significantly different; blood urea nitrogen and protein-related measures also changed. Reproductive physiology improved: the ML50 group reached 100% estrus/mating response, shortened the interval to estrus, and showed higher progesterone at later post-mating time points. Pregnancy, fecundity, prolificacy and twinning-related outcomes generally favored mulberry, although some endpoints did not increase monotonically (for example, conception rate was numerically highest in ML25). | https://doi.org/10.1007/s11250-026-05109-3 |
| 37 | Eight growing goats were used. The available paper record identifies goats as the experimental animals; detailed breed/sex information is not consistently reported in accessible metadata and should not be inferred. | Mulberry was offered as fresh leaves, dried leaves, whole branches, and a dried-leaf/maize-bran mixture. The four treatments compared dried mulberry leaf plus maize bran (1:1), fresh leaves, dried leaves, and whole mulberry branches. The study also evaluated leaves of different maturity; exact period length should be taken from the original paper if used quantitatively. | Intake/digestibility: Dry-matter intake was high for the leaf-based diets, and digestibility differed by form; reported DM digestibility for the first three principal leaf diets was approximately 55.7, 65.6 and 63.0%. Intake and digestibility declined as leaves matured, showing that plant maturity is an important source of heterogeneity. Rumen/metabolism: Rumen fermentation variables and microbiota were not directly measured. The nutritional interpretation is based on intake and whole-tract digestibility rather than rumen mechanism measurements. Growth: Daily gains reported across the four treatments were approximately 26, 92, 86 and 86 g/day, indicating poorer performance for the dried-leaf/maize-bran treatment than for the other mulberry forms in this small study. Carcass: Carcass or slaughter traits were not measured. Meat quality: Physicochemical meat quality was not measured. FA/AA/flavor: Fatty acids, amino acids, volatiles and sensory outcomes were not measured. Antioxidant/physiology: Antioxidant, blood or physiological biomarkers were not evaluated. | Bibliographic record; no DOI located |
| 38 | The animal phase used five growing goats with mean body weight about 17.3 kg. Breed and sex should be verified from the original article before subgroup interpretation. | Chopped mulberry foliage was ensiled. Molasses and rice bran were tested as ensiling additives; approximately 5% molasses produced favorable silage quality and was used to support the feeding evaluation. Goats received natural grass plus 0, 250, 500 or 750 g/day mulberry silage, or mulberry silage offered ad libitum as the sole forage treatment. This produced a wide range of dietary mulberry exposure. | Intake/digestibility: Dry-matter intake rose from about 2.70 to 3.41 kg DM/100 kg body weight as mulberry silage increased to roughly 40% of diet DM; intake on 100% mulberry silage was lower than the best mixed diet but remained substantial. DM and OM digestibility were not significantly different among treatments. Rumen/metabolism: Rumen fermentation was not the central endpoint. Nitrogen utilization was evaluated: nitrogen retention showed a curvilinear response and was high on the all-mulberry-silage treatment, supporting favorable protein value. Growth: The trial focused on intake/digestion rather than a long-term growth endpoint; therefore it should not be used to make strong claims about ADG or feed conversion. Carcass: No slaughter or carcass outcomes were measured. Meat quality: No meat-quality outcomes were measured. FA/AA/flavor: No meat fatty-acid, amino-acid, volatile or sensory outcomes were measured. Antioxidant/physiology: No antioxidant or blood-metabolite panel was reported as a major outcome. | https://www.lrrd.org/lrrd17/2/ba17015.htm |
| 39 | Wether sheep were used for the digestibility evaluation. The paper is a feed-value study rather than a growth or meat-quality experiment; detailed animal number should be taken from the full methods if used quantitatively. | Dried mulberry leaves were evaluated as the feed ingredient. The study used mulberry as a partial diet supplement/replacement. The leaf material contained about 201 g crude protein/kg DM, 268 g NDF/kg DM and showed very high in-vitro true DM digestibility (~89.8%). | Intake/digestibility: Digestibility of DM, crude protein and crude fibre was generally maintained when mulberry partly replaced conventional ingredients, supporting high feeding value rather than a consistent treatment advantage. Rumen/metabolism: Rumen microbiota or detailed fermentation kinetics were not directly assessed in the feeding component. Growth: Long-term growth performance and feed efficiency were not the main outcomes. Carcass: Carcass traits were not measured. Meat quality: Meat quality was not measured. FA/AA/flavor: Meat composition, fatty acids, amino acids, flavor and sensory traits were not measured. Antioxidant/physiology: No systemic antioxidant or blood-metabolite panel was central to the study. | https://doi.org/10.1007/s11250-008-9149-y |
| 40 | Three non-castrated Pelibuey male lambs fitted with rumen cannulas were used. | White mulberry leaves were incorporated as a dried feed ingredient in the ration. The basal diet was approximately 90% concentrate and 10% sorghum straw; white mulberry replaced part of the straw at 0, 2.5 or 5% of total ration. Diets were formulated at similar crude protein (~14%) and metabolizable energy (~10.8 MJ/kg DM). | Intake/digestibility: Whole-animal intake/digestibility was not the main outcome. Nylon-bag degradation showed greater potential degradation at the intermediate inclusion, while degradation rate/effective degradation were generally not strongly altered. Rumen/metabolism: The study directly measured ruminal in-situ degradation but not microbial community structure or detailed VFA profiles. Growth: Growth and feed efficiency were not assessed as meaningful endpoints. Carcass: Carcass traits were not measured. Meat quality: Meat quality was not measured. FA/AA/flavor: Fatty acids, amino acids, flavor and sensory traits were not measured. Antioxidant/physiology: Antioxidant and blood physiology were not measured. | https://doi.org/10.1007/s11250-011-9797-1 |
| 41 | Sheep/lambs were the experimental model. The accessible abstract metadata does not provide enough detail to state breed, sex, sample size or exact initial weight reliably, so these should be checked in the original paper before quantitative pooling. | Mulberry leaves were used as the protein-rich replacement feed. Mulberry progressively replaced rapeseed meal while ammoniated rice straw remained the basal forage. Exact dose groups and trial duration should be taken from the original paper for dose-response extraction. | Intake/digestibility: Published summaries and later reviews report that mulberry replacement maintained or enhanced feed intake and supported nutrient use compared with conventional rapeseed-meal supplementation. Rumen/metabolism: The paper addresses ruminant feeding performance but is not a modern microbiome study; microbial community composition was not measured. Growth: The study is repeatedly cited as showing that mulberry replacement supported sheep growth performance on a low-quality straw basal diet. Exact ADG estimates should not be quoted without the original tables. Carcass: No evidence from this paper should be used for detailed carcass traits unless verified in the full text. Meat quality: Physicochemical meat quality was not a central endpoint. FA/AA/flavor: No modern muscle fatty-acid, amino-acid, volatile or sensory profiling was reported. Antioxidant/physiology: Antioxidant and omics biomarkers were not evaluated. | https://doi.org/10.1016/S0921-4488(00)00180-2 |
| 42 | Ten healthy crossbred sheep (Dorper male × Small-tailed Han female background) with mean body weight 60.0 ± 1.73 kg were used. | A characterized mulberry leaf flavonoid extract was used rather than whole leaf biomass. The MLF dose was 2 g/day per sheep; resveratrol was 0.25 g/day per sheep. All animals received the same basal diet across change-over periods. | Intake/digestibility: MLF and resveratrol tended to improve apparent digestibility of DM, OM, NDF, ADF and nitrogen; the paper reports significant improvements overall, with some effects stronger for resveratrol. Metabolizable energy was improved. Rumen/metabolism: Methane and CO2 were directly measured with an open-circuit respiration system. Both polyphenols reduced methane intensity metrics, although statistical strength differed by compound. Nitrogen retention was not improved. Growth: Growth performance was not the study's purpose and was not evaluated as a long-term production endpoint. Carcass: Carcass traits were not measured. Meat quality: Meat quality was not measured. FA/AA/flavor: No meat fatty-acid, amino-acid or flavor profiling was performed. Antioxidant/physiology: The study focused on gas/energy/nitrogen metabolism rather than antioxidant blood endpoints. | https://doi.org/10.1016/j.aninu.2015.12.008 |
| 43 | Eighteen lambs with initial body weight 14.4 ± 3.35 kg were used, six per treatment. | Mulberry foliage was used directly as a dietary supplement; one treatment combined mulberry with Leucaena. Mulberry foliage comprised 30% of total diet as a substitution for UR; the mixed treatment contained 30% foliage total, 15% mulberry + 15% Leucaena. Diets were formulated to be iso-energy and iso-protein. | Intake/digestibility: Mulberry and mixed foliage increased DMI by about 15.4% and 9.9%, and NDF digestibility by about 17.5% and 14.5%, respectively, relative to UR. Nitrogen excretion/retention was broadly similar. Rumen/metabolism: Rumen fermentation, microbial nitrogen yield and estimated methane production were evaluated. Overall rumen-fermentation responses were similar among treatments; ammonia concentrations suggested extensive ruminal protein degradation. Growth: Average daily gain was similar among treatments (around 71.4 g/day overall), while the foliage treatments increased intake and supported body-weight gain comparable with the conventional supplement. Carcass: Carcass traits were not evaluated. Meat quality: Meat quality was not evaluated. FA/AA/flavor: No muscle fatty acids, amino acids, volatiles or sensory outcomes were measured. Antioxidant/physiology: No antioxidant or blood-metabolite outcomes were central to the study. | https://doi.org/10.4314/sajas.v54i3.04 |
| 44 | Forty-five Hu sheep with mean initial body weight 26.42 ± 3.57 kg were randomly assigned to three groups (approximately 15/group). | Fermented mulberry leaves were used; the intervention combines mulberry composition with effects of fermentation processing. Fermented mulberry leaves replaced 0, 10 or 20% of concentrate. The pretrial lasted 7 days and the formal feeding period 90 days under heat-stress conditions. | Intake/digestibility: The 10% replacement improved apparent digestibility of ADF and crude protein and affected intake/efficiency. Responses were not uniformly stronger at 20%, supporting a non-linear dose relationship. Rumen/metabolism: Rumen fermentation was directly measured; acetate, propionate, valerate and ammonia-N changed with treatment. 16S/ITS sequencing showed bacterial and fungal shifts, including increased Prevotellaceae_UCG_003 and unclassified Selenomonadaceae at 10% and changes in several fungal taxa. Growth: Average daily gain increased during the first month in the 10% group, and feed-conversion/feed cost responses favored moderate replacement; the advantage was not a simple linear dose effect across the full 90 days. Carcass: Carcass outcomes were not the main endpoint. Meat quality: Meat quality was not measured. FA/AA/flavor: Fatty acids, amino acids, volatiles and sensory traits were not measured. Antioxidant/physiology: The experiment was conducted during heat stress, making the results physiologically relevant to environmental challenge, but it did not provide a full oxidative-stress/meat-stability panel. | https://doi.org/10.1007/s00253-026-13819-9 |
| 45 | Two experiments were used. Digestibility was evaluated in 20 castrated Phanrang rams (23–25 kg). The cattle performance trial used 20 Laisind crossbred bulls, approximately 18 months old and 184 kg, five animals per treatment. | Fresh chopped mulberry leaves were used. In cattle, fresh mulberry leaves supplied 0–15% of total dietary DM and partially replaced cottonseed over 84 days. | Intake/digestibility: Mulberry leaves contained about 22.3% CP and 31.1% NDF on a DM basis and showed superior digestibility to the comparator grasses. In cattle, intake and feed utilization were assessed across replacement levels. Rumen/metabolism: Detailed rumen microbiota were not measured; the support is based on digestibility and performance rather than modern microbiome endpoints. Growth: Partial cottonseed replacement was compatible with growing-cattle performance and provided practical evidence that fresh mulberry can replace part of a conventional protein feed. Carcass: Carcass traits were not a primary endpoint. Meat quality: Meat quality was not measured. FA/AA/flavor: No meat fatty-acid, amino-acid, volatile or sensory measurements were made. Antioxidant/physiology: No modern antioxidant/metabolic-omics panel was included. | https://doi.org/10.5713/ajas.2011.90328 |
| 46 | Four rumen-fistulated Brahman × Thai native beef cattle (420 ± 15 kg) were used. | Mulberry leaves were processed into pellets. Pellets were supplemented at 0–600 g/day. Concentrate was offered at 5 g/kg body weight and rice straw ad libitum. Four 21-day periods were used, with metabolism-crate collection in the final 7 days of each period. | Intake/digestibility: Rice-straw and total DMI increased with supplementation, peaking at 600 g/day. Apparent digestibility of DM, OM, CP, NDF and ADF increased, especially at the highest dose; nitrogen balance also improved. Rumen/metabolism: Rumen pH and temperature remained stable. Ammonia-N and BUN increased with supplementation; total VFA and selected VFA proportions increased, indicating altered fermentative nutrient capture. Growth: This metabolism experiment did not provide long-term ADG/feed conversion as a primary endpoint. Carcass: Carcass traits were not measured. Meat quality: Meat quality was not measured. FA/AA/flavor: No muscle composition/flavor outcomes were measured. Antioxidant/physiology: BUN was measured as a nitrogen-metabolism indicator; broader antioxidant physiology was not evaluated. | https://doi.org/10.1016/j.anifeedsci.2012.03.020 |
| 47 | Four rumen-fistulated Brahman × native crossbred beef cattle with initial body weight 420 ± 15 kg were used. | Mulberry leaf meal was pelleted with urea as a concentrated fermentable nitrogen/feed supplement. Pellets were supplied at 0–600 g/head/day with rice straw offered ad libitum; cattle also received concentrate. The crossover periods followed a standard rumen-metabolism design. | Intake/digestibility: Rice-straw and total intake increased with increasing pellet supplementation, with the strongest response at 600 g/day. Rumen/metabolism: Rumen pH and temperature remained within normal ranges; ammonia-N increased, and total/cellulolytic bacterial populations were enhanced at the high pellet level. Methanogen diversity was not clearly altered. These data directly support changes in rumen ecology but not necessarily methane output. Growth: Long-term growth performance was not the central endpoint. Carcass: Carcass traits were not measured. Meat quality: Meat quality was not measured. FA/AA/flavor: No meat composition or sensory outcomes were measured. Antioxidant/physiology: The main physiological measurements concerned ruminal nitrogen and microbial ecology rather than systemic antioxidant status. | https://doi.org/10.5713/ajas.2011.11270 |
| 48 | No live production animal cohort was used. This is a feed-level ensiling/in-vitro study. | Chopped mulberry leaves were ensiled with carbohydrate or microbial additives. Treatments were stored at approximately 25 °C for 90 days before analysis. | Intake/digestibility: DM, NDF, ADF and WSC were broadly similar among treatments. The work used in-vitro gas production rather than animal total-tract digestibility; 72-h gas production was highest with L. rhamnosus treatment. Rumen/metabolism: Silage organic-acid patterns and microbial/yeast counts were affected: acetic acid was lower with 2–3% glucose and propionic acid/yeast were lower with P. acidilactici. No in-vivo rumen microbiome was measured. Growth: Growth was not measured. Carcass: Carcass traits were not measured. Meat quality: Meat quality was not measured. FA/AA/flavor: No meat composition/flavor outcomes were measured. Antioxidant/physiology: Antioxidant and blood responses were not measured. | https://doi.org/10.17582/journal.pjz/2017.49.6.2271.2277 |
| 49 | No live animal feeding trial was performed; this is a feed-level silage experiment. | Mulberry leaves were ensiled with biological and carbohydrate additives. Silage was prepared under controlled conditions and evaluated after storage and during aerobic exposure; no dietary inclusion level in animals applies. | Intake/digestibility: In-vitro digestibility was evaluated. Additives altered preservation characteristics; LAB inoculation was particularly useful for fermentation/aerobic stability, while simple sugar sources did not consistently improve all outcomes. Rumen/metabolism: The microbial mechanism was evaluated at silage level rather than in the rumen. Fermentation acids and aerobic spoilage stability were the primary microbial outcomes. Growth: Growth was not measured. Carcass: Carcass traits were not measured. Meat quality: Meat quality was not measured. FA/AA/flavor: Meat composition and sensory traits were not measured. Antioxidant/physiology: Animal antioxidant or blood responses were not measured. | https://doi.org/10.5713/ajas.19.0420 |
| 50 | Thirty-six mid-lactation dairy cows were randomly allocated to three groups (12/group). | Ensiled mulberry branches and leaves were used, not leaves alone. The intervention supplied 0, 5 or 10% MBL silage in TMR. The trial lasted eight weeks, including two weeks adaptation. | Intake/digestibility: The paper focused on fermentation and milk production rather than a formal total-tract digestibility trial. Rumen/metabolism: The 10% group had higher propionate and a lower acetate:propionate ratio. 16S profiling showed shifts including lower Bacteroides/Prevotella and increased Succinivibrionaceae_UCG-001; several Firmicutes taxa correlated positively with propionate. Growth: Growth does not apply to lactating cows. Milk yield and milk fat increased in the high MBL group; feed-conversion response showed a favorable trend. Carcass: Carcass traits were not measured. Meat quality: Meat quality was not measured. FA/AA/flavor: No meat fatty-acid/amino-acid/flavor outcomes were measured; milk composition rather than meat was relevant. Antioxidant/physiology: No broad antioxidant panel was central to this study. | https://doi.org/10.3390/fermentation8020086 |
| 51 | Twelve healthy multiparous Holstein cows (442.83 ± 86.87 kg; milk yield 12.55 ± 1.91 kg/day; 150 ± 47 days in milk) were randomized to two groups of six. | Mulberry leaves were fed as a dietary supplement alongside the basal ration. The treatment supplied approximately 300 g mulberry leaves/cow/day. The study included an adaptation period followed by a 28-day experimental phase. | Intake/digestibility: DMI was not significantly altered and a formal total-tract digestibility trial was not the primary design. Rumen/metabolism: Rumen pH and ammonia-N were stable. Total VFA tended to rise; propionate and butyrate increased and acetate:propionate decreased. 16S sequencing showed treatment-related bacterial shifts, including enrichment of taxa associated with propionate/fibre metabolism. Growth: Milk yield, feed efficiency and major milk components were largely unchanged at this modest supplement dose. Carcass: Carcass traits were not measured. Meat quality: Meat quality was not measured. FA/AA/flavor: No meat composition or flavor outcomes were measured. Antioxidant/physiology: Routine serum AST, ALT, ALP, BUN and creatinine were not adversely altered, supporting short-term metabolic tolerance at the tested dose. | https://doi.org/10.1080/1828051X.2024.2375394 |
| 52 | Forty male Ossimi lambs, 4–5 months old, with initial body weight about 20.6 ± 0.8 kg were used, approximately 10 lambs per treatment. | Dried mulberry leaves were incorporated directly into the ration. Mulberry leaves were included at 0, 5, 10 and 15% of the diet. The exact adaptation/formal period is reported in the source paper and should be retained as published when used quantitatively. | Intake/digestibility: Mulberry inclusion improved apparent digestibility of DM, OM and CP at moderate/high levels. Reported total digestible nutrients were about 68.4% in control and 71.6/71.3% at 10/15% inclusion. Rumen/metabolism: Rumen fermentation indices were improved at moderate inclusion; the study reports effects on rumen characteristics but did not use microbiome sequencing. Growth: Daily gain, total gain and final body weight improved with mulberry inclusion, and the 15% treatment showed the most favorable feed-conversion/efficiency response. Carcass: Detailed carcass traits were not a principal outcome. Meat quality: Physicochemical meat quality was not a principal outcome. FA/AA/flavor: No detailed muscle fatty-acid, amino-acid, volatile or sensory profiling was reported. Antioxidant/physiology: Blood constituents and antioxidant status were evaluated, providing direct physiological evidence alongside growth and digestibility responses. | https://doi.org/10.1007/s11250-026-05192-6 |
| 53 | Wethers in an in-vivo digestibility comparison of mulberry foliage with alfalfa hay and oat hay. | Mulberry foliage compared directly with alfalfa and oat hay; study focused on total-tract and ruminal digestibility. | Directly measured intake/digestibility and rumen disappearance showed that mulberry foliage can function as a high-quality ruminant forage and was comparable with alfalfa on several nutritive measures. | https://doi.org/10.1016/j.anifeedsci.2006.11.016 |
| 54 | Meta-analysis across poultry and livestock studies rather than a single animal cohort. | Multiple mulberry-leaf forms and doses across species; search covered PubMed, Embase and Scopus through December 2022. | Synthesized growth, production, gut-microbiota and immunological outcomes across species; not designed around carcass-to-meat or flavor outcomes in sheep/goats. | https://doi.org/10.5713/ab.23.0449 |
| 55 | No single animal cohort; broad review of mulberry as a forage crop across livestock systems. | Covers Morus species, agronomy, nutritive value, conservation/preservation and livestock feeding applications. | Provides broad forage-production and feeding context rather than a focused synthesis of small-ruminant carcass, meat-quality, sensory and mechanistic evidence. | https://doi.org/10.1016/j.anifeedsci.2025.116558 |
| 56 | Ruminant degradation experiments comparing mulberry plant fractions harvested at different maturities. | Whole-plant/stem/leaf fractions harvested at 3, 5, 7 and 9 weeks; no meat-quality intervention. | Dry-matter and crude-protein disappearance changed with plant maturity, particularly in whole-plant and stem fractions; the study demonstrates that harvest maturity alters apparent feeding value. | https://doi.org/10.5713/ajas.2005.1569 |
| 57 | Eighteen sheep (approximately 18 months; 22.5 ± 1.1 kg) were allocated to three dietary groups following in-vitro screening. | Sun-dried mulberry leaves used alone or in combination with Eupatorium adenophorum; concentrate replacement design over three months. | Mulberry-containing diets maintained intake and positive live-weight gain; digestibility declined relative to the conventional concentrate and declined further with Eupatorium inclusion. | https://doi.org/10.1016/j.livsci.2010.08.019 |
| 58 | Experiment 1 used 18 Dorper × thin-tailed Han crossbred ewes; experiment 2 used six rumen-cannulated ewes. | Mulberry leaf flavonoids, 2 g/head/day, compared with control diets. | Flavonoids improved nitrogen/NDF digestibility, reduced daily methane output, increased total VFA, decreased protozoa and methanogens, and increased Fibrobacter succinogenes. | https://doi.org/10.1111/asj.12556 |
| 59 | Fattening Hu sheep assigned to diets containing different proportions of whole mulberry. | Whole mulberry branch-and-leaf material at graded dietary proportions. | Reported outcomes include growth, apparent digestibility, serum antioxidant indices and rumen fermentation; later primary papers cite improved ADG at moderate whole-mulberry inclusion. |  |
| 60 | Xiangdong black goats received graded fermented-mulberry-leaf diets. | Fermented mulberry leaves at 10%, 20% and 30% inclusion levels. | Directly measured growth, serum indices, muscle amino acids and fatty acids; later primary reports cite higher ADG at 20-30% inclusion. |  |
| 61 | 160 healthy Hu sheep (19.12 ± 1.04 kg), four groups, five pens/group, eight sheep/pen. | Silage mulberry replaced peanut-seedling forage at 0%, 15%, 30% or 45% for 60 days. | Growth and slaughter performance were maintained. Cooking loss and shear force fell; selected unsaturated fatty acids increased; SFA decreased; at 30-45%, several amino acids, total amino acids and flavor-related amino acids increased. | https://doi.org/10.15933/j.cnki.1004-3268.2022.08.016 |
| 62 | Fifteen fattening Liuyang black goats, 3-4 months old and approximately 17 kg, divided among three treatments. | TMR pellets containing 0%, 15% or 20% mulberry leaf powder. | Direct growth, slaughter, serum and economic outcomes; published reports indicate improved feed efficiency and loin-eye area at mulberry inclusion. |  |
| 63 | Fattening Hu sheep in a graded replacement experiment. | Protein-mulberry silage used to replace whole-corn silage. | Measured growth performance plus nutrient digestion and metabolism, adding direct evidence on nutritionally balanced forage replacement. |  |
| 64 | 45 Xiangdong black goat rams, 5-6 months old, 18.2 ± 1.6 kg; 15 goats/group. | Mulberry silage replaced 0%, 50% or 100% of soybean-meal protein; 7-day adaptation + 55-day formal period. | Replacement increased DMI and ADG, altered serum antioxidant/lipid indices, VFA proportions and rumen bacterial composition. | https://doi.org/10.11686/cyxb2024426 |
| 65 | 120 healthy male Hu sheep (21.44 ± 2.31 kg), four groups, six replicates/group, five sheep/replicate. | Pelleted TMR containing 0%, 10%, 20% or 30% silage mulberry; 5-day adaptation + 60-day trial. | At 30%, ADG and intake increased; 20-30% reduced cooking/drip losses, increased T-AOC/SOD and lowered MDA; VFA responses changed at higher inclusion. | https://doi.org/10.15906/j.cnki.cn11-2975/s.2024120001-03 |
| 66 | Hu sheep feeding trial; the exact biological sample size was not confirmed from the accessible bibliographic record and is therefore not inferred here. | Silage forage mulberry incorporated into the diet; the study directly evaluated growth, slaughter performance and longissimus dorsi composition. | Directly measured growth/slaughter outcomes and muscle amino-acid and fatty-acid composition. The manuscript uses this study conservatively as evidence that muscle composition can respond to silage mulberry feeding. |  |
| 67 | 25 West African Dwarf (WAD) does in a 90-day feeding trial in Nigeria. | Graded replacement of Guinea grass with Morus alba foliage in diets containing cassava peel-based concentrate; the manuscript reports the greatest average daily gain at 75% mulberry forage. Exact treatment levels beyond those explicitly stated in the manuscript are not inferred here. | Directly measured protein intake, nutrient digestibility, nitrogen retention/balance and growth performance. The manuscript reports improved protein intake, digestibility and nitrogen retention with graded mulberry replacement, with the greatest average daily gain at 75% mulberry forage. Carcass and meat-quality endpoints were not assessed. | Anim. Res. Int. 2026, 23(1), 6942-6952. DOI not provided in the original manuscript. |

**Supplementary Table S1C. Mechanistic interpretation, strengths and limitations, and use in the manuscript.**

| **ID** | **Mechanism / interpretation** | **Main strengths & limitations** | **Manuscript use** |
| --- | --- | --- | --- |
| 1 | The core mechanism is free-radical lipid peroxidation through initiation, propagation, and termination, with oxidation rate influenced by fatty-acid unsaturation, oxygen, pro-oxidants such as heme/iron, and antioxidants. These mechanisms are well established biochemically, but their connection to mulberry feeding is inferential and must be supported by mulberry-specific animal studies. | Strengths include a clear biochemical explanation linking lipid oxidation to multiple dimensions of meat quality. Limitations for our review are that it is narrative, not mulberry-specific, not small-ruminant-specific, and does not quantify dietary dose-response effects. It should therefore not be used as direct evidence that mulberry improves goat or sheep meat. | Evidence rating: MODERATE SUPPORTING. Use this paper in the sections on 'Mechanisms linking mulberry feeding to meat quality' and 'Oxidative stability/meat quality'. It complements the direct goat/sheep studies by explaining why enhanced antioxidant status and altered fatty-acid composition could influence meat quality, but direct claims about mulberry must come from ruminant experiments. |
| 2 | The authors attribute the ability of mulberry to replace concentrate to its relatively high nutritive value, digestibility, protein supply, palatability, and mineral content. These mechanisms are supported indirectly by feed composition and digestibility data rather than by molecular or rumen-microbiome measurements. | Strengths are the graded 0-100% replacement design, iso-nitrogenous diets, 90-day feeding period, digestibility assessment, and slaughter measurements. Limitations include the small sample size per treatment, a single breed/sex/production environment, limited mechanistic measurements, and absence of detailed meat chemistry or sensory measurements. | Evidence rating: HIGH DIRECT RELEVANCE for feed replacement, intake, growth, digestibility, and carcass sections. It is particularly useful for the dose-response/evidence-map table and for showing that mulberry leaf meal can replace conventional concentrate in sheep. It should not be cited as evidence for fatty-acid, flavor, or detailed meat-quality effects because those outcomes were not measured. |
| 3 | The proposed mechanism is primarily nutritional: high crude protein/mineral supply and substantial ruminal degradability make mulberry leaf meal a viable supplement. This is directly supported for chemical composition and degradation kinetics, but not for downstream meat-quality mechanisms. | Strengths include direct chemical characterization and multiple incubation times for ruminal degradation. Major limitations are the absence of an in-vivo small-ruminant response in this particular study, use of only three fistulated cattle for in-sacco work, and lack of bioactive-compound standardization. It should not be used to infer growth or meat effects by itself. | Evidence rating: MODERATE-HIGH for the 'Nutritional characteristics/feeding value' section. Use it to support statements about crude protein, minerals, digestibility, and ruminal degradability of mulberry leaf meal, and pair it with direct sheep/goat feeding trials for biological outcomes. |
| 4 | The proposed mechanisms involve differences in forage versus concentrate lipid composition, rumen biohydrogenation, energy supply, intramuscular-fat deposition, physical activity, and diet-derived flavor precursors. These are largely synthesis-level explanations rather than mechanisms experimentally tested within the review itself. | Strengths are its direct focus on sheep/goat meat and its integration of growth, carcass, physicochemical, flavor, and fatty-acid outcomes. Limitations include a narrative rather than formal systematic design, substantial heterogeneity among source studies, and possible overlap with other contemporary reviews. It is not evidence specific to mulberry. | Evidence rating: HIGH SUPPORTING. Use it in the introductory meat-quality framework, the section on sources of heterogeneity, and interpretation of feeding-system effects. It provides context against which mulberry-specific responses can be compared, but mulberry claims should rely on direct Morus alba studies. |
| 5 | The authors propose that mulberry nutrients and bioactives (polysaccharides, flavonoids, phenolics and alkaloids) increase intake/growth and enhance antioxidant/immune status, while altering lipid metabolism and muscle fatty-acid deposition. Antioxidant changes were directly measured, but specific causal molecular pathways and rumen-mediated mechanisms were not tested. | Strengths include a true goat model, five graded inclusion levels, isoenergetic/approximately isonitrogenous diets, a 92-day period, and direct muscle amino-acid/fatty-acid analysis. Important limitations are only five goats per treatment, group-shared feed troughs that weaken individual feed-intake inference, no rumen measurements, no volatile/sensory testing, and limited carcass/physical meat-quality data. The authors themselves note the small group size. | Evidence rating: VERY HIGH DIRECT RELEVANCE. This should be one of the anchor studies in our review for dose-response, goat growth, antioxidant status, fatty acids, amino acids, and the discussion of optimal inclusion. Use caution when describing 'flavor': state that compositional indicators were improved, not that sensory flavor was directly demonstrated. |
| 6 | The mechanism is nutritional synchrony: mulberry provides fermentable nitrogen/protein and fermentable substrate that supports rumen microbial activity and fiber digestion in a low-quality rice-straw diet. This is supported directly by digestibility, VFA, ammonia, and microbial-N measurements. | Strengths include direct sheep rumen/digestibility measurements and a clear comparator diet. Limitations are the very small sample size (four sheep per treatment), short duration, absence of growth/carcass/meat endpoints, and dependence on a specific low-quality rice-straw basal diet that may limit generalization to intensive goat systems. | Evidence rating: HIGH DIRECT RELEVANCE for 'intake, digestibility and rumen fermentation'. Use it to establish that mulberry can function as a fermentable protein/energy supplement in sheep. Do not use it as direct evidence of meat-quality improvement. |
| 7 | The authors propose that MLP enhances nutrient digestibility and acetate supply, which stimulates rumen papilla/epithelial development and absorptive capacity. These links are partly demonstrated because fermentation and tissue morphology were measured. The reasons for poor performance at 45-60% are more inferential and may involve diet composition, palatability, or excessive replacement of concentrate ingredients. | Strengths are five inclusion levels, direct sheep data, repeated performance/digestibility measurements, and combined fermentation plus histological outcomes. Limitations include only eight animals per group, no meat outcomes, and substantial changes in concentrate composition across treatments, making it difficult to attribute every effect solely to mulberry bioactives. | Evidence rating: VERY HIGH for the mechanistic pathway 'mulberry -> rumen digestion/fermentation -> epithelial adaptation'. It is also crucial for the dose-response section because it shows that high inclusion can reduce intake and growth. Use the authors' practical recommendation of about 30% MLP in concentrate with appropriate context. |
| 8 | The authors link responses to the high protein, digestibility, palatability, and bioactive compounds of mulberry, together with altered rumen nutrient use and lipid metabolism. Digestibility/rumen changes and muscle fatty-acid changes were directly measured, but specific molecular pathways were not. | Strengths include a direct sheep model, four graded replacement levels, rumen, blood, carcass, intramuscular-fat and fatty-acid measurements in one trial. Limitations include eight animals per group, a relatively short finishing period, simultaneous replacement of a forage ingredient that changes overall diet composition, and no direct volatile/sensory analysis. | Evidence rating: VERY HIGH DIRECT RELEVANCE. Use as an anchor study for forage replacement, digestibility, rumen fermentation, carcass maintenance, intramuscular fat, and fatty-acid profile. The 24% treatment is particularly important when discussing an apparent intermediate optimum. |
| 9 | Proposed mechanisms include antioxidant radical scavenging, antimicrobial effects on rumen microbes, reduced methanogenesis, improved fiber degradation/VFA supply, and possible modulation of energy-homeostasis pathways by flavonoids. Some individual mechanisms are supported experimentally in cited studies, but the integrated pathway remains partly inferential. | Strengths are its direct focus on mulberry in ruminants and detailed mechanistic discussion. Limitations include narrative methodology, inclusion of mixed species and experimental contexts, variable mulberry preparations, and reliance on some in-vitro/non-production evidence. Importantly, this prior review means our new paper must add novelty rather than repeat a general 'mulberry is beneficial' narrative. | Evidence rating: VERY HIGH SUPPORTING/FOUNDATIONAL. Use for Introduction, nutritional/bioactive composition, rumen mechanisms, antioxidant mechanisms, and identification of historical research gaps. Our review should go beyond it by emphasizing small-ruminant meat quality, dose-response, newer 2021-2026 studies, evidence quality, and direct links from rumen/metabolism to carcass/meat outcomes. |
| 10 | The mechanism is directly supported in part: mulberry silage changed diet fiber/protein characteristics, rumen pH, VFA proportions, ammonia, bacterial community and rumen papilla morphology. However, because maize silage and soybean meal were changed together, the specific contribution of mulberry bioactives cannot be separated from altered nutrient composition. | Strengths include a relatively large sample, replicated pen design, graded replacement, rumen microbiota analysis and gastrointestinal morphology. A key confounder is simultaneous replacement of maize silage and adjustment of soybean meal, so treatment effects represent a diet package rather than a pure mulberry dose. Meat outcomes were limited, and microbial associations do not prove causality. | Evidence rating: VERY HIGH for rumen/gastrointestinal mechanisms and feasibility of mulberry silage in Hu lambs. Use together with the companion Wang et al. meat-quality paper to connect rumen changes with downstream carcass/muscle outcomes, while explicitly noting the diet-composition confounding. |
| 11 | The authors propose that fermentation reduces antinutritional factors and improves nutrient accessibility, while mulberry nutrients/bioactives influence lipid and amino-acid metabolism and intramuscular fat deposition. Some physiological/compositional outcomes were measured, but causal molecular pathways were not tested. | Strengths include a large starting sample, graded replacement, carcass assessment, serum biochemistry, pH/proximate composition, amino acids and fatty acids. The major limitation is confounding because both maize silage and soybean meal proportions changed with treatment. The study also lacks direct sensory/volatile analysis and mechanistic tissue measurements. Some favorable and unfavorable amino-acid shifts coexist, so conclusions should be balanced rather than uniformly positive. | Evidence rating: VERY HIGH DIRECT RELEVANCE. Use in growth, carcass, physicochemical meat, amino-acid and fatty-acid sections. It is especially valuable for showing that 20-40% replacement can be feasible while producing specific muscle-composition changes. Pair with Han et al. for rumen mechanisms. |
| 12 | The proposed mechanisms include radical scavenging, modulation of inflammatory pathways, antimicrobial changes in gut/rumen communities, effects on lipid/glucose metabolism, and bioactive-compound actions. The authors emphasize that whole mulberry materials are chemically complex, so mechanisms are often not attributable to a single compound. | Strengths are breadth and a useful catalogue of mulberry forms/bioactivities. Limitations are narrative methodology, mixed species, variable preparations, and substantial mechanistic evidence from laboratory animals rather than production ruminants. The authors explicitly identify the need for more work on individual monomer compounds and production animals. | Evidence rating: HIGH SUPPORTING. Use for the bioactive-compound/mechanism section and for identifying research gaps. It also helps demonstrate why our proposed review must be more focused on small-ruminant production-to-meat pathways and newer direct experiments rather than repeating a broad animal-production overview. |
| 13 | The mechanism is inferred from differences in energy density, forage lipid composition, grazing activity, fat deposition, and nutrient intake across systems. The meta-analysis demonstrates associations at the outcome level but does not directly test biochemical mechanisms. | Strengths include quantitative synthesis and a larger evidence base than individual trials. Limitations include heterogeneity among source studies, broad feeding-system categories that can mask specific diet ingredients, dependence on published data, possible publication bias, and restriction mainly to sheep rather than goats. It is not mulberry-specific. | Evidence rating: HIGH SUPPORTING. Use to benchmark mulberry outcomes against the broader effects of feeding systems and to justify why diet formulation, energy supply and production system are key sources of heterogeneity. It can strengthen the critical discussion but should not be used to estimate a mulberry-specific effect size. |
| 14 | The proposed mechanism combines a high-protein alternative feed source with mulberry bioactive compounds that may enhance antioxidant status and nutrient intake. Intake, digestibility, plasma chemistry and TAC/MDA were directly measured, but molecular and rumen mechanisms were not. | Strengths include recent direct goat evidence, individual housing, clear protein-replacement contrast, substantial sample size for a goat trial, digestibility, blood, carcass and amino-acid measurements. Limitations include only three dose levels, no rumen data, no direct flavor/volatile panel, mixed antioxidant signals, and the important internal inconsistency in the stated experimental duration. Full replacement also changes multiple diet ingredients, so effects are not a purified mulberry-compound test. | Evidence rating: VERY HIGH DIRECT RELEVANCE and one of the most current anchor studies. Use in feed-replacement, growth, digestibility, antioxidant, carcass and amino-acid sections. Explicitly mention the TAC/MDA trade-off and avoid reproducing the paper's inconsistent duration as a precise value unless clarified from an erratum/source. |
| 15 | The authors propose that mulberry nutrients and bioactive compounds regulate immune/oxidative status through metabolic and microbial pathways. Microbiota and blood changes were directly observed, but causal links between the specific taxa, immunity, and later meat quality were not demonstrated. | Strengths are direct sheep data, a clearly defined 60 g/day treatment, 42-day formal feeding and microbiota sequencing. Limitations include only 16 animals, a single dose, no rumen samples, no meat outcomes, and interpretation of fecal microbial changes as 'healthy' despite increases/decreases in taxa with context-dependent biological significance. Blood-enzyme changes also warrant cautious interpretation. | Evidence rating: HIGH for the physiology/microbiota supporting section, but not for meat-quality claims. Use it when discussing systemic and hindgut responses and the need to distinguish measured biological change from demonstrated production benefit. |
| 16 | The mechanism is directly related to ensiling ecology: Pediococcus drives lactic-acid production and pH reduction, while combined bacteria and wheat-bran substrate shape the microbial community and preserve/enhance antioxidant capacity. The feed-to-animal mechanism remains untested in this paper. | Strengths include direct characterization of fermentation acids, pH, ammonia-N, antioxidant activity and bacterial community. Limitations are absence of an animal validation phase, all treatments receiving wheat bran (so its contribution cannot be separated), and lack of nutrient digestibility/meat outcomes. Inoculated silage effects cannot be assumed to translate directly into goat meat quality. | Evidence rating: MODERATE-HIGH for 'Forms of mulberry feed and processing/ensiling'. Use it to explain why silage preparation and inoculants are sources of heterogeneity among feeding studies and why mulberry 'form' must be recorded in the evidence map. |
| 17 | The mechanism is substrate composition: differences in protein/fiber and interaction with basal TMR NDF alter microbial fermentation kinetics, VFA patterns and degradation. This is directly supported under in-vitro/in-situ conditions but requires in-vivo confirmation. | Strengths include a recent Morus alba comparison of leaf versus pomace, multiple fiber backgrounds, and combined gas/VFA/digestibility/degradation measurements. Limitations are only two cannulated cattle as donors, no small-ruminant in-vivo validation, and a high 50% substitution design that may not match commercial goat diets. Extrapolation to growth or meat quality is not justified. | Evidence rating: MODERATE SUPPORTING. Use in the nutritional-value and feed-form sections, especially to show that mulberry response depends on the fiber context of the basal ration. It is useful mechanistically but should not be counted as a goat/sheep meat-quality trial. |
| 18 | H-FABP expression was higher in selected muscle of the pellet group, and ADSL expression was higher in longissimus/biceps muscles; the authors link these genes to intramuscular fat and IMP deposition, respectively. Gene-expression associations were directly measured, but causality between expression and sensory quality was not experimentally manipulated. | Strengths include a direct goat model, comparison of feed-processing forms, digestibility, physical meat traits, fatty acids, amino acids, IMP and gene expression. Limitations include only five slaughter animals per group, no graded dose-response because all mulberry treatments were 40%, no volatile/sensory confirmation, and several simultaneous diet-processing differences that complicate attribution to mulberry itself versus pellet/fresh/fermented form. | Evidence rating: VERY HIGH DIRECT RELEVANCE and an anchor meat-quality study. Use in feed-form, growth/digestibility, tenderness/color, fatty acids, amino acids/IMP, and mechanism sections. It is especially important for the argument that processing form can be as important as inclusion level. |
| 19 | Mechanistic discussion links genotype, age, production system, muscle composition, intramuscular fat and postmortem properties to quality. These are general goat-meat mechanisms rather than evidence about Morus alba. | Strengths are direct focus on goat meat and broad coverage of quality dimensions. Limitations for our purpose are narrative methodology, no mulberry-specific synthesis, and substantial breed/production heterogeneity. It should be used for background definitions/baselines, not as proof of a mulberry effect. | Evidence rating: HIGH SUPPORTING. Use in the 'what meat quality means' framework, baseline goat-meat composition, and interpretation of fatty-acid/physicochemical outcomes. It complements direct mulberry-goat studies such as Luo, Long and Khattab. |
| 20 | The authors propose that fermentation improves mulberry feed properties and that altered intestinal fermentation/microbiota promotes gut health. Those gut changes were measured directly, but they are monogastric and cannot be transferred directly to rumen or goat-meat mechanisms. | Strengths include a large sample and graded dose design. The major limitation for our review is species relevance: pigs are monogastric, so cecal/intestinal mechanisms differ fundamentally from ruminant fermentation. It is also described as preliminary and did not measure meat quality. It should not be included in pooled small-ruminant conclusions. | Evidence rating: LOW-MODERATE SUPPORTING ONLY. Use, if needed, in a brief discussion of fermentation processing and general bioactivity, clearly labeled as non-ruminant evidence. It is optional and should not displace direct goat/sheep studies. |
| 21 | Mechanisms are mainly nutritional and production-system based: forage versus concentrate fatty-acid supply, rumen biohydrogenation, energy intake, fat deposition and physical activity. These are synthesis-level explanations. | Strengths include direct relevance to both sheep and goats and integration of growth, carcass and meat quality. Limitations are narrative methodology, heterogeneous source studies, and substantial conceptual overlap with Huang et al. (2023). When writing our review, avoid double-counting the same primary studies simply because they appear in both reviews. | Evidence rating: HIGH SUPPORTING. Use in the feeding-system framework, sources of heterogeneity, and fatty-acid/meat-quality interpretation. It should serve as contextual evidence rather than primary evidence for mulberry effects. |
| 22 | Mechanistically, diet and animal factors alter IMF and fatty-acid pools; postmortem lipolysis/oxidation then generates volatile compounds that contribute to species-specific flavor. These pathways are well supported across meat-science studies, but the step from mulberry feeding to flavor remains a hypothesis unless a mulberry trial measures volatiles/sensory outcomes. | Strengths include a recent, sheep/goat-specific synthesis and direct connection among IMF, fatty acids and flavor. Limitations are narrative design, no mulberry focus, and heterogeneous analytical methods for volatile compounds/sensory evaluation among the source literature. | Evidence rating: HIGH SUPPORTING. Use in the flavor section and mechanistic figure to explain how mulberry-induced changes in IMF/fatty acids could alter flavor. It is essential for preventing overclaiming: composition changes are flavor precursors, not direct proof of improved sensory flavor unless volatiles or sensory panels were measured. |
| 23 | Mechanistic evidence includes increased UCP3 and altered expression of hormone-sensitive lipase, acetyl-CoA carboxylase alpha, lipoprotein lipase, PPARgamma and NRF2-related pathways. These are directly measured gene-expression associations in pig muscle and provide plausible lipid/oxidative mechanisms. | Strengths include a large sample, graded dose, muscle fatty-acid profile and molecular endpoints. The decisive limitation is species: pigs lack rumen fermentation and have different lipid metabolism from goats/sheep. Therefore, molecular findings are hypothesis-generating for our ruminant review, not direct evidence. Diet ingredients also changed together as mulberry increased. | Evidence rating: MODERATE SUPPORTING ONLY. Use sparingly in the mechanism section when a ruminant study lacks molecular evidence, and label it explicitly as non-ruminant. Direct small-ruminant studies must remain the basis of conclusions about goat/sheep meat. |
| 24 | The proposed mechanism is that mulberry flavonoids selectively suppress or favor rumen bacterial groups, changing microbial protein and VFA production and thereby nutrient availability. Rumen fermentation and 16S changes were directly measured; however, predicted metagenomic functions and correlation networks do not prove causal microbial pathways. | Strengths include a true ruminant model, graded flavonoid doses, rumen fermentation and 16S profiling, and a relatively large sample for mechanistic ruminant work. Limitations include buffalo rather than sheep/goats, use of an extract rather than whole mulberry feed, predicted rather than directly measured metagenomic functions, and correlational links. No meat outcomes were studied. | Evidence rating: HIGH SUPPORTING MECHANISTIC. Use in 'rumen microbiome/fermentation mechanisms' to support biologically plausible flavonoid pathways, but clearly distinguish buffalo extract evidence from whole-leaf goat/sheep feeding evidence. |
| 25 | The study directly demonstrated that feeding regime changes the raw-muscle metabolome and selected volatile/hydrophilic compounds. The authors proposed that altered nutrient intake and lipid/amino-acid metabolism produce these chemical differences. However, the connection from individual biomarkers to perceived flavor/taste is largely inferential because the study did not perform sensory validation or aroma-activity quantification. GC-MS and UHPLC-QTOF-MS provide strong chemical-discrimination evidence, not proof of consumer-perceived flavor improvement. | Strengths include a four-diet comparison, direct mulberry-silage group, two complementary metabolomics platforms, quality-control samples and multivariate analysis. Limitations include only six animals per diet, untargeted/semiquantitative metabolomics, multiple-comparison complexity, several volatile results with relatively high FDR, raw rather than cooked meat, no sensory panel, and no causal mechanism. The exact diet proportions are in supplementary material and should be extracted before any quantitative dose analysis. The trial appears closely related in farm, age/body weight and feeding duration to other Tan-lamb silage studies from the same research group, so independence/possible cohort overlap should be checked before meta-analysis. | Evidence rating: VERY HIGH relevance for the flavor/metabolomics section but MODERATE certainty for claims of actual sensory improvement. This is our strongest direct mulberry-silage paper for volatile and taste-related muscle chemistry. Use it to show that diet can change hydrophilic taste precursors more readily than raw-meat volatiles and to introduce metabolomic biomarkers. Do not write that mulberry 'improved flavor' without qualification; state instead that it altered compounds associated with flavor/taste and that sensory validation is still required. |
| 26 | The authors proposed that mulberry phytochemicals altered the rumen microbial community and that selected bacteria may interact with host metabolism to enhance antioxidant and immune function. Correlations between taxa and host markers support this pathway, but the study did not manipulate the identified bacteria or demonstrate causality. The proposed reduction in ruminal biohydrogenation based on lower Ruminococcaceae UCG-010/Lachnospiraceae-related taxa is a hypothesis, not a directly measured flux. | Strengths include a controlled replacement of alfalfa silage, relatively large starting cohort, nutritionally similar diets, concurrent performance, serum and rumen-microbiome measurements, and clear microbiome statistics. Limitations include only six slaughtered animals per group for terminal outcomes, pen housing that complicates the independence of intake observations, 16S association rather than functional validation, no digestibility measurement, limited immune phenotyping, and no direct muscle fatty-acid/omics measurement. The authors themselves acknowledge that microbial functions require confirmation. | Evidence rating: HIGH relevance for the rumen-microbiota/antioxidant mechanism section and MODERATE-HIGH certainty for systemic antioxidant effects; LOW-MODERATE for direct meat-quality effects because those traits were mostly unchanged. Use this paper to support the pathway mulberry silage -> rumen microbial restructuring -> host antioxidant/immune associations, while explicitly calling the microbial mechanism associative rather than causal. It complements Ouyang et al. (2020), which measured liver antioxidant-gene expression, and Hou et al. (2025), which measured liver antioxidant enzyme activity. |
| 27 | The authors proposed that mulberry polyphenols/flavonoids increased antioxidant defense through ROS scavenging and regulation of antioxidant pathways, helping protect tissues and potentially improve meat quality. Gene-expression changes provide mechanistic support at the liver transcriptional level, but the study did not measure ROS flux, antioxidant enzyme activities in muscle, oxidative stability of meat, or causally link liver gene expression to tenderness/color changes. | Strengths include a five-level dose-response design, matched crude protein across concentrates, concurrent growth, blood, meat and gene-expression outcomes and orthogonal linear/quadratic tests. Major limitations are only three slaughtered lambs per group for meat/liver outcomes, a single breed, no rumen measurements, no muscle oxidative-stability assays, no flavor/fatty-acid analysis, and changing concentrate ingredient composition/energy with increasing MLP. The very small terminal n means meat-quality and gene-expression estimates should be interpreted cautiously. | Evidence rating: HIGH relevance but MODERATE certainty. This is one of the most useful direct sheep papers for dose-response and physicochemical meat quality. It supports a practical threshold: 15-30% MLP maintained growth, whereas 45-60% depressed intake/gain, even though tenderness/redness and antioxidant-gene expression improved. It should be central in the sections on dose-response heterogeneity, meat quality and antioxidant mechanisms, and contrasted with Cirne et al. (2018), where mulberry hay did not alter sensory or instrumental quality. |
| 28 | The authors proposed that the high α-linolenic-acid content of mulberry hay altered the dietary fatty-acid supply reaching the animal, while ruminal biohydrogenation limited transfer of unsaturated fatty acids to muscle. The proposed link between diet and meat fatty acids is biologically plausible and supported by the measured diet and meat profiles, but ruminal biohydrogenation itself was not measured. Changes in CLA were interpreted in relation to ruminal lipid metabolism rather than directly demonstrated mechanistically. | Strengths include a controlled three-level dose design, individual housing, a common slaughter weight, chemical characterization of diets, direct GC analysis of meat fatty acids and regression testing for linear/quadratic responses. Limitations include only 24 lambs from one breed and production setting, no rumen measurements, no direct oxidative-stability measurements, and no independent replication across farms or breeds. Importantly, this paper and Cirne et al. (2018) use the same 24-lamb feeding trial; they must not be counted as independent animal experiments in meta-analysis or evidence counts. | Evidence rating: HIGH relevance for the fatty-acid/nutritional-quality section and MODERATE overall certainty because of the modest sample size and single trial. It strongly supports the conclusion that mulberry hay can alter specific long-chain and omega-3 fatty acids without changing total muscle fat or cholesterol. It should be used in the sections on fatty-acid composition, nutritional value of meat, ruminal lipid-metabolism hypotheses and dose-response heterogeneity. It should be discussed together with Cao et al. (2024), which also reports favorable shifts in lipid composition, while noting that Cao measured subcutaneous adipose fatty acids rather than the same muscle endpoint. |
| 29 | The authors mainly interpreted the absence of treatment effects through normal postmortem pH decline and the resulting preservation of water-holding capacity, cooking behavior and tenderness. No direct molecular mechanism was measured. The central conclusion is one of equivalence/non-impairment rather than mechanistic improvement. | Strengths include direct physicochemical measurements, a graded inclusion design, individual animals as experimental units for meat traits, common slaughter weight and a consumer sensory panel. Limitations include modest animal sample size, only one breed and production setting, only 30 sensory consumers, no trained descriptive sensory panel, no volatile analysis, no oxidative stability measurements and no mechanism measurements. Crucially, this paper and Cirne et al. (2019) derive from the same 24-lamb trial, so they must be treated as one experiment with multiple outcome publications rather than two independent studies. | Evidence rating: HIGH relevance for physicochemical and sensory sections, with MODERATE certainty. It supports the conclusion that mulberry hay at up to 25% can be used without compromising pH, color, water retention, tenderness or consumer acceptability. It should be paired with the 2019 companion fatty-acid paper and contrasted with Ouyang et al. (2020), where increasing mulberry leaf powder improved redness and reduced shear force. The contrast is useful evidence that response depends on mulberry form, dose, basal diet and animal population. |
| 30 | The authors proposed that complementary nutrient profiles of corn straw and mulberry create positive associative fermentation effects, improving microbial protein/fermentation efficiency and intake, while mulberry bioactive compounds contribute to antioxidant responses. The fermentation synergy was directly tested in vitro. The causal chain from these in-vitro effects to liver antioxidant status and meat water retention was not directly demonstrated. | Strengths include a two-stage in-vitro/in-vivo design, explicit testing of associative effects, multiple mulberry proportions, balanced complete diets, direct meat-quality and antioxidant measurements, and a recent sheep model. Limitations include only 32 sheep, only four carcasses per treatment for meat/carcass outcomes, lack of rumen sampling in vivo, no fatty-acid/omics/sensory analysis, and the fact that the treatment is a corn-straw/mulberry mixture rather than a simple mulberry dose. Therefore, observed effects cannot be attributed exclusively to mulberry phytochemicals. | Evidence rating: HIGH relevance for feed-combination, fermentation, meat water-holding and antioxidant sections, but MODERATE certainty because terminal meat outcomes use n=4/group. Use it to support context-dependent synergy and the argument that mulberry can upgrade low-quality crop residues. It complements Ouyang et al. (2020) on meat tenderness/antioxidant responses and Wang & Luo (2021) on antioxidant physiology, while providing a distinct mechanism based on nutrient complementarity rather than microbiome association. |
| 31 | Proteomic analysis of subcutaneous fat identified hundreds of differentially expressed proteins and enrichment of lipid-metabolism/oxidation pathways. Targeted PRM included proteins such as FASN, FH, ACAA1, ECHS1, AKT2 and PCK2. The authors linked these protein changes to lower carcass fatness and altered fatty-acid metabolism. This is stronger mechanistic evidence than a purely phenotypic study, but the small proteomic sample (n=3/group), observational pathway enrichment and absence of functional perturbation mean causality remains incomplete. | Strengths include a direct corn-vs-mulberry silage comparison, a four-month intervention, pen replication, carcass fat measurement, targeted GC-MS fatty-acid analysis and proteomic/PRM integration. Limitations are only five slaughtered lambs per diet and three proteomic replicates, lack of rumen data, lack of growth/feed-efficiency reporting, and potential overinterpretation of pathway associations. Most importantly, the analyzed long-chain fatty acids were from subcutaneous adipose tissue, so the findings cannot be treated as equivalent to muscle fatty-acid composition. | Evidence rating: HIGH relevance for carcass fatness and lipid-metabolism mechanisms, MODERATE certainty because of small terminal and omics sample sizes. Use this paper in the carcass-fatness, lipid metabolism and mechanistic-omics sections, and as supporting evidence for favorable fatty-acid shifts. Pair it with Cirne et al. (2019), which provides direct longissimus muscle fatty-acid data, and clearly distinguish tissue type. |
| 32 | The authors proposed that mulberry feed form alters substrate availability and therefore rumen microbial community structure while maintaining functional fermentation stability. They also used PICRUSt to predict changes in microbial carbohydrate and lipid metabolic pathways and proposed potential links to host lipid metabolism. These pathway-level conclusions are inferential: PICRUSt predicts gene content from 16S profiles and does not directly measure microbial genes, transcripts, metabolites, or fluxes. Correlations such as Ruminococcus_2 with butyrate support association, not causation. | Major strengths are the exact White Cashmere goat model, comparison of ensiled versus sun-dried mulberry, multiple inclusion levels, a 60-goat starting cohort, 16S rRNA sequencing, qPCR for major rumen microbial groups, direct fermentation assays, rumen morphology, and serum lipid-related biomarkers. Major limitations include only three goats per dietary treatment for terminal sampling, pooling of some terminal results by feed form, one endpoint sampling time, absence of digestibility/growth/carcass/meat-quality outcomes, and reliance on PICRUSt predictions for microbial function. | Evidence rating: VERY HIGH relevance because it directly studies White Cashmere goats and practical mulberry feed forms. Certainty is MODERATE for rumen/community conclusions because terminal sampling was small. Use it in feed-form/processing, rumen fermentation and microbiota, metabolic/physiological responses, integrated mechanisms, heterogeneity, and evidence-quality sections. It should not be cited as proof of improved meat quality. |
| 33 | The authors proposed that mulberry nutrients and selenium could favor meat production, selenium enrichment, and lipid metabolism. However, the experiment did not measure rumen metabolism, gene expression, oxidative pathways, lipidomics, or causal intermediates. The mechanisms are therefore largely interpretive. Because the intervention used selenium-rich mulberry, effects cannot be attributed uniquely to ordinary mulberry phytochemicals versus selenium or their interaction. | Strengths include a direct goat feeding experiment, a White-goat breed, a defined 15% inclusion level, reported diet composition, direct slaughter and physicochemical measurements, longissimus muscle fatty-acid analysis, and muscle selenium measurement. Major limitations are terminal n=3 per group, only one inclusion level, selenium-rich rather than standard mulberry (a confound), a 45-day formal feeding period, no growth/digestibility/rumen/oxidative-stability/flavor/sensory outcomes, and multiple fatty-acid tests without a reported multiple-comparison correction. | Evidence rating: HIGH relevance to carcass/meat/fatty-acid scope, but MODERATE-LOW to MODERATE certainty for terminal outcomes because n=3 per group. Use it in carcass characteristics, physicochemical meat quality, fatty-acid composition, heterogeneity, and evidence-quality sections. It is especially valuable as contradictory evidence because it found little change in major fatty-acid classes and a significant increase in muscle water loss. |
| 34 | The proposed mechanism was built from convergent biochemical, metabolomic and transcriptomic evidence. Mulberry feeding reduced PUFA-peroxidation products and increased tryptophan-pathway metabolites; transcriptomic analyses identified differentially expressed genes associated with oxidative stress and immunity. FML additionally increased glycolytic intermediates and mannose-6-phosphate, while DML increased D-mannose. The authors proposed that reduced PUFA peroxidation plus enhanced tryptophan metabolism improved antioxidant defense, whereas FML strengthened immune function through glycolysis and the mannose-6-phosphate pathway. These pathways are supported by multi-omics associations and measured biomarkers, but they were not tested by pathway inhibition, gene manipulation or other causal interventions, so the mechanism remains strongly supported but not fully causal. | Strengths include a reasonably large starting cohort (54 sheep), nutritionally similar treatment diets, direct comparison of fermented versus dried leaves, repeated growth measurements, antioxidant assays in multiple tissues, immune markers, targeted metabolomics, RNA-seq and publicly deposited omics data. Important limitations are that only six sheep per group were slaughtered and some omics analyses used only 5-8 biological replicates; conventional rumen, carcass and meat-quality measurements were absent; several mechanistic conclusions rely on correlations/pathway enrichment; and the pen/replicate structure should be considered when interpreting feed intake. Most importantly, this paper shares the same experimental cohort with Cui et al. (2024), so the two papers must not be counted as independent animal trials. Study-independence note: IMPORTANT: This paper and Cui et al. (2024, Journal of Animal Science, skae076) are companion publications from the same 54-sheep experiment. They must be treated as one experimental cohort in evidence counts or any future quantitative synthesis. | Evidence rating: HIGH relevance for antioxidant, immune and integrated-mechanism sections, with MODERATE-HIGH certainty for the measured antioxidant and growth responses and MODERATE certainty for the specific molecular mechanisms. Use it in Sections 7 (antioxidant/metabolic responses), 13 (integrated mechanisms), 14 (feed-form heterogeneity) and 15-16 (evidence quality/future omics research). It provides strong mechanistic context for direct meat-quality studies such as Ouyang et al. and Luo et al., but should not itself be cited as proof of improved pH, tenderness, sensory quality or muscle fatty-acid composition. |
| 35 | The authors proposed two connected mechanisms. First, mulberry changed glycerolipid/phospholipid, ether-lipid and sphingolipid metabolism, potentially influencing immune-related complement/coagulation pathways. Second, mulberry promoted lipolysis, mitochondrial fatty-acid β-oxidation and oxidative phosphorylation, reducing tissue fat; DML additionally appeared to stimulate peroxisomal β-oxidation and produced stronger lipolysis than FML. The evidence is mechanistically strong because lipidomics, transcriptomics, biochemical markers and immunofluorescence converge, but causal pathway manipulation was not performed. The explanation that stronger DML lipolysis is due to its higher fiber content remains a hypothesis. | Major strengths are the integrated subcutaneous-fat lipidomics and transcriptomics, multiple lipid-metabolism biomarkers, tissue-specific measurements, direct comparison of FML versus DML and public RNA-seq data. Limitations include small terminal/omics sample sizes, absence of rumen data, absence of conventional meat sensory/physicochemical endpoints, and use of subcutaneous adipose lipidomics rather than a standard muscle fatty-acid profile. The strongest methodological caution is non-independence: this is the same 54-sheep feeding experiment as Cui et al. (2023), so treating the two publications as separate experiments would inflate the evidence base. Study-independence note: IMPORTANT: This paper re-analyzes the same 54-sheep experimental cohort used in Cui et al. (2023, Frontiers in Immunology, 13:1088850). The identical design and NCBI transcriptomic accession (PRJNA898816) confirm that these are companion publications, not independent feeding trials. | Evidence rating: HIGH relevance for lipid-metabolism and integrated-mechanism sections, with MODERATE-HIGH confidence that mulberry altered tissue lipid metabolism and MODERATE confidence in the detailed causal pathway interpretation. Use it in Sections 10 (supporting lipid-metabolism context), 13 (mechanisms), 14 (processing-form heterogeneity) and 15-16 (omics limitations/future research). Pair it with direct muscle fatty-acid studies such as Cirne et al. and Luo et al.; clearly state that Cui et al. measured wide-target lipids in subcutaneous fat and muscle crude fat rather than a conventional muscle fatty-acid profile. |
| 36 | The authors proposed that the high phenolic/flavonoid content of mulberry improves digestive enzyme/microbial activity, fiber degradation and nitrogen use, increasing VFA supply while reducing ammonia accumulation. They further proposed that antioxidant compounds support postpartum metabolic recovery, reproductive endocrine function and offspring growth. The measured HPLC phenolic profile, digestibility, VFA/ammonia, TAC and progesterone changes support parts of this pathway, but specific rumen microbial taxa, molecular signaling pathways and causal links between individual phenolics and reproductive outcomes were not directly demonstrated. Therefore, the mechanistic explanation is plausible but partly inferential. | Strengths include a 150-day practical dairy-goat intervention, individual feeding, characterization of mulberry phenolics by HPLC, simultaneous production/reproduction/economic endpoints, and a separate digestibility/rumen study designed to avoid pregnancy/lactation confounding. Major limitations are the very small digestibility/rumen sample (n=3 bucks per treatment), multiple reproductive proportion endpoints based on only 12 does per group, possible inflation of significance for categorical reproductive outcomes, lack of rumen microbiome or molecular validation, and absence of meat-quality measurements. The interpretation of 25% and 50% replacement must also recognize that diets differed in fiber and nutrient composition as concentrate was displaced. Study-independence note: This is a distinct study. It includes two linked experiments: a 36-doe production/reproduction trial and a separate 9-buck digestibility/rumen-fermentation trial. These animal sets must be distinguished when interpreting outcomes. | Evidence rating: MODERATE-HIGH relevance for feed-replacement strategy, digestibility/rumen physiology and economic/sustainability context, but LOW direct relevance to the meat-quality outcome of our review. Use it selectively in Sections 3-5 (mulberry composition, inclusion strategy, digestibility/rumen function), Section 7 (physiological/antioxidant context), Section 14 (dose and production-stage heterogeneity) and Section 16 (economic/practical research priorities). It should not be used to support claims about carcass traits, meat fatty acids, amino acids, tenderness or flavor. The small n=3 rumen trial should be explicitly acknowledged when weighting that evidence. |
| 37 | The paper supports a nutritional mechanism in which leaf maturity and physical form alter palatability and digestibility. These mechanisms are inferred from feed intake/digestibility data rather than demonstrated through rumen or molecular measurements. | Strengths are direct goat evidence and comparison of multiple mulberry forms. Limitations include the very small sample, crossover design, old analytical/reporting standards, and no rumen, carcass or meat-quality outcomes. Leaf maturity and diet formulation confound any simple conclusion that one mulberry percentage is universally optimal. | Evidence rating: HIGH DIRECT RELEVANCE for foundational goat feed-value evidence but MODERATE certainty because n was small. Use in Sections 3–6 and 14 to show that mulberry form and maturity materially influence intake, digestibility and growth; do not use it as meat-quality evidence. |
| 38 | The evidence directly demonstrates that ensiling can preserve mulberry as a palatable goat feed and that combining mulberry silage with a low-quality forage can improve nutrient intake and nitrogen retention. The mechanistic explanation is nutritional rather than molecular. | Strengths include direct goat evidence, explicit feed-preservation work and graded supplementation. Limitations include only five animals, Latin-square carryover risk, limited modern compositional characterization, and absence of production/meat endpoints. | Evidence rating: HIGH DIRECT RELEVANCE for feed preservation, intake and nitrogen utilization. Use in Sections 3–5 and 14 as foundational evidence that silage processing and replacement basis matter; do not extrapolate to meat quality. |
| 39 | The authors' interpretation is nutritional: relatively high protein, moderate fibre and high digestibility make mulberry capable of replacing part of conventional feed without impairing nutrient utilization. This is directly supported by feed composition/digestibility data. | Strengths are strong feed characterization and direct sheep digestibility evidence. Limitations are absence of production/meat outcomes and lack of standardization for modern bioactive marker compounds. | Evidence rating: HIGH for nutritional/feed-value Section 3 and supporting Sections 4–5. It strengthens the argument that mulberry is a credible forage/protein substitute but cannot establish effects on carcass or meat quality. |
| 40 | The results suggest that modest mulberry inclusion can alter the potentially degradable fraction of dietary DM, but the exact microbial mechanism was not tested. | Strength: direct rumen-cannulated lamb data with iso-nutrient diets. Major limitation: only three animals and a very narrow inclusion range; no production or product-quality outcomes. Latin-square estimates should not be interpreted as population-level proof. | Evidence rating: MODERATE-HIGH DIRECT MECHANISTIC relevance for Section 5 and Section 14. Use to support ruminal degradability context, not growth or meat outcomes. |
| 41 | The primary mechanism is nutrient substitution: mulberry supplies degradable protein and digestible nutrients to improve utilization of a low-quality straw diet. This is a nutritional inference rather than a directly measured molecular pathway. | Strengths are direct sheep evidence and a practical protein-replacement question. Limitations include the age of the study, limited accessible methodological detail, no standardized bioactive characterization and no meat-quality endpoints. Avoid extracting unverified numerical values. | Evidence rating: HIGH HISTORICAL DIRECT relevance for Sections 3, 5 and 6, but MODERATE certainty for quantitative synthesis until the full tables are available. Use qualitatively to show that mulberry can replace conventional protein supplement in low-quality forage systems. |
| 42 | A direct functional mechanism is partly demonstrated: a purified mulberry phytochemical intervention altered methane emission and nutrient digestion. However, methanogen abundance, microbial pathways and downstream muscle effects were not directly mapped. | Strengths include direct sheep data, respiration-based methane measurement and a defined phytochemical dose. Limitations include only 10 animals, change-over effects, purified extract rather than whole-feed exposure, and no rumen microbiome or meat-quality outcomes. | Evidence rating: HIGH SUPPORTING MECHANISTIC relevance for Sections 3, 5, 7 and 13. It supports biological activity of mulberry flavonoids but must not be treated as equivalent to feeding whole mulberry leaves. |
| 43 | The study directly supports nutrient-complementarity mechanisms in low-quality forage diets. The attempt to use Leucaena tannins to reduce ruminal protein degradation did not clearly create a major advantage at the tested ratio. | Strengths include a recent direct lamb trial, iso-energy/iso-protein formulation and concurrent intake, digestibility, nitrogen and rumen measurements. Limitations include 18 lambs, one basal feeding system and no product-quality endpoints. | Evidence rating: VERY HIGH DIRECT relevance for Sections 5–6 and 14. It updates the older rice-straw evidence and demonstrates that mulberry can replace a conventional urea-rice-bran supplement while improving intake/fibre digestibility without necessarily increasing ADG. |
| 44 | A direct rumen-ecology mechanism is supported because fermentation and microbial communities were measured in the same animals. Correlations between microbial taxa and digestibility/fermentation remain associative rather than causal. | Strengths include a comparatively large sheep cohort, recent study, two replacement levels, long formal period and bacterial+fungal profiling. Limitations include a heat-stress-specific context, fermented-feed confounding and no carcass/meat endpoints. | Evidence rating: VERY HIGH DIRECT relevance for Sections 4–6, 13–15. It substantially strengthens the review's recent evidence and shows that moderate fermented-mulberry replacement may be more favorable than a higher level under heat stress. |
| 45 | The demonstrated mechanism is nutritional substitution: high-protein, relatively low-fibre foliage with high digestibility can improve nutrient supply in tropical roughage systems. No direct microbial causal mechanism was measured. | Strengths include both sheep digestibility and cattle production phases and direct comparison with tropical grasses/cottonseed. Limitations for our review are species extrapolation (cattle support, not sheep/goat meat), modest n and absence of product-quality outcomes. | Evidence rating: HIGH SUPPORTING RUMINANT evidence for Sections 3–6. Use as cross-ruminant confirmation of feed value, not as direct small-ruminant or meat-quality proof. |
| 46 | The mechanism is directly supported at the digestive level: pelleted mulberry increased intake/digestibility and altered rumen fermentation while maintaining rumen pH. No microbiome sequencing was performed. | Strengths are controlled crossover measurements, rumen fistulation and total collection. Limitations include only four cattle, cross-species supporting status, possible period/carryover effects and no product endpoints. | Evidence rating: HIGH SUPPORTING RUMINANT relevance for Sections 4–5 and 13. It is useful for showing that physical processing into pellets can improve utilization of low-quality rice-straw diets. |
| 47 | The study demonstrates a processing/nutrient-synchrony mechanism: pelleting mulberry with urea provides fermentable nitrogen and substrate that can increase rumen bacterial abundance and roughage intake. The mechanism is digestive, not a direct meat-quality pathway. | Strengths include fistulated animals and microbial quantification. Limitations are only four cattle, urea confounding (not mulberry alone), Latin-square carryover risk and lack of product-quality outcomes. | Evidence rating: HIGH SUPPORTING RUMINANT evidence for Sections 4–5 and 13–14. Use to emphasize that feed processing and nitrogen supplementation materially alter the response to mulberry. |
| 48 | The mechanism is feed-preservation biology: fermentable glucose and LAB shift ensiling fermentation and microbial ecology, which can modify the quality of the feed presented to ruminants. This was directly demonstrated at silage level but not linked to animal or meat outcomes. | Strengths are controlled additive comparisons and 90-day silage analysis. Limitations are no in-vivo animals, no animal performance, and additive-specific effects that cannot be attributed to mulberry alone. | Evidence rating: MODERATE-HIGH FEED-LEVEL relevance for Sections 3–4 and 14. Use to explain why ensiling protocol is an important source of between-study heterogeneity. |
| 49 | The paper directly demonstrates that ensiling additives can change preservation quality and therefore the effective feed presented to animals. It does not demonstrate an animal rumen-to-meat mechanism. | Strengths are controlled silage processing and evaluation of both digestibility and aerobic stability. Limitations are feed-level evidence only and treatment effects that depend on additive type and storage conditions. | Evidence rating: MODERATE-HIGH FEED-LEVEL relevance. Use in Sections 3–4 and 14 to justify treating 'mulberry silage' as a heterogeneous intervention whose quality depends on inoculation/carbohydrate strategy. |
| 50 | The study directly links mulberry silage to rumen-community/fermentation shifts and a production output (milk). Correlations support, but do not prove, microbial mediation of propionate and milk responses. | Strengths include n=36, two inclusion levels and concurrent microbiome/fermentation/performance data. Limitations are cattle rather than small ruminants, branch-plus-leaf silage, and no meat endpoints. | Evidence rating: HIGH SUPPORTING RUMINANT relevance for Sections 4–5 and 13. It strengthens the general rumen-mechanism model but must be labelled as cross-species evidence. |
| 51 | The study directly demonstrates that a modest leaf supplement can shift rumen fermentation/microbiota without necessarily changing production. Microbial associations are not proof of causal mediation. | Strengths include direct rumen-community and blood measurements under practical dairy conditions. Limitations are only 12 cows, one mulberry dose, short experimental duration, cross-species evidence and no meat outcomes. | Evidence rating: HIGH SUPPORTING RUMINANT relevance for Sections 5, 7, 13 and 14. It is useful because it shows that microbial/fermentation changes can occur even when production remains unchanged. |
| 52 | The authors attribute benefits to improved diet nutritive value and nutrient utilization plus antioxidant-active mulberry constituents. Digestibility and antioxidant measurements support parts of this mechanism, but rumen-microbiome-to-muscle causality was not tested. | Strengths include a recent direct sheep trial, four graded inclusion levels and concurrent digestibility/growth/blood outcomes. Limitations include one breed/production setting, no detailed meat-quality endpoints and limited mechanistic depth. | Evidence rating: VERY HIGH DIRECT relevance for Sections 5–7 and 14. It is useful for current dose-response evidence showing favorable responses at 5–15% inclusion in a nutritionally balanced lamb ration. |
| 53 | Mechanism is conventional forage quality and ruminal/total-tract nutrient utilization; the study does not establish meat-quality or phytochemical mechanisms. | Strengths: direct controlled sheep digestibility comparison and foundational relevance. Limitations: no carcass, meat-quality, omics or sensory outcomes. | Use in feed-value and digestibility sections as foundational direct sheep evidence; do not use for meat-quality claims. |
| 54 | Provides quantitative context for dose-dependent production/immune responses but cannot establish a small-ruminant feed-to-meat mechanism. | Strengths: systematic search and quantitative synthesis. Limitations for this review: cross-species pooling, small number of studies in some outcomes and limited meat-specific endpoints. | Use in the Introduction to position novelty and in evidence-quality discussion; not a substitute for direct sheep/goat evidence. |
| 55 | Useful for defining the broader forage context and showing why a small-ruminant feed-to-meat synthesis remains distinct. | Strengths: recent and broad forage perspective. Limitation for the present question: not organized around sheep/goat meat-quality pathways or evidence hierarchy. | Use in Introduction novelty positioning and contextual discussion only. |
| 56 | Mechanism is conventional forage maturation: increasing structural material and changing protein availability modify ruminal disappearance and intestinal protein supply. | Strengths: controlled maturity comparison and direct degradation measurements. Limitations: not a production or meat-quality trial. | Use in feed-characteristics and heterogeneity sections; supports standardizing plant maturity rather than treating all mulberry leaves as compositionally equivalent. |
| 57 | Combines tree-forage protein substitution with ruminal fermentation effects; the Eupatorium co-intervention prevents attribution of all responses to mulberry. | Strengths: direct sheep feeding plus in-vitro validation. Limitations: mixed intervention and no meat-quality outcomes. | Use cautiously in feed-value/replacement and heterogeneity sections; not evidence that pure mulberry improves meat quality. |
| 58 | Direct evidence that mulberry flavonoids can modify methanogenesis and rumen microbial ecology, but purified-extract exposure is not equivalent to whole-leaf feeding. | Strengths: direct sheep data, separate digestion and cannulated-rumen experiments. Limitations: small n and purified extract. | Use in rumen and mechanistic sections to distinguish phytochemical effects from conventional nutrient replacement. |
| 59 | Integrates whole-plant nutrient supply, rumen fermentation and systemic antioxidant responses. | Strengths: direct sheep, multiple response domains. Limitation: reporting/accessibility in Chinese-language literature is less detailed in international indexing. | Use in intake/digestibility, growth, rumen and evidence-heterogeneity sections. |
| 60 | Provides direct evidence that fermented mulberry can affect goat muscle composition; mechanisms were not resolved with integrated rumen-to-muscle omics. | Strengths: direct goat, dose response, AA and FA endpoints. Limitation: Chinese-language reporting and limited sensory/mechanistic endpoints. | Use in growth, fatty-acid, amino-acid and heterogeneity sections. |
| 61 | Direct phenotypic evidence that nutritionally compatible silage replacement can alter tenderness/water loss and muscle nutrient composition; no complete rumen-to-muscle causal analysis. | Strengths: large n, multiple dose levels, carcass/meat/AA/FA endpoints. Limitation: single breed/production system. | Anchor evidence for meat quality, fatty acids and amino acids; substantially strengthens the former amino-acid evidence gap. |
| 62 | Effects combine mulberry ingredient composition with pelleted-TMR processing. | Strengths: direct goat and economic endpoint. Limitation: small n and no detailed meat chemistry/sensory assessment. | Use in growth/carcass, processing and economics/future-research discussion. |
| 63 | Supports the principle that response depends on the nutrient properties of the displaced silage rather than mulberry percentage alone. | Strengths: direct sheep nutrient-use experiment. Limitation: does not directly resolve sensory/meat-quality mechanisms. | Use in feed strategy, digestibility, growth and dose/replacement heterogeneity sections. |
| 64 | Provides direct rumen/host mechanism evidence for the protein-replacement design; because design characteristics closely match Khattab et al. (2026), it is treated as companion rather than independent replication. | Strengths: direct goat, n=45, rumen microbiome and antioxidant outcomes. Limitation: cohort non-independence with the companion publication. | Use in mechanisms and evidence-quality sections; do not double-count as independent replication. |
| 65 | Integrates production, meat water retention, antioxidant and rumen-fermentation endpoints in one relatively large experiment. | Strengths: large direct sheep trial, dose response and multiple connected endpoints. Limitation: no muscle AA/FA or sensory panel. | Use as recent anchor evidence for growth, physicochemical meat quality, antioxidant responses and rumen fermentation. |
| 66 | Direct phenotype/composition evidence; no complete causal rumen-to-muscle mediation experiment was established from the accessible record. | Strengths: direct sheep muscle AA/FA endpoints. Limitation: full methodological and bibliographic detail is less accessible internationally, so unverified numerical details are not inferred. | Use in Sections 6.2 and 6.3 to strengthen direct evidence for muscle fatty-acid and amino-acid responses. |
| 67 | Supports a nutritional forage-substitution mechanism through improved nutrient use and nitrogen retention; no direct rumen microbiome, molecular, carcass or meat-quality mechanism was tested. | Strengths: direct goat feeding trial, 90-day duration, n=25, and important geographic/breed extension to West African Dwarf does in Nigeria. Limitations: no carcass or meat-quality outcomes, and treatment-level details not explicitly provided in the original manuscript are not inferred. | Use in Section 8 (Sources of Response Heterogeneity) to broaden geographic representation and external-validity evidence; also supports the general intake/digestibility evidence base without being used as direct meat-quality evidence. |
